# Oral Microbiota and Clinical Outcomes in Allogenic Hematopoietic Stem Cell Transplantation: A Systematic Review

**DOI:** 10.3390/microorganisms14020308

**Published:** 2026-01-28

**Authors:** Jefferson Luiz da Silva, Alexandre Soares Ferreira Junior, Danielle Amanda Niz Alvarez, Larissa da Silva Souza, Luiza Dias Machado, Sarah Cantrell, Nelson Jen An Chao, Gislane Lelis Vilela de Oliveira

**Affiliations:** 1 Department of Genetics, Microbiology and Immunology, Institute of Biosciences (IBB), São Paulo State University, Botucatu 18618-970, São Paulo, Brazil; jefferson.l.silva@unesp.br (J.L.d.S.); alexandre.soares@unesp.br (A.S.F.J.); danielle.alvarez@unesp.br (D.A.N.A.); larissa.silva-souza@unesp.br (L.d.S.S.); luiza.d.machado@unesp.br (L.D.M.); 2 Duke University Medical Center Library, Durham, NC 27710, USA; sarah.cantrell@duke.edu; 3 Division of Hematologic Malignancies and Cellular Therapy, Department of Medicine, Duke University, Durham, NC 27710, USA; nelson.chao@duke.edu

**Keywords:** hematopoietic stem cell transplantation, stomatitis, microbiota, oral health, graft-versus-host disease

## Abstract

In patients undergoing allogeneic hematopoietic stem cell transplantation (allo-HSCT), emerging evidence suggests that the oral microbiota may serve as a predictive biomarker. We conducted a systematic review to provide a critical overview of oral microbiota research in the allo-HSCT setting. We searched PubMed, Embase, and Web of Science from inception to December 2025 to identify studies assessing the oral microbiota in allo-HSCT settings. We included all articles reporting detailed data on the oral microbiota in this context and conducted a qualitative synthesis. Risk of bias was assessed using the JBI critical appraisal tools. From 8160 initially identified records, 35 studies evaluating the oral microbiota in 1964 allo-HSCT patients were included. Of these, 27 studies (77%) assessed temporal oral microbiota dynamics and reported dysbiosis in the early post-transplantation period, followed by gradual recovery. Additionally, 27 studies (77%) evaluated the prognostic value of the oral microbiota, identifying associations with key clinical outcomes such as oral mucositis, overall survival, and graft-versus-host disease. Finally, substantial methodological heterogeneity was observed across studies, including differences in sampling techniques, sampling timepoints, and analytical strategies. This systematic review highlights the prognostic and therapeutic potential of the oral microbiota in allo-HSCT and underscores the need for standardized methodologies.

## 1. Introduction

In recent years, the oral microbiota has emerged as a potential predictive biomarker and therapeutic target in several clinical conditions, including the allogeneic hematopoietic stem cell transplantation (allo-HSCT) setting [1,2]. Allo-HSCT represents a particularly relevant clinical context, as it remains associated with substantial treatment-related morbidity and life-threatening complications [3,4,5,6]. Common complications following allo-HSCT include oral mucositis, infections, graft-versus-host disease (GvHD), and disease relapse [3,7]. For instance, acute GvHD is associated with significant morbidity, represents a leading cause of non-relapse mortality, and affects up to 70% of patients [3]. Additionally, oral mucositis, which is reported as the single most debilitating allo-HSCT side effect, may occur in up to 86% of patients [7]. Therefore, understanding oral microbiota disruptions in this setting may enable the development of predictive tools and targeted interventions to reduce these complications.

The oral microbiota represents the second most abundant microbial community in the human body, comprising more than 700 distinct microorganisms [1,8]. These microorganisms—including bacteria, fungi, and viruses—colonize multiple oral niches, such as the hard surfaces of the teeth, the tongue, saliva, and the oral mucosa [8]. Beyond merely inhabiting these sites, the oral microbiota plays a crucial role in human health through several mechanisms: (1) translocation to the gastrointestinal tract, potentially contributing to intestinal dysbiosis; (2) promotion of chronic systemic inflammation; and (3) production of metabolites that enter the systemic circulation and modulate host immune responses [1,8]. These mechanisms are particularly relevant in the allo-HSCT setting, where multiple factors act synergistically to promote epithelial barrier damage, immune reconstitution, and changes in the microbiota (antibiotics, conditioning regimen, total body irradiation, gastrointestinal tract diseases) [2,9,10,11].

Throughout the allo-HSCT course, several factors can significantly impact the oral microbiota [12]. Antibiotics are commonly used to prevent and treat life-threatening infections [13,14]. In addition, conditioning regimens aimed at eradicating residual malignant cells may promote mucosal injury [13,14]. As a result of these interventions and other contributing factors, specific patterns of oral microbiota disruption (dysbiosis) may emerge and have been linked to key clinical outcomes. For instance, distinct trajectories of oral microbiota diversity during allo-HSCT have been associated with an increased risk of oral mucositis, whereas changes in oral microbiota composition have been linked to chronic GvHD [2,9,15]. Despite the growing recognition of the oral microbiota as a relevant variable in the allo-HSCT setting, a comprehensive understanding of its overall dynamics and clinical associations remains lacking. Prior reviews in this field have either not been comprehensive or have focused on specific clinical outcomes, limiting our ability to understand the broader role of the oral microbiota in allo-HSCT [16,17]. Therefore, to address these gaps, we conducted a systematic review of the literature from inception to December 2025 to comprehensively evaluate the prognostic significance of the oral microbiota across a wide range of allo-HSCT-related outcomes, using a qualitative synthesis to integrate and contextualize these complex data for clinicians and researchers. As demonstrated in the following sections, the oral microbiota undergoes significant changes throughout allo-HSCT and plays an important role in the development of key clinical outcomes.

## 2. Materials and Methods

### 2.1. Study Design and PICO Framework

The study design is a systematic review reported in concordance with Preferred Reporting Items for Systematic Reviews and Meta-Analyses guidelines (PRISMA) [18]. The systematic review protocol was registered with the International Prospective Register of Systematic Reviews (PROSPERO; registration number CRD42021282558) [19]. This systematic review also followed the population, intervention/exposure, comparator, and outcomes (PICO) framework for the development of our main research question (What is the prognostic role of oral microbiota features in the allo-HSCT setting?) [20]. The population of interest was patients undergoing allo-HSCT. The exposure comprised features of oral dysbiosis (decreased diversity indices, altered microbiota composition, and domination events). The comparator was patients undergoing allo-HSCT without these dysbiosis features. The outcomes included any clinically relevant endpoints reported by the studies, including but not limited to oral mucositis, overall survival, aGvHD, cGvHD, relapse, and infections.

### 2.2. Search Strategy

A comprehensive literature search was developed and conducted by a medical librarian (S.C.) with input from all authors. We searched Medline (PubMed), Embase (Elsevier), and Web of Science (Clarivate) from database inception to 7 September 2022. The search included a mix of keywords and subject headings representing stem cell transplantation and microbiota (see Appendix A). No restrictions were placed on date or language in the search strategy. Editorials, comments, and notes were excluded from the search. The search strategy was peer-reviewed by another experienced medical librarian using a modified PRESS checklist [21]. A search update was conducted on 12 January 2025, including Medline (PubMed) and Embase (Elsevier). A final search update was performed on 15 December 2025 across all three databases. The full, reproducible search strategies for all included databases are in the Appendix A. Additional references were identified through the examination of previous reviews addressing oral microbiota in the context of allo-HSCT [16,17].

### 2.3. Study Selection and Eligibility Criteria

The identified studies were uploaded into Covidence systematic review software (Veritas Health Innovation, Melbourne, VIC, Australia) [22]. All titles and abstracts were screened by two independent reviewers (J.L.d.S., A.S.F.J., D.A.N.A., and L.S.S.) to determine their suitability for a full-text review. Any conflicts were resolved through discussion or by a binding vote from a third independent reviewer.

The inclusion criteria for studies were different according to the screening stage. At the title and abstract stage, the following criteria were used: (1) investigations involving patients undergoing allo-HSCT, and (2) studies reporting data on the microbiota (any site). Because some studies do not clearly specify in the abstract whether oral microbiota were assessed, during the title and abstract screening, we included studies evaluating any type of microbiota (intestinal, nasal, skin, blood, and other sites). In the full-text review stage we followed the same screening method as described above; however, only studies explicitly reporting oral microbiota data were retained. Exclusion criteria comprised: (1) non-human studies, (2) studies with incomplete or non-extractable oral microbiota data, (3) studies evaluating only intestinal, nasal, blood, or skin microbiota, (4) studies regarding fecal microbiota transplantation, (5) studies including patients with autologous HSCT, and (6) publications in languages other than English (resource limitations). The risk of bias was assessed by two independent reviewers (J.L.d.S. and D.A.N.A.) using the Joanna Briggs Institute Critical Appraisal Tools checklist (see Appendixes B and C for details) [23]. These checklists assess the methodological quality of articles with a total score generated from questions answered as “yes, no, unclear, or not applicable”. Given the heterogeneity of the included studies, the descriptive nature of this systematic review, and the fact that it is not an intervention-based review, the GRADE approach was not used to formulate recommendations.

### 2.4. Data Extraction

Data were extracted independently by two independent reviewers (J.L.d.S. and A.S.F.J.) into a predefined table. Both reviewers met initially and pilot-tested the extraction process for consistency. Once 100% agreement was achieved, each reviewer extracted the data independently. Any discrepancies were resolved through discussion until consensus was reached. Extracted variables included the primary author and year of publication, number of patients, demographic characteristics (age, sex, and primary indication for allo-HSCT), methodology for DNA sequencing, α-diversity and β-diversity indices and respective values, methods for bacterial taxonomic identification, and reported associations between oral microbiota features and clinical outcomes.

### 2.5. Data Synthesis

The included studies were synthesized qualitatively, and findings were organized thematically according to the primary clinical outcomes reported in association with the oral microbiota. These outcomes included oral mucositis, overall survival, GVHD, disease relapse, and other relevant clinical endpoints.

Studies were additionally classified into three categories based on oral microbiota profiles, following a conceptual template originally developed for intestinal microbiota [3,14]. The three categories were: (1) studies evaluating associations between oral microbial diversity and clinical outcomes, (2) studies assessing associations between oral microbiota composition and clinical outcomes, and (3) studies examining associations between oral microbial domination and clinical outcomes [3,14].

Data from the included articles were extracted and organized in summary tables to facilitate cross-study comparisons. When available, numerical values for diversity indices (e.g., Shannon, Simpson, Chao1) were reported and compared descriptively across different timepoints (pre- and post-transplantation) and clinical subgroups (e.g., patients with vs. without oral mucositis). Owing to substantial heterogeneity in study design, sample size, sequencing approaches, and analytic approaches, conducting a formal meta-analysis was not feasible. When statistical measures of association were reported (e.g., hazard ratios, *p*-values, confidence intervals), they were synthesized narratively and interpreted within the broader context of the evidence. Whenever possible, particular emphasis was placed on studies using multivariate models to adjust for potential confounders (e.g., age, conditioning intensity, antibiotic exposure), given their increased methodological rigor.

## 3. Results

### 3.1. Included Studies

**Figure 1:**
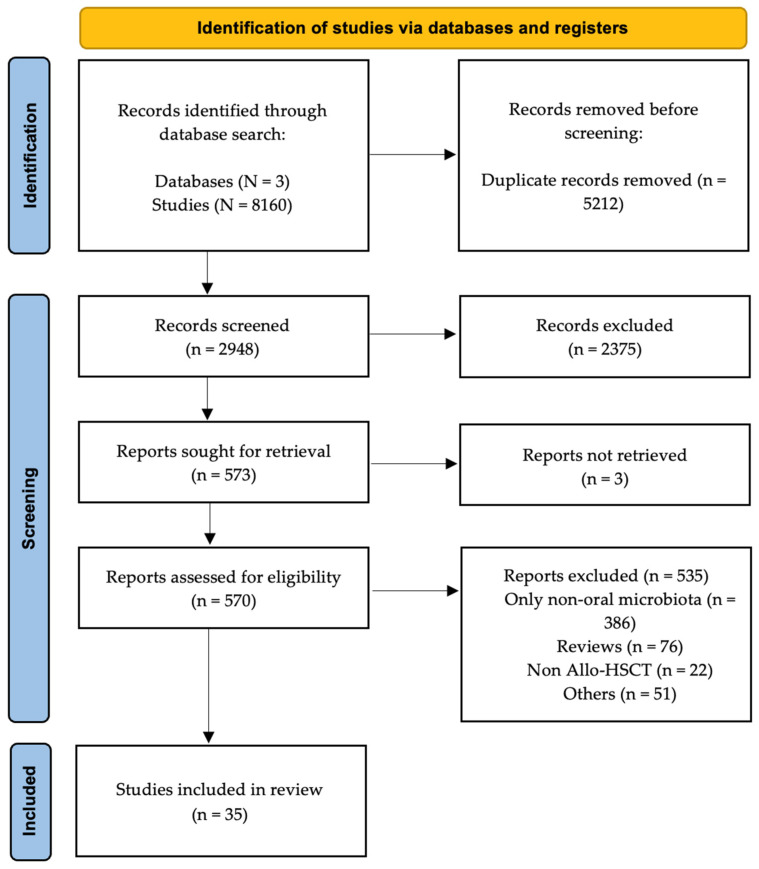
Study PRISMA flow diagram. There were 8160 references imported for screening and 5212 duplicates removed. The number of studies screened against title and abstract was 2948. Of these, 2375 studies were excluded. Of all the full-text studies assessed (n = 570), 535 studies were excluded: 386 evaluating only non-oral microbiota, 76 reviews, 22 non-allo-HSCT articles, and 51 for other reasons. Therefore, the final number of studies included was 35.

The initial and updated search strategies identified a total of 8160 records (see Figure 1). After removal of duplicates (n = 5212), 2948 studies remained for title and abstract screening. Of these, 573 met the inclusion criteria for full-text assessment. Three studies were not retrieved. A total of 535 studies were subsequently excluded, most commonly because they evaluated only non-oral microbiota (72%; n = 386), were review articles (14%; n = 76), or did not involve patients undergoing allo-HSCT (4%; n = 22). Reference searching in prior reviews did not identify any additional articles (see Supplementary Table S1) [2,10,13,15,24,25,26,27,28,29,30,31,32,33,34]. Therefore, 35 studies fulfilled all eligibility criteria and were included in the qualitative synthesis, providing data from 1964 patients who underwent allo-HSCT. Among these 35 studies, five were identified through the final updated search strategy. The risk of bias assessment is shown in Supplementary Tables S2A,B. Among the 28 studies included in the quality review (seven abstracts excluded), only one (4%) reported sufficient data to achieve the maximum score in the risk of bias assessment.

### 3.2. Patient Demographics and Oral Microbiota Methodologies

Among the 35 included studies, 25 (71%) exclusively reported data from adult patients [2,9,10,11,13,15,32,33,34,35,36,37,38,39,40,41,42,43,44,45,46,47,48,49,50], four (11%) focused solely on pediatric populations [31,51,52,53], and three (9%) included both adult and pediatric patients (see Supplementary Table S3) [12,54,55]. The remaining three studies (9%) did not provide information regarding patient age [56,57,58]. Information regarding biological sex was reported in 22 studies [2,9,10,11,13,15,31,32,34,35,36,38,39,40,45,46,47,48,49,50,54,58]. Across these studies, 554 patients (55%) were male and 451 (45%) were female. The remaining studies did not provide sex-related demographic data. Twenty-eight studies including 1439 patients reported data regarding underlying diagnosis [2,9,10,11,12,13,15,31,32,33,34,36,38,39,40,41,44,45,46,47,48,49,50,51,52,54,55,56]. Most of these patients had leukemia (59%, n = 834) or lymphoma (8%, n = 110).

Several aspects of oral microbiota methodologies varied across studies, including sample collection technique, sampling timepoints, and microbiota analysis methods (see Supplementary Table S3). Five main oral microbiota sampling techniques were used across the included studies: oral swab (18 studies) [2,9,11,12,13,31,34,36,37,40,45,46,47,54,55,56,57,58], saliva/oral rinse (14 studies) [9,15,32,38,39,41,42,43,44,48,49,50,51,52], dental biofilm collection (6 studies) [10,35,36,38,49,50], oral brushing (2 studies) [33,35], and gingival crevicular fluid sampling (3 studies) [36,38,50]. One study did not report its sampling technique [53]. Almost all studies (n = 30; 86%) collected samples before the allo-HSCT procedure [2,9,10,11,12,15,31,32,33,34,35,36,37,38,40,41,43,44,47,49,50,51,52,53,54,55,56,57,58]. However, post-transplantation sampling timepoints varied: four studies (11%) collected samples during aplasia [2,10,36,56], 10 (29%) at engraftment [2,10,12,33,35,36,51,52,55,56], 15 (43%) at later timepoints (>day + 60) [12,15,31,33,36,37,40,48,49,50,51,52,54,55,57], and 26 (74%) at other distinct moments (including mucositis onset, cGvHD onset, or fever onset) [11,12,13,15,31,32,33,35,36,37,38,39,40,41,43,44,45,46,47,48,49,50,51,53,55,57]. Heterogeneity was also observed in the methodologies used for microbiota analysis. The most common approach was 16S rRNA sequencing (57%, 20 studies) [2,9,10,11,12,13,15,31,33,36,37,41,42,43,44,48,53,55,56,58]. Other methodologies included culture-based techniques (17%, 6 studies) [39,45,46,51,52,54], shotgun metagenomic sequencing (11%, 4 studies) [40,49,50,57], real-time polymerase chain reaction (6%, 2 studies) [32,38], human oral microbe identification microarray (HOMIM; 3%, 1 study) [35], and restriction fragment length polymorphism analysis (RFLP; 3%, 1 study) [47].

### 3.3. Oral Microbiota Dynamics over the Allo-HSCT

**Figure 2:**
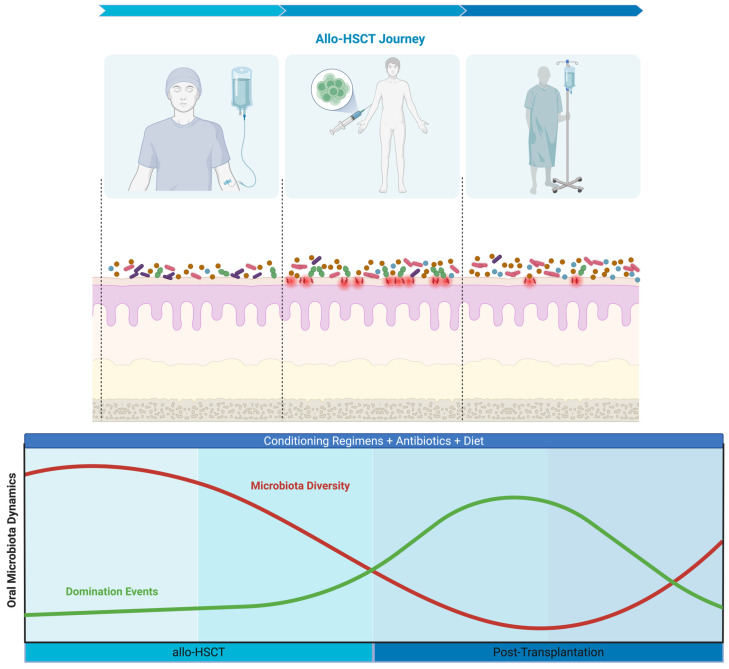
Dynamics of Oral Microbiota Over the Allo-HSCT Journey. Allo-HSCT = Allogeneic hematopoietic stem cell transplantation. Created with BioRender.com, Soares Ferreira Junior A. (2026) https://BioRender.com/gtn5lve (accessed on 26 January 2026).

Of the 35 included studies, 27 (77%) evaluated the temporal dynamics of the oral microbiota throughout the allo-HSCT course. These studies reported data from 1720 patients. The most frequently investigated microbiota characteristics were: (1) microbiota diversity (67%; n = 18/27) [2,9,10,11,12,13,15,31,33,36,40,41,42,43,44,49,50,55], (2) microbiota composition (78%; n = 21/27) [2,10,11,12,13,15,31,32,33,35,36,38,39,44,45,46,50,51,52,54,55], and (3) oral microbiota domination events (19%; n = 5/27) [2,9,10,13,36]. The overall patterns of change in these three microbiota characteristics are illustrated in Figure 2.

Although these alterations in the oral microbiota may be influenced by multiple factors, in the allo-HSCT setting, key factors driving these changes appear to include demographic characteristics (e.g., age, biological sex, and body mass index) [2,9,15,50,54], antibiotic exposure [9,10,13,33,34,36,45,48], comorbidities (e.g., underlying disease and renal impairment) [2,12], and host immune responses (see Supplementary Table S4) [31,48].

#### 3.3.1. Dynamics of Oral Diversity and Microbiota Composition over the Allo-HSCT

Among the 18 studies evaluating microbiota diversity, all (100%) assessed α-diversity, and five (28%) evaluated β-diversity throughout the allo-HSCT course (see Supplementary Table S5) [2,9,10,11,12,13,15,31,33,36,40,41,42,43,44,49,55]. Overall, the majority of studies (89%; n = 16/18) consistently demonstrated a significant decline in oral α-diversity during the first 30 days following allo-HSCT, followed by a gradual recovery approaching baseline levels around day +100 [2,9,10,11,12,15,31,33,36,41,42,43,44,49,50,55]. When compared with baseline samples (collected prior to allo-HSCT), α-diversity was significantly reduced at multiple timepoints, including during aplasia (n = 7) [2,10,15,36,42,43,44], at engraftment (n = 9) [2,10,12,15,31,33,36,41,55], and at D + 30 (n = 2) [12,36]. Although only a limited number of studies provided data at later post-transplantation timepoints (>D + 90), available evidence indicates a partial recovery by D + 100, with a return to baseline levels by D + 360 or D + 450 [15,33]. Similar to changes observed in α-diversity, the studies assessing β-diversity reported significant shifts in oral microbiota composition over the course of allo-HSCT [9,11]. These findings are further supported by the 21 studies (60%) that specifically investigated temporal changes in microbiota composition during allo-HSCT (see Supplementary Table S5) [2,10,11,12,13,15,31,32,33,35,36,38,39,44,45,46,50,51,52,54,55]. Although numerous taxa demonstrated temporal variability, clinically meaningful alterations included an increased relative abundance of *Streptococcus*, *Enterococcus*, *Actinomyces*, and *Solobacterium* (see Section 3.4) [2,10,31,37,56].

#### 3.3.2. Dynamics of Oral Domination Events over the Allo-HSCT

Among the 27 studies evaluating oral microbiota dynamics, only five (19%) investigated oral domination events during allo-HSCT (see Supplementary Table S5) [2,9,10,13,36]. These studies collectively included 161 patients and demonstrated that oral domination is a frequent microbiota feature, occurring in 59% to 100% of patients. Overall, all studies reported an increase in domination events following allo-HSCT, with the most commonly implicated genera including *Enterococcus* [10,36], *Rothia* [2,10], *Lactobacillus* [10,36], *Staphylococcus* [10,13,36], *Streptococcus* [2], and *Neisseria* [2].

### 3.4. Oral Microbiota and Clinical Outcomes

**Figure 3:**
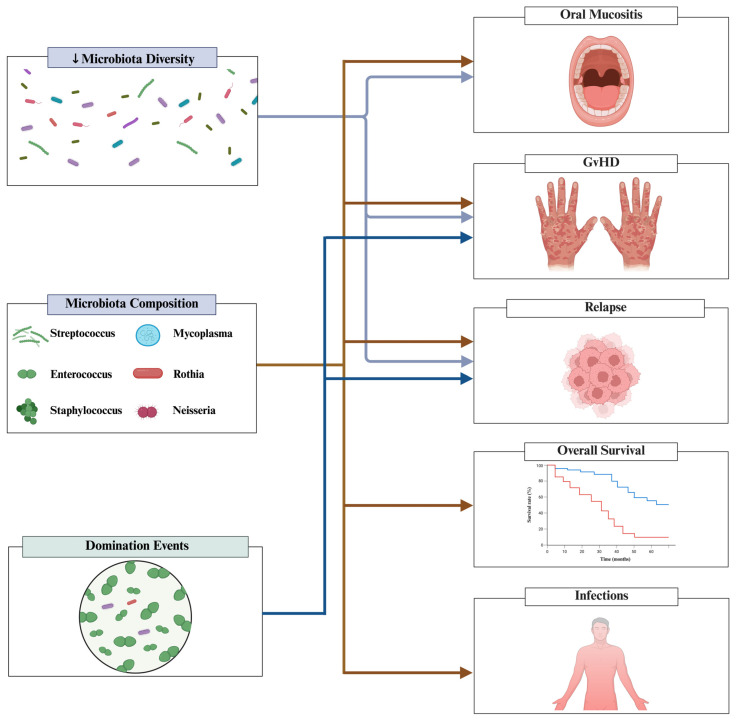
Associations between oral microbiota and clinical outcomes in the allo-HSCT setting. Allo-HSCT = Allogeneic hematopoietic stem cell transplantation; aGvHD = acute graft-versus-host disease. Created with BioRender.com, Soares Ferreira Junior A. (2026) https://BioRender.com/yhtif3k (accessed 26 January 2026).

Among the 35 included studies, 27 (77%)—encompassing 1772 patients—evaluated associations between the oral microbiota and clinical outcomes [2,9,10,11,12,13,15,31,32,35,36,37,39,40,41,42,43,44,47,48,50,53,54,55,56,57,58]. The most frequently evaluated clinical outcomes were: (1) oral mucositis (56%; n = 15 studies with 1276 patients) [2,9,11,12,15,32,41,42,43,44,47,50,54,55], (2) aGvHD (26%; n = 7 studies with 389 patients) [2,10,13,31,48,53,54], (3) overall survival (22%; n = 5 studies with 538 patients) [2,13,37,42,44], (4) cGvHD (22%; n = 6 studies with 244 patients) [2,15,40,48,57,58], and (5) relapse (7%; n = 2 studies with 60 patients) [2,56]. Overall, these studies showed significant associations between microbiota diversity, microbiota composition, and domination events and key clinical outcomes, including oral mucositis [11,15,41,42,43,44,50,55], overall survival [13,37], aGvHD [10,31,53], cGvHD [2,15,40,57,58], and relapse (see Figure 3) [2,56].

#### 3.4.1. Oral Diversity and Clinical Outcomes

Among the 35 included studies, 14 (40%) comprising 1071 patients evaluated associations between oral microbiota diversity and clinical outcomes (see Table 1) [2,9,10,11,15,41,42,43,44,48,50,53,56,58]. The clinical outcomes most frequently assessed were: (1) oral mucositis (64%; 9/14 studies; 927 patients) [2,9,11,15,41,42,43,44,50], (2) cGvHD (29%; 4/14 studies; 154 patients) [2,15,48,58], (3) aGvHD (29%; 4/14 studies; 113 patients) [2,10,48,53], (4) overall survival (21%; 3/14 studies; 398 patients) [2,42,44], and (5) relapse (14%; 2/14 studies; 60 patients) [2,56]. Additional outcomes assessed in single studies included progressiosn-free survival (PFS), non-relapse mortality (NRM), bronchiolitis obliterans, and febrile neutropenia [15,48]. Overall, most studies did not identify significant associations between oral microbiota diversity at single timepoints and the evaluated clinical outcomes (see Table 1). However, six studies reported that patients who developed oral mucositis experienced significantly greater reductions in oral microbiota diversity throughout the allo-HSCT course [9,15,41,42,43,44]. Additionally, one study found that patients who developed aGvHD exhibited a distinct diversity trajectory over time when compared with those who did not develop the disease, although specific details were not reported [53]. Also, in another study evaluating cGvHD, patients who later developed this complication had a lower Shannon diversity index both before (*p* = 0.001) and after allo-HSCT (*p* = 0.0001) [58]. Similarly, one study demonstrated that patients who later developed cGvHD showed a divergent diversity pattern relative to those without this complication [15]. Finally, two studies showed that lower pre-transplantation diversity was associated with increased risk of relapse [2,56]. Taken together, these data highlight the potential of monitoring oral microbiota diversity throughout allo-HSCT as a prognostic biomarker for predicting clinical outcomes. Both static and temporal changes in oral microbiota diversity appear to be important indicators of prognosis.

#### 3.4.2. Oral Microbiota Composition and Clinical Outcomes

Among the 35 included studies, 24 (69%) comprising 1688 patients evaluated associations between oral microbiota composition and clinical outcomes (see Table 2) [2,9,10,11,12,13,15,31,32,35,37,39,40,41,42,43,44,47,50,54,55,56,57,58]. The clinical outcomes most frequently assessed were: (1) oral mucositis (58%; 14/24 studies; 1246 patients) [9,11,12,13,15,32,41,42,43,44,47,50,54,55], (2) overall survival (12%; 3/24 studies; 170 patients) [2,13,37], (3) aGvHD (21%; 5/24 studies; 336 patients) [2,10,13,31,54], (4) cGvHD (21%; 5/24 studies; 201 patients) [2,15,40,57,58], (5) relapse (8%; 2/24 studies; 60 patients) [2,56], and (6) other clinical outcomes (21%; 5/24 studies; 384 patients) [2,13,35,39,54]. Additional outcomes evaluated included PFS [2], NRM [2], infections [13,39,54], fever of unknown origin [54], and respiratory manifestations [35].

Of the 14 studies evaluating the impact of microbiota composition on oral mucositis, 12 (86%) reported significant associations between specific microbial profiles and this outcome [9,11,12,15,32,41,42,43,44,47,50,55]. Six of these studies identified bacterial taxa present before allo-HSCT that were predictive of subsequent oral mucositis [11,41,42,43,44,50]. These taxa included *Kingella*, *Atopobium*, *Rothia*, *Bergeyella*, *Fusobacterium*, *Gemella*, *Haemophilus*, *Prevotella*, *Selenomonas*, *Oribacterium asaccharolyticum*, and *Streptococcus* [11,41,42,43,44,50]. There were also 11 studies that identified significant changes in microbiota composition over the course of allo-HSCT that were associated with the development, severity, or recovery of oral mucositis [9,11,12,15,32,41,42,43,44,47,55]. Key bacterial taxa reported in these studies included *Mycoplasma*, *Rothia*, *Lactobacillus*, *Enterococcus*, *Staphylococcus*, *Streptococcus*, *Neisseria*, *Prevotella*, and *Methylobacterium* (see Table 2 for details).

Regarding the three studies evaluating associations between microbiota composition and overall survival, one reported no significant associations between the abundance of five genera (*Enterococcus*, *Lactobacillus*, *Mycoplasma*, *Staphylococcus*, and *Solobacterium*) and survival outcomes [2], whereas the other identified that *Streptococcus*, *Enterococcus*, and *Actinomyces* were significantly increased in patients who died within 1–5 years post-transplantation [37]. The remaining study reported a significant association between overall survival and the presence of specific bacterial taxa, including *Staphylococcus haemolyticus* (*p* = 0.009), *Ralstonia pickettii* (*p* = 0.02), or either organism (*p* = 0.003) [13]. Notably, the presence of *Staphylococcus haemolyticus* and/or *Ralstonia pickettii* remained independently associated with overall survival in multivariable analysis (adjusted HR = 2.5; 95% CI, 1.0–6.4; *p* = 0.04) [13].

While three studies found no significant associations between the assessed genera or species and aGvHD [2,13,54], two additional studies reported notable findings [10,31]. Increased abundance of *Streptococcus* prior to conditioning was associated with higher risk of aGvHD, whereas increased *Veillonella* abundance was associated with lower risk [10,31]. Moreover, using a machine learning model, aGvHD severity was predicted based on the abundance of three oral ASVs: ASV 568 (*Actinomyces* sp.; *p* < 0.001), ASV 226 (*Prevotella melaninogenica*; *p* < 0.001), and ASV 500 (*Pseudopropionibacterium propionicum*; *p* < 0.001) [31].

Among the five studies evaluating associations between microbiota composition and cGvHD, four reported significant findings. One study showed that increased *Enterococcus* abundance in samples collected before conditioning and at engraftment was associated with a higher incidence of cGvHD [2]. Another study found that, at the time of cGvHD onset, patients exhibited increased relative abundance of *Veillonella parvula* (*p* adjusted = 0.06) and *Streptococcus salivarius* (*p* adjusted = 0.06) [40]. Moreover, in another study, two bacterial families were more prevalent in patients with cGVHD (*Staphylococcaceae* and *Enterobacteriaceae*) [58]. Finally, one study identified increased abundance of multiple bacterial and fungal species in patients who developed cGvHD [57]. With regard to relapse, in both studies assessing this outcome, increased presence of *Solobacterium* was associated with a higher risk of relapse [2,56].

Finally, among the five studies evaluating associations with other clinical outcomes, two identified significant results. One study reported that positive *Klebsiella pneumoniae* cultures were significantly associated with infectious events [54], and another demonstrated significant compositional differences between patients who developed versus did not develop respiratory manifestations [35].

Overall, although some conflicting findings were observed, across all major clinical outcomes at least one aspect of oral microbiota composition was significantly associated with clinical prognosis. These studies indicate that both static features (such as the presence of specific taxa at a given allo-HSCT timepoint) and dynamic features (temporal changes in the overall microbiota profile) may serve as potential prognostic markers.

#### 3.4.3. Oral Domination Events and Clinical Outcomes

Among the 35 included studies, associations between oral domination events and clinical outcomes were assessed in four studies (121 patients; see Table 3) [2,10,36,56]. The clinical outcomes evaluated were: (1) overall survival (two studies, 60 patients) [2,56], (2) aGvHD (two studies, 60 patients) [2,56], (3) cGvHD (one study, 30 patients) [2], and (4) other outcomes (three studies, 91 patients) [2,36,56]. The additional outcomes reported included infections [36], PFS [2,56], and NRM [2].

In both studies evaluating overall survival, domination by any genus was associated with reduced overall survival; however, this association did not remain significant in multivariable analyses [2,56]. Similarly, in the two studies assessing aGvHD, domination by any genus was not significantly associated with this outcome [2,56]. Nevertheless, *Enterococcus* domination was significantly associated with both aGvHD and severe aGvHD [2]. In the single study examining cGvHD, no significant association with domination by any genus was reported [2]. In contrast, both studies investigating relapse demonstrated a significant association between domination by any genus and increased relapse risk [2,56]. Regarding PFS, two studies observed that domination by any genus was significantly associated with inferior PFS [2,56].

## 4. Discussion

In this systematic review evaluating the oral microbiota in the context of allo-HSCT, we identified three major findings. First, available evidence suggests that allo-HSCT is associated with oral dysbiosis in the early post-transplantation period, followed by gradual recovery. Second, among oral microbiota changes, the diversity trajectory, microbiota composition, and domination events appear to have significant prognostic value. Finally, despite the emerging role of the oral microbiota in allo-HSCT settings, available studies demonstrate substantial heterogeneity across multiple methodological dimensions of microbiota research.

It is interesting to note our findings demonstrating that allo-HSCT is associated with significant oral dysbiosis. This oral microbiota imbalance seems to result from a combination of several factors, including demographic characteristics (e.g., age, biological sex, and body mass index) [2,9,15,50,54], antibiotic exposure [9,10,13,33,34,36,45], comorbidities (e.g., underlying disease and renal impairments) [2,12], and host immune responses (see Supplementary Table S4) [31]. For instance, in a study including 25 patients, biological sex significantly influenced β-diversity dynamics during allo-HSCT (oral mucosa: R^2^ = 0.04; adjusted *p* = 0.014), whereas older age and overweight were associated with a slower decline in *α*-diversity over time (estimate = 0.029; *p* = 0.033 and estimate = −0.060; *p* = 0.029, respectively) [9]. In the same study, greater antibiotic exposure during allo-HSCT was associated with more pronounced shifts in oral microbiota composition (oral mucosa: R^2^ = 0.05; adjusted *p* = 0.001) [9]. Antibiotic exposure was also associated with oral dysbiosis in a study of 31 allo-HSCT patients [36]. In this cohort, greater cumulative antibiotic exposure correlated with reduced diversity stability (CGF: *p* = 0.0172; OM: *p* = 0.0015; and SB: *p* = 0.0467), and glycopeptide use was significantly associated with decreased compositional stability (SB: *p* = 0.0235) [36]. Finally, in another study including 29 patients, immune system markers showed significant correlations with oral microbiota composition. CD4^+^Th17^+^ T-cell counts were positively correlated with members of the *Flavobacteriaceae*, *Prevotellaceae*, *Veillonellaceae*, and *Neisseriaceae* families, whereas NK cell counts were positively correlated with *Actinomyces odontolyticus* (ASV 422) and *Veillonella parvula* (ASV 546) [31]. Taken together, these findings suggest that, in allo-HSCT recipients, both host-related and transplant-related factors contribute to oral dysbiosis. This is particularly relevant, as identifying such key determinants may guide future research toward targeted intervention strategies aimed at modulating the oral microbiota throughout the allo-HSCT. However, it should be acknowledged that most studies included in this systematic review are limited by small sample sizes and/or methodological constraints in oral microbiota characterization. Therefore, future investigations with larger cohorts and more robust analytical approaches are desired.

Another key finding of this systematic review is the emerging prognostic relevance of the oral microbiota (see Table 1, Table 2 and Table 3). Although most research in the allo-HSCT setting has traditionally focused on the intestinal microbiota, the oral microbiota has now been recognized as an additional key site with potential predictive value [2,9,11,15]. In addition, the oral microbiota has been associated with specific allo-HSCT clinical outcomes, such as oral mucositis, which has been less extensively investigated in intestinal microbiota research [2,9,11,12,15,32,41,42,43,44,47,50,54]. Indeed, oral mucositis was the most frequently evaluated clinical outcome (56%; n = 15 studies with 1276 patients) among those included in our systematic review and was also the one with the most consistent findings, with studies reporting similar associations between oral microbiota alterations and this outcome. In the allo-HSCT setting, oral microbiota fingerprints associated with oral mucositis include both (1) temporal trajectories of diversity throughout the transplantation course [9,15,41,42,43,44] and (2) shifts in oral microbiota composition over time [9,11,12,13,15,32,41,42,43,44,47,50,55]. Although less thoroughly explored and with more heterogeneous findings, other important allo-HSCT clinical outcomes potentially linked to these two fingerprints include overall survival [2,13,37], aGvHD [2,10,31,53,54], cGvHD [2,15,40,57,58], and relapse risk [2,56]. Finally, we also identified that oral domination events may be another fingerprint with prognostic value [2,10,36,56]. Nevertheless, the divergent findings regarding the prognostic value of the oral microbiota in the allo-HSCT setting may be partly explained by the limited statistical power of existing studies and the substantial methodological heterogeneity across microbiota research. For example, some studies did not use multivariable analyses capable of accounting for potential confounders, such as antibiotic exposure or dietary changes (see Supplementary Table S4). Therefore, future studies should explore the potential clinical utility of dynamic oral microbiota monitoring during allo-HSCT using larger cohorts and robust, standardized methodological approaches.

Indeed, this substantial heterogeneity across multiple methodological dimensions of microbiota research emerged as one of the most important findings of this systematic review. This heterogeneity is problematic for several reasons. First, it hampers accurate comparability across studies, limiting our ability to conduct large-scale meta-analyses and to understand microbiota composition dynamics (genera and species) as well as the role of the oral microbiota in allo-HSCT. Second, it acts as a barrier to identifying confounders that may be influencing the variation observed across studies. Finally, because such heterogeneity can generate markedly different findings, it may lead to inaccurate associations and misinterpretation of microbiota fingerprints. As a result, there is a risk of falsely concluding that certain associations have prognostic significance when, in reality, they do not. All these risks were further highlighted by three studies included in this systematic review demonstrating different findings based on sampling site [35,36,50]. In a study including 16 allo-HSCT patients, differences in sample collection sites (plaque vs. saliva vs. buccal brushing vs. tongue brushing) were analyzed using HOMIM [35]. The number of positive probes varied by collection site—saliva (25.25; SD ± 9.97), tongue (24.27; SD ± 9.23), plaque (21.34; SD ± 11.03), and buccal specimens (19.33; SD ± 7.21) [35]. Additionally, the trajectory of positive probes over the allo-HSCT course also varied according to collection site [35]. Whereas only small differences were observed when comparing pre- and post-transplantation samples for tongue, plaque, and buccal specimens, saliva samples showed a significant change over the allo-HSCT period (*p* < 0.02) [35]. Similarly, in another study including 31 allo-HSCT patients, oral samples were collected from three different sites: (1) gingival crevicular fluid (GCF), (2) oral mucosa, and (3) supragingival biofilm (SB) [36]. In this study, significant differences across several microbiota features were observed when samples from different oral collection sites were analyzed, including: (1) microbiota composition (PERMANOVA: GCF vs. oral mucosa, *p* = 0.001; GCF vs. SB, *p* = 0.002; oral mucosa vs. SB, *p* = 0.018), (2) microbiota resilience (GCF showing higher diversity resilience than OM and SB), and (3) microbiota diversity (distinct trajectories depending on the collection site) [36]. Taken together, these studies and others highlight the potential errors that may arise when the sampling site is not standardized. It is important to note that this is only one of many variables that remain unstandardized in allo-HSCT microbiota research. Thus, there is a need for a standardized and unified microbiota methodology, spanning sampling timepoints, sequencing approaches, big-data analytics, and bioinformatic pipelines for data analysis.

Only through standardized microbiome research will it be possible to determine the true prognostic significance of the oral microbiota in the allo-HSCT setting. With a clear understanding of its prognostic value, findings from oral microbiota research may then be translated into clinical practice. Such translation into the allo-HSCT setting may occur through different strategies. First, as in other fields, the oral microbiota may be used to improve the diagnostic accuracy of allo-HSCT-related complications [59,60]. For example, in a study including 47 patients with oral squamous cell carcinoma, a machine learning model based on oral microbiota profiles was developed to diagnose this condition. This model achieved diagnostic accuracies ranging from 96% to 98% depending on the sampling site [59]. Oral microbiota may also be used as a prognostic stratification tool [61]. In a study including 1040 patients with indeterminate pulmonary nodules, an oral microbiota signature was associated with subsequent cancer development [61]. In this study, a model based on saliva samples achieved an area under the curve (AUC) of 0.887 (95% CI, 0.865–0.918) and identified the following taxa as predictive of cancer: *Actinomyces*, *Rothia*, *Streptococcus*, *Prevotella*, *Porphyromonas*, and *Veillonella* [61]. Finally, the oral microbiota may also be used to predict response to therapy or even serve as a therapeutic target [62]. For instance, in a study including 36 patients with breast cancer, a specific oral microbiota signature was associated with responsiveness to chemotherapy. Using oral microbiota biomarkers, a model with an AUC of 77.3% (95% CI, 60.5–94.2%) was developed, highlighting the potential role of the oral microbiota as a variable for anticipating treatment response. These studies highlight the potential clinical utility of the oral microbiota in the allo-HSCT setting. However, before translation into clinical practice, larger studies using standardized methodologies are required to clarify the prognostic role of the oral microbiota in allo-HSCT settings.

The primary strength of this study is its extensive search strategy and clinical perspective, which provide a comprehensive overview of oral microbiota research in the allo-HSCT setting. Previous reviews have not used a systematic review methodology to capture all available evidence and/or were limited to a single clinical endpoint [16,17]. Additionally, this study not only focused on sequencing methodologies but also incorporated relevant findings from studies that used culture-based and other analytical approaches. This study, however, has some limitations. First, a number of included studies did not report key demographic variables, such as sex and gender, and several did not achieve the maximum score in the methodological quality assessment. Second, the substantial heterogeneity across studies in several dimensions of microbiome research prevented meaningful comparisons and limited the ability to synthesize findings effectively. This heterogeneity is also problematic because it may lead to misinterpretation of the true prognostic role of the oral microbiota in the allo-HSCT setting. For instance, prior studies have highlighted important quantitative and qualitative differences between 16S rRNA gene sequencing and shotgun metagenomic approaches, which may result in divergent microbiota profiles and limit cross-study comparability [63,64]. Moreover, several of the included studies did not acknowledge or adjust for potential confounders (antibiotic exposure, immune reconstitution, conditioning regimen, demographics) that may influence the associations between oral microbiota and clinical outcomes identified in this systematic review. Additionally, the limited number of mechanistic studies hampers our ability to determine causal relationships, making it difficult to discern whether oral dysbiosis is a driver or a consequence of disease. Finally, most studies included in this review were based on small patient cohorts, which limits statistical power and reduces the ability to identify true prognostic associations between oral microbiota and allo-HSCT clinical outcomes. Notwithstanding these limitations, this systematic review provides valuable insights into the potential role of oral microbiota research in the allo-HSCT setting and underscores the urgent need for higher-quality studies using standardized microbiota methodologies.

## 5. Conclusions

Although this systematic review highlights the potential role of the oral microbiota as a predictive biomarker and therapeutic target in the allo-HSCT setting, the absence of high-quality evidence and standardized methodologies still limits our understanding of its true clinical relevance. Our findings reveal the substantial heterogeneity across microbiota studies in this field, underscoring the need for future research using standardized microbiota methodologies.

## Figures and Tables

**Table 1:** Clinical Implications of Oral Diversity Over the Allo-HSCT.

Outcome	Author, Year N	Sample Timing	Findings
**Oral Mucositis** [2,9,11,15,41,42,43,44]	Bartha 2025 [9] 25	Before Allo-HSCT After Allo-HSCT	**Before Allo-HSCT** Patients with ulcerative mucositis exhibited similar *α*-diversity compared to those without ulcerations (saliva: values NR, *p* value NR; oral mucosa: values NR, *p* value NR). **After Allo-HSCT** Patients with ulcerative mucositis exhibited similar *α*-diversity compared to those without ulcerations (values NR, *p* value NR). **Before and After Allo-HSCT** Patients with ulcerative mucositis had higher decline in *α*-diversity (estimate: −0.232, *p* = 0.041).
	Bruno 2022 [11] 30	Before Conditioning Oral Mucositis Onset Oral Mucositis Healing	**Before Conditioning vs. Oral Mucositis Onset** Similar *α*-diversity (values NR; *p* = 0.11). **Before Conditioning vs. Oral Mucositis Healing** ↓ *α*-diversity (values NR; *p* = 0.019). **Oral Mucositis Onset vs. Oral Mucositis Healing** Similar *α*-diversity (values NR; *p* = 0.21). **All Samples** *β*-diversity significantly differed between timepoints (*F* = 1.23; *p* = 0.015). **Before Conditioning** Patients who developed oral mucositis exhibited Shannon diversity indices comparable to those who did not develop the condition (values NR; *p* = 0.81). When stratified based on the median Shannon index (high vs. low), patients also exhibited similar oral mucositis cumulative incidence (*p* = 0.81).
	De Molla 2021 [2] 30	Before Conditioning At Aplasia At Engraftment	**Pre-conditioning** No association was observed between oral microbial diversity and the occurrence of oral mucositis (*p* = 1.00). **At Aplasia** No association was observed between oral microbial diversity and the occurrence of oral mucositis (*p* = 1.00). **At Engraftment** No association was observed between oral microbial diversity and the occurrence of oral mucositis (*p* = 1.00).
	Laheij 2022 [15] 50	Before Allo-HSCT (8 weeks to days before) Weekly during Hospitalization D + 90 D + 180 D + 360 D + 450	**Before Conditioning** There was no difference in oral microbial diversity at baseline between individuals who did and did not develop ulcerative oral mucositis (PERMANOVA, *p* > 0.001; Mann–Whitney U test, *p* > 0.05). **All Samples** There was a significant effect of oral mucositis on bacterial diversity (*p* = 0.028). Bacterial diversity was lower when oral mucositis was present. At D + 21, the decrease in microbial diversity was significantly greater in patients with oral mucositis compared with those who did not develop it (*p* = 0.0032).
	Shouval 2019 [41] 184	D − 7 to D − 1 D0 to D + 6 D + 7 to D + 13 D + 14 to D + 20 D + 21 to D + 34	**All Samples** Patients with grade 2–4 oral mucositis exhibited a greater reduction in *α*-diversity over time compared with those without mucositis or with only mild disease (*p* value NR).
	Shouval 2020 [43] 184	Weekly from D − 7 to D + 34	**All Samples** Patients with grade 2–4 oral mucositis exhibited a more pronounced decline in *α*-diversity over time compared with those with mild or no mucositis.
	Shouval 2020 [44] 184	D − 7 to D − 1 D0 to D + 6 D + 7 to D + 13 D + 14 to D + 20 D + 21 to D + 27 D + 28 to D + 34	**D** − **7 to D** − **1** **Grade 1–2 vs. Grade 3–4 Oral Mucositis** Oral β- and α-diversity were similar irrespective of future mucositis severity (*p* value NR). **D** − **7 to D + 13** Patients who developed severe oral mucositis exhibited a reduction in α-diversity (*p* < 0.01) and an increase in β-diversity (*p* < 0.05).
	Shouval 2020 [42] 184	NR	**All Samples** Severe oral mucositis was associated with a reduction in *α*-diversity over time (*p* < 0.01).
	Gem 2024 [50] 56	Baseline D + 7 D + 14 D + 21 D + 28 D + 84	**Baseline** **Subgingival Plaque** There was a weak but statistically significant correlation between α-diversity and total oral mucositis score at D +7 (r = −0.32; *p* = 0.03); however, this association was no longer significant after adjustment for multiple comparisons. **Baseline** **Shannon Index and Total Mucositis Score at D + 7** Saliva: r = −0.19, *p* = 0.18. Supragingival Plaque: r = −0.16, *p* = 0.28. Subgingival Plaque: = −0.32, *p* = 0.025. **Baseline** **Shannon Index and Total Mucositis Score at D + 14** Saliva: r = −0.15, *p* = 0.31. Supragingival Plaque: r = −0.04, *p* = 0.77. Subgingival Plaque: r = −0.21, *p* = 0.15. **Baseline** **Shannon Index and Total Mucositis Score at D + 21** Saliva: r = −0.04, *p* = 0.85. Supragingival Plaque: r = −0.06, *p* = 0.78. Subgingival Plaque: r = −00.02, *p* = 0.91. **D + 14** **Shannon Index and Total Mucositis Score at D + 14** Saliva: r = 0.007, *p* = 0.96. Supragingival Plaque: r = 0.10, *p* = 0.50. **D + 14** **Shannon Index and Total Mucositis Score at D + 21** Saliva: r = −0.12, *p* = 0.59 Supragingival Plaque: r = −0.11, *p* = 0.58.
**Overall Survival** [2,42,44]	De Molla 2021 [2] 30	Before Conditioning	**Low vs. High Diversity** No significant differences in OS were observed between patients with high and low diversity (54% vs. 57%; HR 0.96, 95% CI 0.33–2.89, *p* = 0.96).
	Shouval 2020 [42] 184	NR	*α*-diversity was not associated with overall survival (details NR).
	Shouval 2020 [44] 184	D − 7 to D − 1 D0 to D + 6 D + 7 to D + 13 D + 14 to D + 20 D + 21 to D + 27 D + 28 to D + 34	**Peri-engraftment Samples** Peri-engraftment α-diversity was not associated with overall survival (*p* = 0.89).
**aGvHD** [2,10,48,53]	De Molla 2021 [2] 30	Before Conditioning At Aplasia At Engraftment	**Low vs. High Diversity** No significant differences in aGvHD were observed between patients with high and low diversity (43% vs. 62%; HR 1.77, 95% CI 0.66–4.81, *p* = 0.26). **Before Conditioning** No association was observed between oral microbial diversity and the occurrence of aGvHD (*p* = 0.45). Similarly, no significant association was observed with severe aGvHD (*p* = 0.16). **Aplasia** No association was observed between oral microbial diversity and the occurrence of aGvHD (*p* = 1.00). Similarly, no significant association was observed with severe aGvHD (*p* = 1.00). **Engraftment** No association was observed between oral microbial diversity and the occurrence of aGvHD (*p* = 1.00). Similarly, no significant association was observed with severe aGvHD (*p* = 1.00).
	Heidrich 2021 [10] 30	Before Conditioning At Aplasia At Engraftment	**Before Conditioning** **Low vs. High Diversity** No significant differences in aGvHD were observed between patients with high and low diversity (HR 0.68; 95% CI 0.26–1.78; *p* = 0.43). **At Aplasia** **Low vs. High Diversity** No significant differences in aGvHD were observed between patients with high and low diversity (HR 0.88; 95% CI 0.33–2.31; *p* = 0.79). **At Engraftment** **Low vs. High Diversity** No significant differences in aGvHD were observed between patients with high and low diversity (HR 0.92; 95% CI 0.33–2.58; *p* = 0.87).
	Parco 2019 [53] 10	D + 2 D + 16 D + 24	**All Samples** Patients who developed GvHD exhibited a distinct diversity trajectory throughout the allo-HSCT course compared with those who did not develop GvHD (*p* value not reported).
	Brehm 2025 [48] 43	Not Clear	There were no significant differences in β- and α-diversity between individuals with and without aGvHD (NR).
**cGvHD** [2,15,48,58]	De Molla 2021 [2] 30	Before Conditioning At Aplasia At Engraftment	**Low vs. High Diversity** No significant differences in cGvHD were observed between patients with high and low diversity (30% vs. 7%; HR 4.79, 95% CI 0.56–40.8, *p* = 0.15). No association was observed between oral microbial diversity and the occurrence of cGvHD (*p* = 0.16). **At Aplasia** No association was observed between oral microbial diversity and the occurrence of cGvHD (*p* = 1.00). **At Engraftment** No association was observed between oral microbial diversity and the occurrence of cGvHD (*p* = 1.00).
	Laheij 2022 [15] 50	Before Allo-HSCT (8 weeks to days before) Weekly during Hospitalization D + 90 D + 180 D + 360 D + 450	**Before Conditioning** There was no difference in oral microbial diversity at baseline between individuals who did and did not develop oral cGvHD (PERMANOVA, *p* > 0.001; Mann–Whitney U test, *p* > 0.05). **All Samples** There was no significant association between oral cGvHD and microbial diversity at any time (*p* = 0.451). The trajectory of microbial diversity over time differed between patients with and without oral cGvHD. In patients without oral cGvHD, microbial diversity significantly decreased at weeks 1, 2, and 3 (linear mixed model, *p* = 0.037; *p* = 0.002; *p* < 0.001). In contrast, patients with oral cGvHD showed a slight but significant increase in diversity at 12 and 18 months (linear mixed model, *p* = 0.048; *p* = 0.017).
	Kambara 2025 [58] 31	Before Allo-HSCT After Allo-HSCT	**Before Allo-HSCT** Yes cGvHD vs. No cGvHD ↓ Shannon index in patients with cGvHD (*p* = 0.001). **After Allo-HSCT** Yes cGvHD vs. No cGvHD ↓ Shannon index in patients with cGvHD (*p* = 0.0001). **All Samples** Yes cGvHD vs. No cGvHD ↓ Shannon index ratio in patients with cGvHD (*p* = 0.0001).
	Brehm 2025 [48] 43	At Admission	**At Admission** Oral β-diversity was not significantly affected by the presence of active cGVHD at the time of enrollment (*p* = 0.095).
**Relapse** [2,56]	de Molla 2020 [56] 30	Before Conditioning	**↓ Relapse** When compared to patients with low diversity, those with high diversity had lower risk of relapse at 3 years (68% vs. 33%; *p* = 0.04). This association lost significance after adjusting for DRI (HR = 0.30; 95% CI 0.08–1.09; *p* = 0.07).
	De Molla 2021 [2] 30	Before Conditioning At Aplasia At Engraftment	**Before Conditioning** **↑ Relapse** When compared to patients with higher diversity, those with lower diversity had a higher risk of relapse at 3 years (68% versus 33%, respectively; HR 0.27, 95% CI 0.07–0.97, *p* = 0.04). This association lost significance after adjusting for DRI (HR 0.30, 95% CI 0.08–1.09, *p* = 0.07). **At Aplasia** No significant differences in relapses were observed between patients with high and low diversity (HR 1.30; 95% CI 0.43–3.90; *p* = 0.64). **At Engraftment** No significant differences in relapse were observed between patients with high and low diversity (HR 0.73; 95% CI 0.21–2.53; *p* = 0.62).
**Other** [2,48]	De Molla 2021 [2] 30	Before Conditioning At Aplasia At Engraftment	**PFS** **Low vs. High Diversity** No significant differences in PFS were observed between patients with high and low diversity (6% vs. 32%; HR 0.75, 95% CI 0.28–2.00, *p* = 0.57).
			**NRM** **Low vs. High Diversity** No significant differences in NRM were observed between patients with high and low diversity (18% vs. 0%, HR 4.12, 95% CI 0.86–19.32, *p* = 0.07).
			**Febrile Neutropenia** **Low vs. High Diversity** No association was observed between oral microbial diversity and the occurrence of febrile neutropenia (*p* = 0.22). **At Aplasia** No association was observed between oral microbial diversity and the occurrence of febrile neutropenia (*p* = 0.48). **At Engraftment** No association was observed between oral microbial diversity and the occurrence of febrile neutropenia (*p* = 0.48).
	Brehm 2025 [48] 43	Not Clear	**Bronchiolitis Obliterans** Patients with bronchiolitis obliterans showed a significant reduction in oral microbiota diversity when compared with patients with transient pulmonary impairment, pre-bronchiolitis obliterans, and controls (*p* = 0.0057).

aGvHD = acute Graft-versus-Host Disease; Allo-HSCT = allogeneic hematopoietic stem cell transplantation; CI = Confidence Interval; cGvHD = chronic Graft-versus-Host Disease; D = day; DRI = Disease risk index; HR = Hazard Ratio; N = number of patients included in the analysis; NR = Not Reported; NRM = Non-Relapse Mortality; PFS = Progression-Free Survival; ↑ = Increased; ↓ = Decreased.

**Table 2:** Clinical Implications of Oral Microbiota Composition Over the Allo-HSCT.

Outcome	Author, Year N	Sample Timing	Findings
**Oral Mucositis** [9,11,12,13,15,32,41,42,43,44,47,54,55]	Bartha 2025 [9] 25	Before Allo-HSCT After Allo-HSCT	**Before Allo-HSCT** No significant differences in RSV abundances were observed between patients with and without ulcerative mucositis. **After Allo-HSCT** Patients with ulcerative mucositis exhibited significant differences in the abundance of the following bacterial taxa: ↑ *Mycoplasma salivarium* (*p* < 0.05). ↓ *Rothia mucilaginosa* (*p* < 0.001). ↓ *Prevotella histicola* (*p* < 0.05). ↓ *Oribacterium sinus* (*p* < 0.05). ↓ *Leptotrichia* sp. (*p* < 0.001). ↓ *Haemopilus* sp. (*p* < 0.05). ↓ *Gemella* sp. (*p* < 0.001). ↓ *Fusobacterium periodonticum* (*p* < 0.05). ↓ *Alloprevotella* sp. (*p* < 0.05).
	Bruno 2022 [11] 30	Before Conditioning Oral Mucositis Onset Oral Mucositis Healing	**Before Conditioning vs. Oral Mucositis Onset** ↑ *Lactobacillus* (*p* < 0.001). ↑ *Mycoplasma* (*p* < 0.001). ↑ *Parvimonas* (*p* < 0.05). ↓ *Catonella* (*p* < 0.001). **Before Conditioning vs. Oral Mucositis Healing** ↑ *Lactobacillus* (*p* < 0.001). ↑ *Enterococcus* (*p* < 0.001). ↑ Unclassified *Bifidobacteriaceae* (*p* < 0.001). ↑ *Treponema 2* (*p* < 0.001). ↑ *Lactococcus* (*p* < 0.05). ↑ *Staphylococcus* (*p* < 0.001). ↓ *Porphyromonas* (*p* < 0.001). ↓ *Haemophilus* (*p* < 0.001). ↓ *Lachnoaerobaculum (p* < 0.001). ↓ *Neisseria (p* < 0.001). ↓ *Bergeyella (p* < 0.001). **Oral Mucositis Onset vs. Oral Mucositis Healing** ↑ *Delftia* (*p* < 0.05). ↓ *Porphyromonas (p* < 0.001). **Before Conditioning** Patients who developed oral mucositis exhibited *β*-diversity indices similar to those of patients who did not develop the condition (F = 0.95; *p* = 0.49). Additionally, Cox regression analysis revealed no associations between the relative abundance of any genus in pre-conditioning samples and the subsequent risk of developing oral mucositis. An SVM model based on pre-conditioning microbiota profiles identified a signature of eight genera that predicted oral mucositis onset with an accuracy of 96.6%. These genera were: (1) *Bergeyella*, (2) *Fusobacterium*, (3) *Gemella*, (4) *Haemophilus*, (5) *Prevotella*, (6) *Prevotella 6*, (7) *Selenomonas 3*, and (8) *Streptococcus.* When analyzing the entire cohort, no genera in pre-conditioning samples were associated with oral mucositis severity. However, when restricting the analysis to patients who developed severe oral mucositis, the relative abundance of *Porphyromonas* was significantly correlated with severity (*p* = 0.028). **Oral Mucositis Onset** Patients with higher *Lactobacillus* relative abundance (above the median) exhibited a significantly shorter healing time compared to those with lower abundance (6 vs. 10 days; *p* = 0.032).
	Chukhlovin 2019 [54] 202	Before Allo-HSCT D + 30 D + 60 D + 90 D + 120	**All Samples** There were no significant associations between the most common bacterial species and oral mucositis: *Streptococcus viridans* (*p* = 0.81). *Staphylococcus epidermidis* (*p* = 0.67). *Neisseria* spp. (*p* = 0.75). *Corynebacterium* spp. (*p* = 0.10). *Klebsiella pneumoniae* (*p* = 0.67).
	Faraci 2024 [12] 17	Before Allo-HSCT At Engraftment D + 30 D + 100	**At Engraftment** The following bacteria had significantly higher abundance in samples at engraftment from patients that developed oral mucositis: ↑ *Coriobacteriaceae* (FDR = 0.0320). ↑ *Atopobium* (FDR = 0.0205). ↑ *Coriobacteriia* (FDR = 0.0017). ↑ Clostridiales XI incertae sedis (FDR = 0.0388). ↑ *Fusobacteria* (FDR = 0.0195). ↑ *Tenericutes* (FDR = 0.0064). ↑ *Mollicutes* (FDR = 0.0177). ↑ *Mycoplasmatales* (FDR = 0.0119). ↑ *Mycoplasmataceae* (FDR = 0.0334). ↑ *Mycoplasma* (FDR = 0.0481). **D + 30** The following bacteria had significantly higher abundance in samples from D + 30 from patients that developed oral mucositis: ↑ *Prevotellaceae* (FDR = 0.0188). ↑ *Prevotella* (FDR = 0.0491). ↑ *Catonella* (FDR = 0.0065). ↑ *Peptostreptococcaceae* (FDR = 0.0300). ↑ *Peptostreptococcus* (FDR = 0.0267). ↑ *Selenomonas* (FDR = 0.000000018). ↑ *Fusobacterium periodonticum* (FDR = 0.0050). ↑ *Eikenella* (FDR = 0.00029). ↑ *Campylobacter curvus* (FDR = 0.0000000017). ↑ *Serratia* (FDR = 0.00023). **All Samples** The following bacteria had significantly higher abundance in samples from patients that never developed oral mucositis: ↑ *Micrococcaceae* (FDR = 0.0069). ↑ *Rothia aeria* (FDR = 0.0133). ↑ *Rothia denticariosa* (FDR = 0.0133). ↑ *Actinomyces graevenitzii* (FDR = 0.0228). ↑ *Actinomyces* sp. (FDR = 0.0228). ↑ *Corynebacteriaceae* (FDR = 0.0048). ↑ *Corynebacterium* (FDR = 0.0008). ↑ *Campnocytophaga gingivalis* (FDR = 0.0484). ↑ *Gemella sanguinis* (FDR = 0.0444). ↑ *Streptococcus* (FDR = 0.0087). ↑ *Streptococcus cristatus* (FDR = 0.0448). ↑ *Streptococcus gordonii* (FDR = 0.0000000000034). ↑ *Streptococcus lactarius* (FDR = 0.0228). ↑ *Streptococcus mutans* (FDR = 0.0076). ↑ *Roseburia intestinalis* (FDR = 0.0377).
	Laheij 2012 [32] 49	Before Conditioning Twice Weekly until Hospital Discharge	**All Samples** **↑ Oral Ulcerations (non-keratinized mucosa)** The presence of *P. gingivalis* (*p* = 0.007) and *C. kefyr* (*p* = 0.029) was significantly associated with oral ulcerations (non-keratinized mucosa) in the multivariable model. Similarly, the load of *P. gingivalis* (*p* = 0.004) and *C. kefyr* (*p* = 0.013) was significantly associated with oral ulcerations (non-keratinized mucosa). In another multivariate analysis, the proportions of P. *gingivalis* (*p* = 0.001), *P. micra* (*p* = 0.001), *T. denticola* (*p* = 0.000), *F. nucleatum* (*p* = 0.015), and *C. glabrata* (*p* = 0.000) relative to the total bacterial load were significantly associated with oral ulcerations (non-keratinized mucosa). **↑ Oral Ulcerations (keratinized mucosa)** The presence of *P. gingivalis* (*p* = 0.005), P. *micra* (*p* = 0.043), and *C. kefyr* (*p* = 0.005) was significantly associated with oral ulcerations (keratinized mucosa) in the multivariable model. Similarly, the load of *P. gingivalis* (*p* = 0.034) and *C. kefyr* (*p* = 0.028) was significantly associated with oral ulcerations (keratinized mucosa).
	Laheij 2022 [15] 50	Before Allo-HSCT (8 weeks to days before) Weekly during Hospitalization D + 90 D + 180 D + 360 D + 450	**D + 21** Patients with oral mucositis exhibited reduced relative abundances of *Streptococcus* and *Neisseria*, along with increased abundances of *Prevotella*, *Alloprevotella*, *Mycobacterium*, *Staphylococcus*, and *Enterococcus* (details NR). **D + 90** Patients with oral mucositis exhibited increased relative abundance of *Neisseria* and *Veillonella*, along with decreased abundance of *Prevotella*. **All Samples after Allo-HSCT** Patients with oral mucositis exhibited higher relative abundances of *Actinobaculum*, *Lactobacillus*, and *Staphylococcus* and lower abundances of *Saccharibacteria* and *Scardovia*.
	Shouval 2019 [41] 184	D − 7 to D − 1 D0 to D + 6 D + 7 to D + 13 D + 14 to D + 20 D + 21 to D + 34	**Prior to Allo-HSCT** Patients who developed oral mucositis demonstrated increased relative abundances of *Rothia*, *Kingella*, and *Atopobium*. **D** − **7 to D + 13** Patients who developed grade 3–4 oral mucositis exhibited a more pronounced increase in *β*-diversity (*p* < 0.005). **D + 7 to D + 13** Patients with severe mucositis were more likely to harbor *Methylobacterium*, whereas those with mild mucositis exhibited higher relative abundances of *Treponema* and the genus TG5.
	Shouval 2020 [42] 184	NR	**Prior to Allo-HSCT** Pre-transplantation identification of *Kingella* and *Atopobium* was predictive of subsequent development of oral mucositis (details NR). **All Samples** Severe oral mucositis was associated with an increase in *β*-diversity over time (*p* < 0.0054). **All Samples** Patients with grade 3–4 oral mucositis exhibited increased relative abundances of *Mycoplasma*, *Methylobacterium*, *Campylobacter*, and *Staphylococcus*.
	Shouval 2020 [43] 184	Weekly from D − 7 to D + 34	**Prior to Allo-HSCT** Pre-transplantation identification of *Kingella*, *Rothia*, and *Atopobium* was predictive of subsequent development of oral mucositis (details NR). **D + 7 to D + 13** Patients with grade 3–4 oral mucositis showed a higher abundance of *Methylobacterium*, whereas those with mild mucositis had higher levels of *Treponema* and genus TG5.
	Shouval 2020 [44] 184	D − 7 to D − 1 D0 to D + 6 D + 7 to D + 13 D + 14 to D + 20 D + 21 to D + 27 D + 28 to D + 34	**D** − **7 to D** − **1** **Grade 1–2 vs. Grade 3–4 Oral Mucositis** *Kingella* (*p* = 0.0009) and *Atopobium* (*p* < 0.05) were predictive of severe oral mucositis in two independent models (XGBoost and linear discriminant analysis effect size). **D** − **7 to D + 13** Among patients with grade 0–1 oral mucositis, significant changes were observed in several taxa, including increased abundance of *Kingella* and *Eikenella* and decreased abundance of *Oribacterium*, *Moryella*, *Catonella*, *Butyrivibrio*, unclassified members of the *Lachnospiraceae* and *Clostridiales*, *Lactobacillus*, *Capnocytophaga*, and *Prevotella*. Among patients with grade 3–4 oral mucositis, significant changes were observed in several taxa, including increased abundance of *Mycoplasma*, *Campylobacter*, *Methylobacterium*, an unclassified member of the *Gemellaceae* family, and *Staphylococcus*. Patients also demonstrated decreased abundance of multiple taxa, including TG5, *Haemophilus*, *Neisseria*, *Kingella*, *Leptotrichia*, *Fusobacterium*, *Bulleidia*, *Parvimonas*, *Mogibacterium*, an unclassified member of the *Mogibacteriaceae*, *Selenomonas*, *Schwartzia*, *Megasphaera*, an unclassified member of the *Veillonellaceae*, *Oribacterium*, *Moryella*, *Catonella*, unclassified members of the *Lachnospiraceae* and *Clostridiales* families, *Granulicatella*, *Capnocytophaga*, *Paraprevotellaceae Prevotella*, *Prevotellaceae Prevotella*, *Atopobium*, *Rothia*, *Corynebacterium*, and *Actinomyces*. **D + 7 to D + 13** **Grade 1–2 vs. Grade 3–4 Oral Mucositis** Patients with grade 3–4 oral mucositis showed a higher abundance of *Methylobacterium*, whereas those with mild mucositis had higher levels of *Treponema* and genus TG5.
	Takahashi 2020 [47] 19	Before Allo-HSCT Mucositis Peak After Engraftment	**Before Allo-HSCT to Mucositis Peak** **Oral Mucositis vs. Controls** The mean distance was significantly longer in the moderate and severe mucositis groups than in the control group (moderate vs. control: 424.5 [61.3] vs. 169.4 [47.1], *p* = 0.031; severe vs. control: 510.2 [64.1] vs. 169.4 [47.1], *p* = 0.016). **Mucositis Peak to Post-engraftment** **Oral Mucositis vs. Controls** The mean distance was significantly longer in the moderate and severe mucositis groups than in the control group (moderate vs. control: 659.4 [108.0] vs. 101.2 [22.3], *p* = 0.0075; severe vs. control: 330.4 [57.1] vs. 101.2 [22.3], *p* = 0.036).
	Faraci 2023 [55] 17	Before Allo-HSCT At Engraftment D + 30 D + 100	**All Samples** The following taxa were significantly associated with oral mucositis: *Atopobium*. *Campylobacter*. *Fusobacterium periodonticum*. *Prevotella species*. *Prevotellaceae*. *Fusobacteriaceae*.
	Gem 2024 [50] 56	Baseline D + 7 D + 14 D + 21 D + 28 D + 84	**Baseline** **Oral Saliva** In a LASSO model, the presence of *Oribacterium asaccharolyticum* in baseline samples was associated with greater oral mucositis severity at D + 14 (*p* = 0.01). In a multivariable linear regression model including sex, conditioning intensity, baseline salivary *Oribacterium asaccharolyticum* presence/absence, and day +14 salivary flow rate as potential predictors of the day +14 total mucositis score, *Oribacterium asaccharolyticum* presence remained the only significant variable (*p* = 0.036). **Baseline** **Supragingival Plaque** In a LASSO model, the presence of Rothia species in baseline samples was associated with greater oral mucositis severity at D + 21 (specific details NR).
	Oku 2020 [13] 45	D0	**D0** The presence of the following bacterial taxa was not associated with oral mucositis: *Ralstonia pickettii* (*p* values NR). *Staphylococcus haemolyticus* (*p* value NR). *Streptococcus thermophilus* (*p* value NR).
**Overall Survival** [2,13,37]	De Molla 2021 [2] 30	Before Conditioning At Aplasia At Engraftment	**Before Conditioning and at Engraftment** ↑ *Enterococcus*, *Lactobacillus*, *Mycoplasma*, and *Staphylococcus*: changes in the relative abundance of these genera were not associated with overall survival (*p* = 0.31, *p* = 0.80, *p* = 0.43, and *p* = 0.34). **Before Conditioning** The presence of *Solobacterium* was not significantly associated with overall survival (55% vs. 28%, HR 0.99, 95% CI 0.32–3.08, *p* = 0.99).
	Diamond 2023 [37] 95	Before Conditioning D + 90	**Before Conditioning** *Streptococcus* was significantly enhanced in patients who died within 1 year. **D + 90** *Enterococcus* was significantly enhanced in patients who died within one year (*p* value NR). *Streptococcus and Actinomyces* were significantly enhanced in patients who died within 5 years (*p* values NR).
	Oku 2020 [13] 45	D0	**D0** The presence of the following bacterial taxa was associated with significantly lower overall survival: *Staphylococcus haemolyticus* (*p* = 0.009). *Ralstonia pickettii* (*p* = 0.02). *Staphylococcus haemolytics* and/or *Ralstonia pickettii* (*p* = 0.003). The estimated one-year overall survival was 37.8% in patients positive for *Staphylococcus haemolyticus* and/or *Ralstonia pickettii*, versus 78.2% in those who tested negative for these taxa. The association between the presence of *Staphylococcus haemolyticus* and/or *Ralstonia pickettii* and overall survival remained significant in the multivariate model (adjusted HR = 2.5; 95% CI, 1.0–6.4; *p* = 0.04).
**aGvHD** [2,10,13,31,54]	Chukhlovin 2019 [54] 202	Before Allo-HSCT D + 30 D + 60 D + 90 D + 120	**All Samples** There were no significant associations between the most common bacterial species and aGvHD: *Streptococcus viridans* (*p* = 0.37). *Staphylococcus epidermidis* (*p* = 0.88). *Neisseria* spp. (*p* = 0.34). *Corynebacterium* spp. (*p* = 0.80). *Klebsiella pneumoniae* (*p* = 0.36).
	Oku 2020 [13] 45	D0	**D0** The presence of the following bacterial taxa was not associated with aGvHD: *Ralstonia pickettii* (*p* values NR). *Staphylococcus haemolyticus* (*p* value NR). *Streptococcus thermophilus* (*p* value NR).
	De Molla 2021 [2] 30	Before Conditioning At Aplasia At Engraftment	**Before Conditioning and at Engraftment** ↑ *Enterococcus*, *Lactobacillus*, *Mycoplasma*, and *Staphylococcus*: changes in the relative abundance of these genera were not associated with aGvHD (*p* = 0.54, *p* = 0.14, *p* = 0.96, and *p* = 0.26) and severe aGvHD (*p* = 0.52, *p* = 1.00, *p* = 0.42, and *p* = 0.58). **Before Conditioning** The presence of *Solobacterium* was not significantly associated with aGvHD (64% vs. 44%, HR 1.84, 95% CI 0.68–4.95, *p* = 0.23).
	Heidrich 2021 [10] 30	Before Conditioning At Aplasia At Engraftment	**Before Conditioning** Increased *Streptococcus* (HR = 2.89, 95% CI 1.07–7.79, *p* = 0.036) and *Corynebacterium* (HR = 2.74, 95% CI 1.05–7.15, *p* = 0.04) abundances were associated with higher aGvHD risk. After adjustment, only *Streptococcus* remained significant (HR = 3.17, 95% CI 1.12–9.01, *p* = 0.03). Conversely, *Veillonella* abundance was linked to a reduced aGvHD risk (HR = 0.24, 95% CI 0.08–0.70, *p* = 0.006), persisting after adjustment (HR = 0.21, 95% CI 0.07–0.65, *p* = 0.006). *Veillonella*/*Streptococcus* ratio > 1 before conditioning was associated with a lower risk of aGvHD (HR = 0.23, 95% CI 0.08–0.62, *p* = 0.004). This remained significant after adjusting for graft source and conditioning regiment intensity (HR = 0.22, 95% CI 0.08–0.64, *p* = 0.005). **At Aplasia + At Engraftment** No similar associations between the relative abundance of the three aforementioned genera and the ratio were observed in samples collected during aplasia or engraftment were identified.
	Ingham 2021 [31] 29	At Pre-examination D0 D + 7 D + 14 D + 21 D + 30 D + 90 D + 180 D + 360	**Before Allo-HSCT** **Grade II–IV vs. Grade 0–I aGvHD** ↑ *Aerococcacea* (*p* value NR). ↑ *Prevotellaceae* (*p* value NR). ↓ *Neisseriacea* (*p* value NR). A machine learning approach predicted aGvHD severity based on the abundance of three significant oral ASVs: ASV 568 *Actinomyces* sp. (*p* < 0.001). ASV 226 *Prevotella melaningonecia* (*p* <0.001). ASV 500 *Pseudopropionibacterium propionicum* (*p* <0.001).
**cGvHD** [2,15,40,57,58]	De Molla 2021 [2] 30	Before Conditioning At Aplasia At Engraftment	**Before Conditioning and at Engraftment** ↑ *Enterococcus* relative abundance was significantly associated with higher incidence of cGvHD (*p* = 0.03). ↑ *Lactobacillus*, *Mycoplasma*, and *Staphylococcus*: changes in the relative abundance of these genera were not associated with cGvHD (*p* = 0.63, *p* = 0.46, *p* = 0.36). **Before Conditioning** The presence of *Solobacterium* was not significantly associated with cGvHD (27% vs. 13%, HR 2.41, 95% CI 0.43–13.4, *p* = 0.31).
	Ganesan 2021 [57] 10	Before Allo-HSCT D + 60 D + 180 D + 360 cGvHD Onset	**Other Timepoints vs. cGvHD Onset** The following genera were significantly increased only in patients at the onset of cGvHD: ↑ *Sphingomonas* (specific data NR). ↑ *Lautropia* (specific data NR). **Yes cGvHD vs. No cGvHD** The following fungal species were significantly elevated in patients with cGvHD: ↑ *Candida* (specific data NR). ↑ *Trichoderma* (specific data NR). ↑ *Thielavia* (specific data NR). ↑ *Myceliphthora* (specific data NR).
	Kambara 2025 [58] 31	Before Allo-HSCT After Allo-HSCT	**Before Allo-HSCT** **Yes cGvHD vs. No cGvHD** PCoA showed no significant differences in microbiota composition when patients were stratified by cGVHD (*p* = 0.76). **After Allo-HSCT** **Yes cGvHD vs. No cGvHD** PCoA showed no significant differences in microbiota composition when patients were stratified by cGVHD (*p* = 0.83). The following bacterial families were more prevalent in patients with cGVHD: ↑ Staphylococcaceae (specific NR). ↑ Enterobacteriaceae (specific NR). The following bacterial families were more prevalent in patients without cGVHD: ↑ *Prevotellaceae* (specific data NR). ↑ *Veillonellaceae* (specific data NR). ↑ *Lactobacillaceae* (specific data NR).
	Rashid 2024 [40] 80	Before Conditioning cGvHD Onset First cGvHD Follow-up Visit D + 360	**Before Conditioning** Before conditioning, there were no significant differences in baseline microbiota composition between patients who subsequently developed cGVHD and those who did not. **D + 360** At 1-year follow-up there were no significant differences in microbiota composition between patients who subsequently developed cGVHD and those who did not (*p* = 0.43). **All Samples** There was a significant difference in microbial composition between samples collected from patients with cGVHD and those without (*p* = 0.002). **Before Conditioning and Onset of cGvHD** At the time of cGvHD onset, there was a significant increase in the relative abundance of *Veillonella parvula* (Padj = 0.06) and *Streptococcus salivarius* (Padj = 0.06).
	Laheij 2022 [15] 50	Before Allo-HSCT (8 weeks to days before) Weekly during Hospitalization D + 90 D + 180 D + 360 D + 450	**Before Allo-HSCT and at Time of cGvHD Development** No significant differences in microbial composition were observed between samples from before allo-HSCT and samples taken at the time of cGvHD development (restricted PERMANOVA, F = 1.0316, *p* = 0.0341). **All Samples** **Yes cGvHD vs. No cGvHD** There was a significant effect of time on microbiome composition in both groups (restricted PERMANOVA, F = 1.1191, *p* = 0.0001; F = 2.1962, *p* = 0.0001).
**Relapse** [2,56]	de Molla 2020 [56] 30	Before Conditioning	**↑ *Solobacterium*** Presence of *Solobacterium* was associated with lower relapse risk (9% vs. 56%; *p* = 0.04). This remained significant after adjusting for DRI (HR = 0.20; 95% CI 0.006–0.67; *p* = 0.01)
	De Molla 2021 [2] 30	Before Conditioning At Aplasia At Engraftment	**Before Conditioning and at Engraftment** ↑ *Enterococcus*, *Lactobacillus*, *Mycoplasma*, and *Staphylococcus*: changes in the relative abundance of these genera were not associated with relapse risk (*p* = 0.78, *p* = 0.74, *p* = 0.19, and *p* = 0.09). **Before Conditioning** ↑ *Solobacterium*: this was the only genus significantly associated with lower relapse risk (9% vs. 56%, respectively; HR 0.23, 95% CI 0.05–0.94, *p* = 0.04). This association remained significant after adjusting for DRI (HR 0.20, 95% CI 0.06–0.67, *p* = 0.01). However, this association lost significance after adjusting for multiple comparisons using the Bonferroni correction (*p* = 0.72). The following bacteria were not significantly associated with relapse risk: Uncultured *Saccharimonadaceae* (*p* = 0.62). *Lachnoanaerobaculum* (*p* = 0.26). *Fusobacterium* (*p* = 0.40). *Catonella* (*p* = 0.40). *Atopoblum* (*p* = 0.43). *Haemophilus* (*p* = 0.49). *Porphyromonas* (*p* = 0.59). *Bergeyella* (*p* = 0.78). *Selenomonas* 3 (*p* = 0.82). *Ruminococcaceae* UCG 014 (*p* = 0.84). *Oribacterium* (*p* = 0.89). *Stomatobaculum* (*p* = 0.93). *Alloprevotella* (*p* = 0.83). *Megasphaera* (*p* = 0.79). *Prevotella 6* (*p* = 0.80). *Neisseria* (*p* = 0.43). *Capnocytophaga* (*p* = 0.45).
**Other** [2,13,35,39,54]	De Molla 2021 [2] 30	Before Conditioning At Aplasia At Engraftment	**PFS** **Before Conditioning and at Engraftment** ↑ *Enterococcus*, *Lactobacillus*, *Mycoplasma*, and *Staphylococcus*: changes in the relative abundance of these genera were not associated with PFS (*p* = 0.53, *p* = 0.61, *p* = 0.36, and *p* = 0.06). **Before Conditioning** The presence of *Solobacterium* was not significantly associated with PFS (55% vs. 37%, HR 0.83, 95% CI 0.31–0.83, *p* = 0.71). **NRM** **Before Conditioning and at Engraftment** ↑ *Enterococcus*, *Lactobacillus*, *Mycoplasma*, and *Staphylococcus*: changes in the relative abundance of these genera were not associated with NMR (*p* = 0.19, *p* = 0.10, *p* = 0.12, and *p* = 0.67).
	Oku 2020 [13] 45	D0	**Bacteremia** **D0** The presence of the following bacterial taxa was not associated with bacteremia: *Ralstonia pickettii* (*p* values NR). *Staphylococcus haemolyticus* (*p* value NR). *Streptococcus thermophilus* (*p* value NR).
	Chukhlovin 2019 [54] 202	Before Allo-HSCT D + 30 D + 60 D + 90 D + 120	**Infections** **All Samples** There were no significant associations between four of the most common bacterial species and infections: *Streptococcus viridans* (*p* = 0.146). *Staphylococcus epidermidis* (*p* = 0.304). *Neisseria* spp. (*p* = 0.23). *Corynebacterium* spp. (*p* = 0.25). There was a significant association between positive *Klebsiella pneumoniae* cultures and the occurrence of infections (10.2% vs. 4.9%; *p* = 0.02). **Fever of Unknown Origin** **All Samples** There were no significant associations between the most common bacterial species and fever of unknown origin: *Streptococcus viridans* (*p* = 0.65). *Staphylococcus epidermidis* (*p* = 0.06). *Neisseria* spp. (*p* = 0.62). *Corynebacterium* spp. (*p* = 0.15). *Klebsiella pneumoniae* (*p* = 0.48).
	Ohbayashi 2021 [39] 96	At the Time of Fever D + 30	**Infections** BSIs caused by oral microorganisms occurred in 12 (46.2%) cases. These included 5 cases *of Staphylococcus epidermidis*, 4 cases of *Stenotrophomonas maltophilia*, 2 cases of *Enterococcus faecium*, and 1 case of methicillin-resistant *Staphylococcus aureus* (MRSA).
	Ames 2012 [35] 11	Before Allo-HSCT Neutrophil Nadir At Engraftment Respiratory Infection ICU Admission	**Respiratory Manifestations** **Yes Respiratory Manifestations vs. No Respiratory Manifestations** There were several differences in the relative number of positive probes for certain bacterial taxa (see below). Additionally, principal component analysis demonstrated no overlap between the groups, suggesting a clear separation in microbiota composition in both groups. ↓ *Streptococcus Cluster III* (details NR). ↓ *Streptococcus oralis* (details NR). ↓ *Granulicatellaadiacens elegans II* (details NR). ↓ *Streptococcus infantis* (details NR). ↓ *Granulicatellaadiacensl* (details NR). ↓ *Rothia dentocariosa* or *R. mucilaginosa* (details NR). ↑ *Veillonella Cluster II* (details NR). ↑ *Veillonella Cluster IV* (details NR). ↑ *Streptococcus parasanguinis II* (details NR). ↑ *Veillonella atypical II* (details NR). ↑ *Gemellahaemolysans sanguisl* (details NR). ↑ *Campylobacter rectus* or *C. concisus* (details NR). ↑ *Streptococcus parasanguinis BE024I* (details NR). ↑ *Preptostreptococcus microsl* (details NR). ↑ *Veillonella parvulal* (details NR). ↑ *Streptococcus anginosis or Streptoccocus gordoniil* (details NR).

aGvHD = acute Graft-versus-Host Disease; Allo-HSCT = allogeneic hematopoietic stem cell transplantation; ASV = Amplicon Sequence Variant; CI = Confidence interval; cGvHD = chronic Graft-versus-Host Disease; D = day; DRI = Disease risk index; FDR = False Discovery Rate; HR = Hazard Ratio; ICU = Intensive Care Unit; N = number of patients included in the analysis; NR = Not Reported; PFS = Progression-Free Survival; SMV = Support Vector Machine; ↑ = Increased; ↓ = Decreased.

**Table 3:** Clinical Implications of Oral Microbiota Domination Over the Allo-HSCT.

Outcome	Author, Year N	Sample Timing	Findings
**Overall Survival** [2,56]	de Molla 2020 [56] 30	Before Conditioning	**↓ Overall Survival** Dominance by any genus was associated with inferior overall survival (38% vs. 81%; *p* = 0.02). This association lost significance after adjusting for DRI (HR = 4.12; 95% CI 0.089–19.13; *p* = 0.07).
	De Molla 2021 [2] 30	Before Conditioning	**↓ Overall Survival** Patients with dominance by any genus exhibited inferior overall survival compared with those without dominance (38% vs. 81%, HR 4.73, 95% CI 1.59–14.08, *p* = 0.02). This association lost significance after adjusting for DRI (HR 4.12, 95% CI 0.89–19.13, *p* = 0.07).
**aGvHD** [2,56]	De Molla 2021 [2] 30	Before Conditioning	**Before Conditioning** No significant differences in aGvHD were observed between patients with and without dominance by any genus (43% vs. 63%; HR 0.50, 95% CI 0.18–1.37, *p* = 0.18).
	Heidrich 2021 [10] 30	Before Conditioning At Aplasia At Engraftment	**All Samples** Patients with oral domination by any genus did not show a significant association with aGvHD risk (HR = 2.29; 95% CI 0.63–2.36; *p* = 0.21). ***Enterococcus* Domination** *Enterococcus* domination was significantly associated with aGvHD (HR = 4.07, 95% CI 1.82–9.14, *p* = 0.0007). This remained significant after adjusting for graft source and conditioning regimen intensity (HR = 4.90, 95% CI 1.66–14.50, *p* = 0.004). *Enterococcus* domination was significantly associated with severe aGvHD (HR = 6.82, 95% CI 1.48–31.41, *p* = 0.014).
**cGvHD** [2]	De Molla 2021 [2] 30	Before Conditioning	**Before Conditioning** No significant differences in cGvHD were observed between patients with and without dominance by any genus (19% vs. 18%; HR 1.07, 95% CI 0.19–5.93, *p* = 0.94).
**Relapse** [2,56]	de Molla 2020 [56] 30	Before Conditioning	**↑ Relapse** Dominance by any genus was associated with increased relapse risk at 3 years when compared to patients without any dominance (63% vs. 36%; *p* = 0.003). This remained significant after adjusting for DRI (HR = 4.19; 95% CI 1.25–14.1; *p* = 0.02).
	De Molla 2021 [2] 30	Before Conditioning	**↑ Relapse** Patients with dominance by any genus exhibited increased relapse risk compared with those without dominance (63% vs. 36%; HR 4.59, 95% CI 1.11–19; *p* = 0.003). This remained significant after adjusting for DRI (HR 4.19, 95% CI 1.25–14.1, *p* = 0.02). ***Streptococcus* Dominance** No significant differences in relapse rates were observed between patients with and without *Streptococcus* dominance (56% vs. 39%; HR = 1.64, 95% CI 0.52–5.14, *p* = 0.40). **Facultative Anaerobic Genera Dominance** No significant differences in relapse rates were observed between patients with and without facultative anaerobic genera dominance (6% vs. 39%; HR 2.05, 95% CI 0.67–6.27, *p* = 0.21).
**Other** [2,36,56]	Heidrich 2023 [36] 31	Before Conditioning At Aplasia At Engraftment D + 30 D + 75	**↑ Respiratory Infections** Among the three patients that developed respiratory infections over the allo-HSCT, two had a domination event of the same genus identified in the microbiological exam of their respiratory tract samples 1–2 weeks before the infection. **Bacteremia** There was no significant association between oral microbiota domination and bacteremia. GCF: OR 3.17; *p* = 0.156. OM: OR 2.25; *p* = 0.299. SR: OR 0.92; *p* = 1.
	de Molla 2020 [56] 30	Prior to Conditioning	**↓ PFS** Dominance by any genus was associated with inferior PFS (19% vs. 55%; *p* = 0.01). This remained significant after adjusting for DRI (HR = 4.14; 95% CI 1.15–14.89; *p* = 0.03).
	De Molla 2021 [2] 30	Before Conditioning	**↓ PFS** Patients with dominance by any genus exhibited inferior PFS compared with those without dominance (19% vs. 55%; HR 4.75, 95% CI 1.78–12.7, *p* = 0.01). This remained significant after adjusting for DRI (HR = 4.14; 95% CI 1.15–14.89; *p* = 0.03). **NMR** No significant differences in NMR were observed between patients with and without dominance by any genus (20% vs. 9%; HR 2.35, 95% CI 0.27–20.60, *p* = 0.44).

aGvHD = acute Graft-versus-Host Disease; Allo-HSCT = allogeneic hematopoietic stem cell transplantation; CI = Confidence interval; cGvHD = chronic Graft-versus-Host Disease; D = day; DRI = Disease risk index; GCF = gingival crevicular fluid; HR = Hazard Ratio; N = number of patients included in the analysis; NRM = Non-Relapse Mortality; OM = oral mucosa; OR = Odds ratio; PFS = Progression-Free Survival; SB = supragingival biofilm; ↑ = Increased; ↓ = Decreased.

## Data Availability

No new data were created or analyzed in this study.

## Supplementary Materials

The following supporting information can be downloaded at: https://www.mdpi.com/article/10.3390/microorganisms14020308/s1, Table S1: Additional studies identified from other oral microbiota reviews; Table S2A: Methodological Quality Assessment of Included Cohort Studies. Table S2B: Methodological Quality Assessment of Included Cross-Section Studies. Table S3: Patient Demographics and Oral Microbiota Methodologies; Table S4: Factors Impacting Oral Microbiota Over the Allo-HSCT; Table S5: Studies Evaluating the Dynamics of Oral Microbiota Over the Allo-HSCT [65,66,67,68,69,70,71].

## Abbreviations

aGvHD	Acute graft-versus-host disease
Allo-HSCT	Allogeneic hematopoietic stem cell transplantation
ANCOM	Analysis of composition of microbiomes
ASV	Amplicon sequence variant
BSI	Bloodstream infection
cGvHD	Chronic graft-versus-host disease
CI	Confidence interval
D	Day
DMF-T	Decayed, Missing, Filled Teeth Score
DOT	Days of therapy
DRI	Disease risk index
FDR	False discovery rate
GCF	Gingival crevicular fluid
GvHD	Graft-versus-host disease
HCT-CI	Hematopoietic cell transplantation-specific comorbidity index
HOMIM	Human oral microbe identification microarray
HR	Hazard ratio
ICU	Intensive care unit
LDA	Linear discriminant analysis
MM	Multiple myeloma
N	Number of patients included in this study
NA	Not applicable
NK cells	Natural killer cells
NR	Not reported
NRM	Non-relapse mortality
NS	Not significant
OM	Oral mucosa
PFS	Progression-free survival
PRISMA	Preferred Reporting Items for Systematic Reviews and Meta-Analyses
RFLP	Restriction fragment length polymorphism
RT-PCR	Real-time polymerase chain reaction
SB	Supragingival biofilm

## Appendix A. Search Strategies

**Database: MEDLINE (via PubMed)**

**Search Set** **Description**	**Search Strategy**	**Results** **7 September 2022**	**Results** **15 December 2025**
#1 Stem cells	“Stem Cell Transplantation”[Mesh] OR “Bone Marrow Transplantation”[Mesh] OR ((“stem cell”[tiab] OR “stem cells”[tiab] OR “bone marrow”[tiab]) AND (transplant[tiab] OR transplants[tiab] OR transplanted[tiab] OR transplanting[tiab] OR transplantation[tiab] OR transplantations[tiab] OR post-transplant[tiab] OR post-transplants[tiab] OR post-transplantation[tiab] OR post-transplantations[tiab] OR post-transplanted[tiab] OR posttransplant[tiab] OR posttransplants[tiab] OR posttransplanted[tiab] OR posttransplantation[tiab] OR posttransplantations[tiab])) OR ASCT[tiab] OR HSCT[tiab] OR Allo-HSCT[tiab] OR AlloHSCT[tiab]	187,083	213,436
#2 Microbiome	“Microbiota”[Mesh] OR microbiome[tiab] OR microbiomes[tiab] OR microbiota[tiab] OR mycobiota[tiab] OR microbiotas[tiab] OR mycobiotas[tiab] OR mycobiome[tiab] OR mycobiomes[tiab] OR virome[tiab] OR viromes[tiab] OR bacteriome[tiab] OR bacteriomes[tiab] OR “microbial consortia”[tiab] OR “microbial consortium” OR “microbial community”[tiab] OR “microbial communities”[tiab] OR flora[tiab] OR microflora[tiab] OR floras[tiab] OR microfloras[tiab]	187,917	295,000
#3	#1 AND #2	794	1241
#4 Study design exclusion	#3 NOT (Editorial[pt] OR Comment[pt])	775	1215
#5 Search update 12/15/25	#4 AND (“2022/09/02”[Date-MeSH]: “3000”[Date-MeSH])	N/A	447

Updated search (12 January 2025): 1060 additional articles (771 duplicates and 289 new records).

**Database: Embase (via Elsevier).**

Note: Search from Results page.

**Search Set** **Description**	**Search Strategy**	**Results** **7 September 2022**	**Results** **15 December 2025**
#1 Stem cells	‘stem cell transplantation’/exp OR ‘bone marrow transplantation’/exp OR ((‘stem cell’:ti,ab OR ‘stem cells’:ti,ab OR ‘bone marrow’:ti,ab) AND (transplant:ti,ab OR transplants:ti,ab OR transplanted:ti,ab OR transplanting:ti,ab OR transplantation:ti,ab OR transplantations:ti,ab OR posttransplant:ti,ab OR posttransplants:ti,ab OR posttransplantation:ti,ab OR posttransplantations:ti,ab OR posttransplanted:ti,ab)) OR ASCT:ti,ab OR HSCT:ti,ab OR AlloHSCT:ti,ab	314,793	383,622
#2 MICROBIOME	‘microbiome’/exp OR ‘microflora’/de OR ‘bacterial flora’/exp OR ‘fungal flora’/exp OR ‘mouth flora’/exp OR microbiome:ti,ab OR microbiomes:ti,ab OR microbiota:ti,ab OR microbiotas:ti,ab OR mycobiota:ti,ab OR mycobiotas:ti,ab OR mycobiome:ti,ab OR mycobiomes:ti,ab OR virome:ti,ab OR viromes:ti,ab OR bacteriome:ti,ab OR bacteriomes:ti,ab OR ‘microbial consortia’:ti,ab OR ‘microbial consortium’:ti,ab OR ‘microbial community’:ti,ab OR ‘microbial communities’:ti,ab OR flora:ti,ab OR floras:ti,ab OR microflora:ti,ab OR microfloras:ti,ab	226,849	352,717
#3	#1 AND #2	1587	2508
#4 Study design exclusion	#3 NOT (‘editorial’/exp OR ‘note’/exp OR [note]/lim)	1542	287
#5 Search update 12/15/25	#4 AND [02-09-2022]/sd	N/A	829

Updated search (12 January 2025): 2035 additional articles (1712 duplicates and 323 new records).

**Database: Web of Science—Science Citation Index SCI 1900–present and Social Science Citation Index SSCI 1900–present (via Clarivate).**

Note: Search from Advanced.

**Search Set Description**	**Search Strategy**	**Results** **7 September 2022**	**Results** **15 December 2025**
#1 Stem cells	TS = ((“stem cell” OR “stem cells” OR “bone marrow”) AND (transplant OR transplants OR transplanted OR transplanting OR transplantation OR transplantations OR post-transplant OR post-transplants OR post-transplantation OR post-transplantations OR post-transplanted OR posttransplant OR posttransplants OR posttransplantation OR posttransplantations OR posttransplanted)) OR TS = (ASCT OR HSCT OR Allo-HSCT OR AlloHSCT)	213,945	237,253
#2 Microbiome	TS = (microbiome OR microbiomes OR microbiota OR microbiotas OR mycobiota OR mycobiotas OR mycobiome OR mycobiomes OR virome OR viromes OR bacteriome OR bacteriomes OR “microbial consortia” OR “microbial consortium” OR “microbial community” OR “microbial communities” OR flora OR floras OR microflora OR microfloras)	301,734	433,901
#3	#1 AND #2	976	1408
#4 Study design limit	#3 AND (Article or Review Article or Meeting Abstract or Proceeding Paper or Letter or Early Access (Document Types))	947	1366
#5 Search update 12/15/25	Refined by Publication Years: 2022 or 2023 or 2024 or 2025	N/A	525

## Appendix B. Joanna Briggs Critical Appraisal Tools—Cohort Studies

	**Yes**	**No**	**Unclear**	**Not Applicable**
1. Were the two groups similar and recruited from the same population? It will be assessed whether the compared groups (e.g., high versus low oral microbiota diversity; high versus low bacterial abundance; patients with and without GvHD) were recruited from the same source population, considering the same center, hospital, or clinical protocol. It will also be observed whether the groups presented comparable clinical and demographic characteristics at baseline, minimizing selection biases.	□	□	□	□
2. Were the exposures measured similarly to assign people to both exposed and unexposed groups? It will be verified whether the measurement of oral microbiota was performed in a standardized and identical manner in all groups compared, including the method of collection, storage, DNA extraction, sequencing platform, and bioinformatics pipeline, ensuring comparability between exposed and unexposed groups.	□	□	□	□
3. Was the exposure measured in a valid and reliable way? In this systematic review, the validity and reliability of oral microbiota assessment will be evaluated based on the ability of the applied methodology to comprehensively characterize the microbial community, including overall composition, diversity, and relative abundance, which are essential for robust association analyses with clinical outcomes after allo-HSCT. Studies using exclusively culture-based techniques, targeted real-time PCR, human-pathogen-focused assays, or restriction fragment length polymorphism (RFLP) will be classified as NO, as these approaches assess only predefined microbial targets and do not capture the full oral microbial community. Consequently, they limit the robustness of microbiota analyses and the identification of bacteria associated with post-allo-HSCT outcomes. In contrast, studies using 16S rRNA gene sequencing, shotgun metagenomics, or human oral microbe identification microarray (HOMIM) will be classified as YES, as these methods enable a comprehensive assessment of oral microbiota composition, allowing more reliable analyses of microbial patterns associated with GvHD, oral mucositis, and other clinical outcomes following allo-HSCT.	□	□	□	□
4. Were confounding factors identified? This item will assess whether the study identified and clearly described potential confounding factors that could influence the association between oral microbiota and clinical outcomes after allo-HSCT. Key confounders of interest include, but are not limited to, antibiotic exposure, antifungal or antiviral prophylaxis, conditioning regimen, age, sex, underlying disease, pre-transplant clinical status, oral hygiene practices or dental status, sample collection and storage procedures, and the timing of sample collection in relation to allo-HSCT. Studies will be considered adequate if they evaluated or acknowledged these variables, as well as any additional factors with the potential to affect oral microbiota composition or post-allo-HSCT outcomes, even if not all confounders were included in adjusted analyses.	□	□	□	□
5. Were strategies to deal with confounding factors stated? The study will be evaluated based on whether it clearly described the use of methodological and/or statistical strategies to control for confounding factors, especially through multivariate analyses or adjusted models. Appropriate approaches will include logistic regression, linear regression, Cox models for time to event, generalized linear models, or other multivariate models that allow simultaneous adjustment for potential confounders, such as age, sex, antibiotic use, conditioning regimen, underlying disease, and other relevant clinical variables. Additionally, it will be assessed whether the studies applied adjusted or stratified analyses to explore the impact of specific confounders, as well as the performance of sensitivity analyses to test the robustness of the results. In studies with sample or participant loss during follow-up, it will be considered whether appropriate statistical strategies were used to handle these events, such as models that accommodate censored data, analyses with justified exclusions, or other appropriate approaches. Alternatively, studies will also be considered adequate when limitations related to the control of confounding factors are explicitly acknowledged and transparently discussed by the authors.	□	□	□	□
6. Were the groups/participants free of the outcome at the start of the study (or at the moment of exposure)? Selection will be based on whether participants were free of the clinical outcome assessed at the start of follow-up or at the time of exposure, especially in the pre-allo-HSCT period, ensuring the correct timing between exposure (oral microbiota) and outcomes (GvHD, mucositis, infection).	□	□	□	□
7. Were the outcomes measured in a valid and reliable way? It will be assessed whether clinical outcomes were measured using standardized, validated, and widely accepted criteria in the literature, with clear and reproducible definitions of clinical events. For GvHD, the use of recognized classification systems will be considered adequate, such as acute GvHD graded from I to IV (e.g., modified Glucksberg criteria or NIH criteria) and chronic GvHD classified according to NIH consensus. For oral mucositis, it will be assessed whether studies used validated scales, such as the World Health Organization (WHO) classification or the Common Terminology Criteria for Adverse Events (CTCAE/NCI), with a clear definition of severity levels. In addition, other relevant clinical outcomes in the context of allo-HSCT will be considered, including relapse, progression-free survival (PFS), non-relapse-related mortality (NRM), febrile neutropenia, overall survival, infections, respiratory manifestations, and fever of unknown origin. Studies that present objective diagnostic criteria, clinical scores, or standardized definitions, as well as a consistent description of outcome measurement, will be considered methodologically adequate.	□	□	□	□
8. Was the follow up time reported and sufficient to be long enough for outcomes to occur? It will be verified whether the study clearly described the follow-up period and whether this period was sufficient to allow the development of the evaluated clinical outcomes, as observed in the literature for post-allo-HSCT events. Oral mucositis appears early after transplantation, with reports of onset generally between 3 and 30 days post-transplant and peak severity around 8–11 days after stem cell infusion. Acute GvHD is classically considered to occur up to 100 days post-transplant in many studies and classification consensuses. Chronic GvHD tends to appear later, often after the first 100 days, with reports of average development of oral manifestations around 229 days post-allo-HSCT. For relapse and long-term survival outcomes, many studies use extended follow-ups, such as 3 years (36 months), to assess relapse, PFS, and overall survival. Studies that clearly reported the follow-up time and whose duration was consistent with the expected timing of the clinical outcomes of interest will be considered methodologically adequate.	□	□	□	□
9. Was follow up complete, and if not, were the reasons to loss to follow up described and explored? It will be assessed whether participants were fully monitored throughout the study and, in cases of loss, whether the authors adequately described and discussed the reasons, such as mortality, sample collection failure, loss to clinical follow-up, or technical exclusions.	□	□	□	□
10. Were strategies to address incomplete follow up utilized? It will be assessed whether the study acknowledged the occurrence of incomplete follow-up and whether appropriate strategies were used to address missing data in order to minimize bias and preserve the validity of the results. Appropriate approaches will include a clear description of losses to follow-up, justification for missing data (e.g., death, sample collection failure, withdrawal from the study), and the application of analytical strategies to address these losses, including intention-to-treat analyses, duly justified exclusions, sensitivity analyses, or other appropriate statistical methods. Studies that do not present formal strategies for addressing incomplete follow-up may still be considered adequate if the losses are minimal and clearly described, as recommended by the JBI, or if the authors transparently discuss the potential impact of these losses on the results.	□	□	□	□
11. Was appropriate statistical analysis used? This item will evaluate whether the study applied statistical analyses appropriate for high-throughput microbiota data. Appropriate analyses must consider the biological questions, the structure of microbiome data, and the study design. Common elements of robust statistical analysis in microbiome research include: diversity analyses: use of alpha diversity indices (e.g., Shannon, Simpson) and beta diversity metrics (e.g., Bray–Curtis, UniFrac) to summarize within-sample and between-sample community differences, respectively, with appropriate statistical testing (e.g., non-parametric tests, PERMANOVA) for group comparisons. Differential abundance testing: use of methods specifically designed for sequencing count data (e.g., DESeq2, ANCOM/ANCOM-BC), which account for compositionality and multiple testing, rather than standard parametric tests alone. Multivariate analyses: ordination methods (e.g., principal coordinates analysis (PCoA), non-metric multidimensional scaling (NMDS), and models that accommodate covariates and repeated measures when relevant. Adjustment for multiple comparisons: application of appropriate correction methods (e.g., false discovery rate (FDR) correction) to control for type I error when multiple taxa or indices are tested. Model choice and assumptions: selection of statistical tests consistent with data distribution (e.g., non-parametric tests when normality assumptions are violated) and use of models that incorporate potential covariates/confounders where appropriate. Studies will be considered methodologically adequate if they demonstrate statistical approaches tailored to microbiome data characteristics, including diversity metrics, compositional data methods, and appropriate correction for multiple testing. Inappropriate use of standard parametric statistics without consideration of data properties (e.g., compositional structure) will be judged less robust.	□	□	□	□

## Appendix C. Joanna Briggs Critical Appraisal Tools—Cross-Sectional Studies

	Yes	No	Unclear	Not Applicable
1. Were the criteria for inclusion in the sample clearly defined? The evaluation will consider whether the studies presented clearly defined inclusion criteria, transparently described in the methodology, encompassing the characteristics of the study population, such as patients undergoing allo-HSCT, recruitment center(s), age range, underlying disease, and relevant clinical criteria.	□	□	□	□
2. Were the study subjects and the setting described in detail? It will be verified whether the studies provided a clear and detailed description of the evaluated population and the clinical setting, allowing for proper interpretation and comparability of results. Characterization of demographic factors, including age, biological sex, population type (adult, pediatric, or mixed), and the underlying disease that motivated allo-HSCT, such as leukemia, lymphoma, multiple myeloma, or other hematological malignancies, will be considered essential. A consistent description of these elements will be considered fundamental to contextualizing the findings on the oral microbiota in relation to clinical outcomes in the transplantation setting.	□	□	□	□
3. Was the exposure measured in a valid and reliable way? In this systematic review, the validity and reliability of oral microbiota assessment will be evaluated based on the ability of the applied methodology to comprehensively characterize the microbial community, including overall composition, diversity, and relative abundance, which are essential for robust association analyses with clinical outcomes after allo-HSCT. Studies employing exclusively culture-based techniques, targeted real-time PCR, human-pathogen-focused assays, or restriction fragment length polymorphism (RFLP) will be classified as NO, as these approaches assess only predefined microbial targets and do not capture the full oral microbial community. Consequently, they limit the robustness of microbiota analyses and the identification of bacteria associated with post-allo-HSCT outcomes. In contrast, studies using 16S rRNA gene sequencing, shotgun metagenomics, or human oral microbe identification microarray (HOMIM) will be classified as YES, as these methods enable a comprehensive assessment of oral microbiota composition, allowing more reliable analyses of microbial patterns associated with GvHD, oral mucositis, and other clinical outcomes following allo-HSCT.	□	□	□	□
4. Were objective, standard criteria used for measurement of the condition? The studies will be evaluated to determine if they used objective, standardized, and reproducible criteria for measuring conditions related to the oral microbiota, including diversity, dominance, and bacterial abundance. Studies that define these metrics based on parameters recognized in the literature, clearly linked to the methodology used, such as validated diversity indices, quantitative or relative measures of abundance, and explicit criteria for characterizing microbial dominance, will be considered adequate. The consistent use of these criteria is essential to ensure the comparability of results among the included studies.	□	□	□	□
5. Were confounding factors identified? This item will assess whether the study identified and clearly described potential confounding factors that could influence the association between oral microbiota and clinical outcomes after allo-HSCT. Key confounders of interest include, but are not limited to, antibiotic exposure, antifungal or antiviral prophylaxis, conditioning regimen, age, sex, underlying disease, pre-transplant clinical status, oral hygiene practices or dental status, sample collection and storage procedures, and the timing of sample collection in relation to allo-HSCT. Studies will be considered adequate if they evaluated or acknowledged these variables, as well as any additional factors with the potential to affect oral microbiota composition or post-allo-HSCT outcomes, even if not all confounders were included in adjusted analyses.	□	□	□	□
6. Were strategies to deal with confounding factors stated? The study will be evaluated based on whether it clearly described the use of methodological and/or statistical strategies to control for confounding factors, especially through multivariate analyses or adjusted models. Appropriate approaches will include logistic regression, linear regression, Cox models for time to event, generalized linear models, or other multivariate models that allow simultaneous adjustment for potential confounders, such as age, sex, antibiotic use, conditioning regimen, underlying disease, and other relevant clinical variables. Additionally, it will be assessed whether the studies applied adjusted or stratified analyses to explore the impact of specific confounders, as well as the performance of sensitivity analyses to test the robustness of the results. In studies with sample or participant loss during follow-up, it will be considered whether appropriate statistical strategies were used to handle these events, such as models that accommodate censored data, analyses with justified exclusions, or other appropriate approaches. Alternatively, studies will also be considered adequate when limitations related to the control of confounding factors are explicitly acknowledged and transparently discussed by the authors.	□	□	□	□
7. Were the outcomes measured in a valid and reliable way? It will be assessed whether clinical outcomes were measured using standardized, validated, and widely accepted criteria in the literature, with clear and reproducible definitions of clinical events. For GvHD, the use of recognized classification systems will be considered adequate, such as acute GvHD graded from I to IV (e.g., modified Glucksberg criteria or NIH criteria) and chronic GvHD classified according to NIH consensus. For oral mucositis, it will be assessed whether studies used validated scales, such as the World Health Organization (WHO) classification or the Common Terminology Criteria for Adverse Events (CTCAE/NCI), with a clear definition of severity levels. In addition, other relevant clinical outcomes in the context of allo-HSCT will be considered, including relapse, progression-free survival (PFS), non-relapse-related mortality (NRM), febrile neutropenia, overall survival, infections, respiratory manifestations, and fever of unknown origin. Studies that present objective diagnostic criteria, clinical scores, or standardized definitions, as well as a consistent description of outcome measurement, will be considered methodologically adequate.	□	□	□	□
8. Was appropriate statistical analysis used? This item will evaluate whether the study applied statistical analyses appropriate for high-throughput microbiota data. Appropriate analyses must consider the biological questions, the structure of microbiome data, and the study design. Common elements of robust statistical analysis in microbiome research include: diversity analyses: use of alpha diversity indices (e.g., Shannon, Simpson) and beta diversity metrics (e.g., Bray–Curtis, UniFrac) to summarize within-sample and between-sample community differences, respectively, with appropriate statistical testing (e.g., non-parametric tests, PERMANOVA) for group comparisons. Differential abundance testing: use of methods specifically designed for sequencing count data (e.g., DESeq2, ANCOM/ANCOM-BC), which account for compositionality and multiple testing, rather than standard parametric tests alone. Multivariate analyses: ordination methods (e.g., principal coordinates analysis (PCoA), non-metric multidimensional scaling (NMDS), and models that accommodate covariates and repeated measures when relevant. Adjustment for multiple comparisons: application of appropriate correction methods (e.g., false discovery rate (FDR) correction) to control for type I error when multiple taxa or indices are tested. Model choice and assumptions: selection of statistical tests consistent with data distribution (e.g., non-parametric tests when normality assumptions are violated) and use of models that incorporate potential covariates/confounders where appropriate. Studies will be considered methodologically adequate if they demonstrate statistical approaches tailored to microbiome data characteristics, including diversity metrics, compositional data methods, and appropriate correction for multiple testing. Inappropriate use of standard parametric statistics without consideration of data properties (e.g., compositional structure) will be judged less robust.	□	□	□	□
