# Supplementary material for: Oral Microbiota and Clinical Outcomes in Allogenic Hematopoietic Stem Cell Transplantation: A Systematic Review
Source: Microorganisms. 2026 Jan 28;14(2):308. doi: 10.3390/microorganisms14020308 (PMC12943532; doi:10.3390/microorganisms14020308)
Supplement: Supplementary file 1 [file microorganisms-14-00308-s001.zip › microorganisms-4076806-supplementary.pdf]

# Supplementary Files

| Supplementary Table S1. Additional studies identified from other oral microbiota reviews | |
|---|---|
| **Author, Year** | **Decision** |
| Ingham 2019[24] | Exclude<br>Oral microbiota was not evaluated |
| Taur 2012[25] | Exclude<br>Oral microbiota was not evaluated |
| Kaysen 2017[26] | Exclude<br>Oral microbiota was not evaluated |
| Peled 2020[27] | Exclude<br>Oral microbiota was not evaluated |
| Weber 2019[28] | Exclude<br>Oral microbiota was not evaluated |
| Osakabe 2017[29] | Excluded<br>Not clear if cohort was patients undergoing allo-HSCT |
| Sugita 2012[30] | Excluded<br>Oral microbiota was not evaluated |
| Pragman 2019[65] | Excluded<br>Non Allo-HSCT study |
| Bingle 2006 [66] | Excluded<br>Non Allo-HSCT study |
| Rajakaruna 2012[67] | Excluded<br>Non Allo-HSCT study |
| Kitamoto 2020[68] | Excluded<br>Non Allo-HSCT study |
| Ribeiro 2022[69] | Excluded<br>Non Allo-HSCT study |
| Liu 2021[70] | Excluded<br>Non Allo-HSCT study |
| Rothman 2023[71] | Excluded<br>Non Allo-HSCT study |
| Yamamoto 2023[16] | Excluded<br>Review article |
| De Molla 2021[2] | Already included |
| Heidrich 2021[10] | Already included |
| Ingham 2021[31] | Already included |
| Oku 2020[13] | Already included |
| Laheij 2012[32] | Already included |
| Ames 2019[33] | Already included |
| Muro 2018[34] | Already included |
| Laheij 2022[15] | Already included |
| Shouval 2020[44] | Already included |
| Gem 2024[50] | Already included |
| Rashidi 2024[40] | Already included |
| Faraci 2024[12] | Already included |
| Bruno 2022[11] | Already included |

Allo-HSCT = allogeneic hematopoietic stem cell transplantation.

| Supplementary Table S2A. Methodological Quality Assessment of Included Cohort Studies | | | | | | | | | | | | | |
|---|---|---|---|---|---|---|---|---|---|---|---|---|---|
| **Study** | **Reviewer** | **Q1** | **Q2** | **Q3** | **Q4** | **Q5** | **Q6** | **Q7** | **Q8** | **Q9** | **Q10** | **Q11** | **Total Score** |
| Brehm 2025 [48] | Jefferson | Yes | Yes | Yes | Yes | No | No | Yes | Yes | Unclear | Unclear | Yes | 7/11 |
| | Danielle | Yes | Yes | Yes | Yes | No | No | Yes | Yes | Unclear | Unclear | Yes | |
| Ebadi 2025 [49] | Jefferson | Yes | Yes | Yes | Yes | Yes | Yes | Yes | Yes | Unclear | Unclear | Yes | 9/11 |
| | Danielle | Yes | Yes | Yes | Yes | Yes | Yes | Yes | Yes | Unclear | Unclear | Yes | |
| Kambara 2025 [58] | Jefferson | Yes | Yes | Yes | Yes | Yes | Yes | Yes | Yes | Unclear | Unclear | Yes | 9/11 |
| | Danielle | Yes | Yes | Yes | Yes | Yes | Yes | Yes | Yes | Unclear | Unclear | Yes | |
| Gem 2024[50] 56 | Jefferson | Yes | Yes | Yes | Yes | Yes | Yes | Yes | Yes | Unclear | Unclear | Yes | 9/11 |
| | Danielle | Yes | Yes | Yes | Yes | Yes | Yes | Yes | Yes | Unclear | Unclear | Yes | |
| Ames 2019 [33] | Jefferson | Yes | Yes | Yes | Yes | Yes | Yes | Yes | Yes | Yes | No | Yes | 10/11 |
| | Danielle | Yes | Yes | Yes | Yes | Yes | Yes | Yes | Yes | Yes | No | Yes | |
| Ames 2012 [35] | Jefferson | Yes | Yes | Yes | Yes | Yes | Yes | Yes | Yes | Yes | Yes | Yes | 11/11 |
| | Danielle | Yes | Yes | Yes | Yes | Yes | Yes | Yes | Yes | Yes | Yes | Yes | |
| Bartha 2025 [9] | Jefferson | Yes | Yes | Yes | Yes | Yes | Yes | No | No | Yes | Yes | Yes | 9/11 |
| | Danielle | Yes | Yes | Yes | Yes | Yes | Yes | No | No | Yes | Yes | Yes | |
| Bruno 2022 [11] | Jefferson | Yes | Yes | Yes | No | No | Yes | Yes | Yes | Yes | Yes | Yes | 9/11 |
| | Danielle | Yes | Yes | Yes | No | No | Yes | Yes | Yes | Yes | Yes | Yes | |
| Chukhlovin 2019 [54] | Jefferson | Yes | Yes | No | Yes | Yes | Yes | Yes | Yes | No | No | Yes | 8/11 |
| | Danielle | Yes | Yes | No | Yes | Yes | Yes | Yes | Yes | No | No | Yes | |
| De Molla 2021 [2] | Jefferson | Yes | Yes | Yes | Yes | No | Yes | Yes | Yes | Yes | No | Yes | 9/11 |
| | Danielle | Yes | Yes | Yes | Yes | No | Yes | Yes | Yes | Yes | No | Yes | |
| Faraci 2024 [12] | Jefferson | Yes | Yes | Yes | Yes | No | Yes | Yes | Yes | Unclear | No | Yes | 8/11 |
| | Danielle | Yes | Yes | Yes | Yes | No | Yes | Yes | Yes | Unclear | No | Yes | |
| Heidrich 2021 [10] | Jefferson | Yes | Yes | Yes | Yes | Yes | Yes | Yes | Yes | Unclear | No | Yes | 9/11 |
| | Danielle | Yes | Yes | Yes | Yes | Yes | Yes | Yes | Yes | Unclear | No | Yes | |
| Heidrich 2023 [36] | Jefferson | Yes | Yes | Yes | Yes | Yes | Yes | Yes | Yes | No | No | Yes | 9/11 |
| | Danielle | Yes | Yes | Yes | Yes | Yes | Yes | Yes | Yes | No | No | Yes | |
| Parco 2019 [53] | Jefferson | Yes | Yes | Yes | Yes | No | Yes | Yes | Yes | No | No | No | 7/11 |
| | Danielle | Yes | Yes | Yes | Yes | No | Yes | Yes | Yes | No | No | No | |
| Lucas 1997 [51] | Jefferson | Yes | Yes | No | No | No | Yes | Yes | Yes | No | No | Yes | 6/11 |
| | Danielle | Yes | Yes | No | No | No | Yes | Yes | Yes | No | No | Yes | |
| Muro 2018 [34] | Jefferson | Yes | Yes | No | Yes | No | Yes | Yes | Yes | No | No | Yes | 7/11 |
| | Danielle | Yes | Yes | No | Yes | No | Yes | Yes | Yes | No | No | Yes | |
| Ingham 2021 [31] | Jefferson | Yes | Yes | Yes | Yes | Yes | Yes | Yes | Yes | No | No | Yes | 9/11 |
| | Danielle | Yes | Yes | Yes | Yes | Yes | Yes | Yes | Yes | No | No | Yes | |
| Kawajiri 2022 [38] | Jefferson | Yes | Yes | Yes | Yes | Yes | Yes | Yes | Yes | Unclear | No | Yes | 9/11 |
| | Danielle | Yes | Yes | Yes | Yes | Yes | Yes | Yes | Yes | Unclear | No | Yes | |
| Laheij 2012 [32] | Jefferson | Yes | Yes | No | Yes | No | Yes | Yes | Yes | Unclear | No | Yes | 7/11 |
| | Danielle | Yes | Yes | No | Yes | No | Yes | Yes | Yes | Unclear | No | Yes | |
| Laheij 2022 [15] | Jefferson | Yes | Yes | Yes | Yes | No | Yes | Yes | Yes | Unclear | No | Yes | 8/11 |
| | Danielle | Yes | Yes | Yes | Yes | No | Yes | Yes | Yes | Unclear | No | Yes | |
| Ohbayashi 2021 [39] | Jefferson | Yes | Yes | Yes | Yes | No | Yes | Yes | Yes | Unclear | No | Yes | 8/11 |
| | Danielle | Yes | Yes | Yes | Yes | No | Yes | Yes | Yes | Unclear | No | Yes | |
| Rashidi 2024 [40] | Jefferson | Yes | Yes | Yes | Yes | No | Yes | Yes | Yes | No | No | Yes | 8/11 |
| | Danielle | Yes | Yes | Yes | Yes | No | Yes | Yes | Yes | No | No | Yes | |

| | | | | | | | | | | | | |
|---|---|---|---|---|---|---|---|---|---|---|---|---|
| Shouval 2020 [44] | Jefferson | Yes | Yes | Yes | Yes | Yes | Yes | Yes | Yes | No | No | Yes | 9/11 |
| | Danielle | Yes | Yes | Yes | Yes | Yes | Yes | Yes | Yes | No | No | Yes | |
| Soga 2011 [45] | Jefferson | Yes | Yes | Yes | Yes | No | Yes | Yes | Yes | No | No | No | 7/11 |
| | Danielle | Yes | Yes | Yes | Yes | No | Yes | Yes | Yes | No | No | No | |
| Lucas 1997 [52] | Jefferson | Yes | Yes | No | No | No | Yes | Yes | Yes | No | No | Yes | 6/11 |
| | Danielle | Yes | Yes | No | No | No | Yes | Yes | Yes | No | No | Yes | |
| Vokurka 2020 [46] | Jefferson | Yes | Yes | Yes | Yes | Yes | Yes | Yes | Yes | No | No | Yes | 9/11 |
| | Danielle | Yes | Yes | Yes | Yes | Yes | Yes | Yes | Yes | No | No | Yes | |
| Takahashi 2020 [47] | Jefferson | Yes | Unclear | Yes | Yes | No | No | Yes | Yes | Unclear | No | Yes | 6/11 |
| | Danielle | Yes | Unclear | Yes | Yes | No | No | Yes | Yes | Unclear | No | Yes | |

Q1. Were the two groups similar and recruited from the same population?

Q2. Were the exposures measured similarly to assign people to both exposed and unexposed groups?

Q3. Was the exposure measured in a valid and reliable way?

Q4. Were confounding factors identified?

Q5. Were strategies to deal with confounding factors stated?

Q6. Were the groups/participants free of the outcome at the start of the study (or at the moment of exposure)?

Q7. Were the outcomes measured in a valid and reliable way?

Q8. Was the follow up time reported and sufficient to be long enough for outcomes to occur?

Q9. Was follow up complete, and if not, were the reasons to loss to follow up described and explored?

Q10. Were strategies to address incomplete follow up utilized?

Q11. Was appropriate statistical analysis used??

**Supplementary Table S2B. Methodological Quality Assessment of Included Cross-Sectional Studies**

| Study | Reviewer | Q1 | Q2 | Q3 | Q4 | Q5 | Q6 | Q7 | Q8 | Total Score |
|---|---|---|---|---|---|---|---|---|---|---|
| Oku 2020 [13] | Jefferson | Yes | Yes | Yes | Yes | Yes | No | Yes | Yes | 7/8 |
| | Danielle | Yes | Yes | Yes | Yes | Yes | No | Yes | Yes | |

Q1. Were the criteria for inclusion in the sample clearly defined?

Q2. Were the study subjects and the setting described in detail?

Q3. Was the exposure measured in a valid and reliable way?

Q4. Were objective, standard criteria used for measurement of the condition?

Q5. Were confounding factors identified?

Q6. Were strategies to deal with confounding factors stated?

Q7. Were the outcomes measured in a valid and reliable way?

Q8. Was appropriate statistical analysis used?

**Supplementary Table S3. Patient Demographics and Oral Microbiota Methodologies**

| Author, year<br>N | Biological Sex<br>Population Age | Underlying Diseases | Sampling Timepoints | Sample Collection Technique | Microbiota Analysis Methods |
|---|---|---|---|---|---|
| De Molla 2020[56]<br>30 | NR<br>NR | Leukemia: 18<br>Lymphoma: NR<br>MM: NR<br>Other: NR | Before Conditioning<br>At aplasia<br>At engraftment | Oral mucosa swab | 16S rRNA V3-V4 |
| Ames 2019[33]<br>4 | NR<br>Adults | Leukemia: 0<br>Lymphoma: 0<br>MM: 0<br>Other: 4 | Before Conditioning<br>At engraftment<br>D+100 | Tongue brushing | 16S rRNA V2-V4, V6-V9 |

| | | | | | |
|---|---|---|---|---|---|
| | | | Intubation* | | |
| Ames 2012[35]<br>11 | Male: 8<br>Female: 3<br>Adults | Leukemia: NR<br>Lymphoma: NR<br>MM: NR<br>Other: NR | Before Allo-HSCT<br>Neutrophil nadir<br>At Engraftment<br>Respiratory infection**<br>ICU Admission*** | Saliva, supragingival plaque and mucosal brushings | HOMIM |
| Bartha 2025[9]<br>25 | Male: 17<br>Female: 8<br>Adults | Leukemia: 16<br>Lymphoma: 3<br>MM: 1<br>Other: 5 | Before Allo-HSCT<br>After Allo-HSCT | Saliva and oral mucosa swab | 16S rRNA |
| Bruno 2022[11]<br>30 | Male: 16<br>Female: 14<br>Adults | Leukemia: 28<br>Lymphoma: 5<br>MM: 3<br>Other: 4 | Before Conditioning<br>Oral Mucositis Onset<br>Oral Mucositis Healing | Oral mucosa swab | 16S rRNA V3-V4 |
| Chukhlovin 2019[54]<br>202 | Male: 108<br>Female: 94<br>Adults and Pediatrics | Leukemia: 128<br>Lymphoma: 10<br>MM: NR<br>Other: 64 | Before Allo-HSCT<br>D+30<br>D+60<br>D+90<br>D+120 | Tongue swab | Bacterial culture |
| De Molla 2021[2]<br>30 | Male: 16<br>Female: 14<br>Adults | Leukemia: 27<br>Lymphoma: 5<br>MM: 1<br>Other: 4 | Before Conditioning<br>At Aplasia<br>At Engraftment | Tongue Swab | 16S rRNA V3-V4 |
| Diamond 2023[37]<br>95 | NR<br>Adults | Leukemia: NR<br>Lymphoma: NR<br>MM: NR<br>Other: NR | †Before Allo-HSCT<br>D+30<br>D+90 | Oral Swabs | 16s rRNA |
| Faraci 2024[12]<br>17 | NR<br>Adults and Pediatrics | Leukemia: 8<br>Lymphoma: 0<br>MM: 0<br>Other: 9 | Before Allo-HSCT<br>At Engraftment<br>D+30<br>D+100 | Mouth Vestibule Swab | 16S rRNA V2, V3, V4, V6, V7, V8, V9 |
| Ganesan 2021[57]<br>10 | NR<br>NR | Leukemia: NR<br>Lymphoma: NR<br>MM: NR<br>Other: NR | Before Allo-HSCT<br>D+60<br>D+180<br>D+360<br>cGvHD Onset | Oral Swabs | Whole Genome Shotgun |
| Heidrich 2021[10]<br>30 | Male: 16<br>Female: 14<br>Adults | Leukemia: 18<br>Lymphoma: 0<br>MM: 0<br>Other: 12 | Before Conditioning<br>At Aplasia<br>At Engraftment | Supragingival Biofilm | 16S rRNA V3-V4 |
| Heidrich 2023[36]<br>31 | Male: 17<br>Female: 14<br>Adults | Leukemia: 18<br>Lymphoma: 5<br>MM: 1<br>Other: 7 | Before Conditioning<br>At Aplasia<br>At Engraftment<br>D+30<br>D+75 | Gingival Crevicular Fluid<br>Oral Mucosa<br>Supragingival Biofilm | 16s rRNA V3-V4 |
| Parco 2019[53]<br>10 | NR<br>Pediatrics | Leukemia: NR<br>Lymphoma: NR<br>MM: NR<br>Other: NR | Before Allo-HSCT<br>D+2<br>D+16<br>D+24 | NR | 16S rRNA V1-V3 |
| Lucas 1997[51]<br>20 | NR<br>Pediatrics | Leukemia: 18<br>Lymphoma: 0<br>MM: 0<br>Other: 2 | Before Conditioning<br>D+7<br>At Engraftment<br>D+119 | Oral Rinse | Bacterial culture |
| Muro 2018[34]<br>6 | Male: 2<br>Female: 4<br>Adults | Leukemia: 4<br>Lymphoma: 0<br>MM: 0<br>Other: 2 | Before Allo-HSCT (1w)<br>After Allo-HSCT (2-3w) | Oral Swab | DGGE Bands-16S rRNA |

| | | | | | |
|---|---|---|---|---|---|
| Ingham 2021[31]<br>29 | Male: 16<br>Female: 13<br>Pediatrics | Leukemia: NR<br>Lymphoma: NR<br>MM: NR<br>Other: 29 | Before Allo-HSCT<br>D0<br>D+7<br>D+14<br>D+21<br>D+30<br>D+90<br>D+180<br>D+360 | Oral Swabs | 16S rRNA V3-V4 |
| Kawajiri 2022[38]<br>10 | Male: 6<br>Female: 4<br>Adults | Leukemia: 9<br>Lymphoma: 1<br>MM: 0<br>Other: 0 | D-21<br>D-1<br>D+7<br>D+14<br>D+21 | Subgingival plaque or gingival crevicular fluid in the periodontal pocket of the lower right first molar | rtPCR |
| Laheij 2012[32]<br>49 | Male: 27<br>Female: 22<br>Adults | Leukemia: 28<br>Lymphoma: 7<br>MM: 10<br>Other: 4 | Before Conditioning<br>Twice weekly until hospital discharge | Oral rinsing | rtPCR |
| Laheij 2022[15]<br>50 | Male: 24<br>Female: 26<br>Adults | Leukemia: 26<br>Lymphoma: 6<br>MM: 2<br>Other:16 | Before Allo-HSCT (8 weeks to days before)<br>Weekly during hospitalization<br>D+90<br>D+180<br>D+360<br>D+450 | Oral rinsing | 16S rRNA V4 |
| Ohbayashi 2021[39]<br>96 | Male: 47<br>Female: 49<br>Adults | Leukemia: 37<br>Lymphoma: 1<br>MM: 0<br>Other: 58 | At the time of fever<br>D+30 | Oral rinsing | Bacterial culture |
| Rashidi 2024[40]<br>80 | Male: 48<br>Female: 32<br>Adults | Leukemia: 38<br>Lymphoma: NR<br>MM: NR<br>Other: 42 | Before Conditioning<br>cGvHD onset<br>First cGvHD follow-up visit<br>D+360 | Oral Swab | Shotgun |
| Shouval 2019[41]<br>184 | NR<br>Adults | Leukemia: 86<br>Lymphoma: NR<br>MM: NR<br>Other: NR | D-7 to D-1<br>D0 to D+6<br>D+7 to D+13<br>D+14 to D+20<br>D+21 to D+34 | Saliva | 16S rRNA V4 |
| Shouval 2020[43]<br>184 | NR<br>Adults | Leukemia: NR<br>Lymphoma: NR<br>MM: NR<br>Other: NR | Weekly from D-7 to D+34 | Saliva | 16S rRNA V4 |
| Shouval 2020[44]<br>184 | NR<br>Adults | Leukemia: 108<br>Lymphoma: 30<br>MM: 1<br>Other: 45 | D-7 to D-1<br>D0 to D+6<br>D+7 to D+13<br>D+14 to D+20<br>D+21 to D+27<br>D+28 to D+34 | Saliva | 16S rRNA V4 |
| Shouval 2020[42]<br>184 | NR<br>Adults | Leukemia: NR<br>Lymphoma: NR<br>MM: NR<br>Other: NR | NR | Saliva | 16S rRNA V4 |
| Soga 2011[45]<br>63 | Male: 42<br>Female: 21<br>Adults | Leukemia: 33<br>Lymphoma: 19<br>MM: 0<br>Other: 11 | D-7 to D-1<br>D0 to D+6<br>D+7 to D+13<br>D+14 to D+20 | Buccal Swab | Bacterial culture |

| | | | | | |
|---|---|---|---|---|---|
| Lucas 1997[52]<br>20 | NR<br>Pediatrics | Leukemia: 18<br>Lymphoma: 0<br>MM: 0<br>Other: 2 | Before Conditioning<br>D+7<br>At Engraftment<br>D+110 to D+130 | Oral Rinse | Bacterial culture |
| Vokurka 2020[46]<br>22 | Male: 11<br>Female: 11<br>Adults | Leukemia: 16<br>Lymphoma: 1<br>MM: 2<br>Other: 1 | Weekly from hospital admission to oral mucositis resolution | Oral Swab | Bacterial culture |
| Takahashi 2020[47]<br>19 | Male: 16<br>Female: 3<br>Adults | Leukemia: 18<br>Lymphoma: NR<br>MM: NR<br>Other: 2 | Before Allo-HSCT<br>Mucositis peak<br>After engraftment | Oral Swab | T-RFLP |
| Oku 2020[13]<br>45 | Male: 26<br>Female: 19<br>Adults | Leukemia: 28<br>Lymphoma: 10<br>MM: NR<br>Other: 7 | D0 | Tongue Swab | 16S rRNA |
| Brehm 2025[48]<br>43 | Male: 19<br>Female: 24<br>Adults | Leukemia: 25<br>Lymphoma: 2<br>MM: 1<br>Other: 15 | At Admission<br>Every 3 months | Oral Saliva | 16S rRNA V1-V3 |
| Ebadi 2025[49]<br>47 | Male: 23<br>Female: 24<br>Adults | Leukemia: 38<br>Lymphoma: NR<br>MM: NR<br>Other: 9 | Baseline<br>D+14<br>D+28<br>D+84 | Supragingival Plaque<br>Oral Saliva | Shotgun |
| Kambara 2025[58]<br>31 | Male: 20<br>Female: 11<br>Not Clear | Leukemia: NR<br>Lymphoma: NR<br>MM: NR<br>Other: NR | Before Allo-HSCT<br>After Allo-HSCT | Buccal mucosa swab | 16S rRNA V3-V4 |
| Faraci 2023[55]<br>17 | Male: NR<br>Female: NR<br>Adults and Pediatrics | Leukemia: NR<br>Lymphoma: NR<br>MM: NR<br>Other: 17 | Before Allo-HSCT<br>At Engraftment<br>D+30<br>D+100 | Oral Swab | 16S rRNA (specific details NR) |
| Gem 2024[50]<br>56 | Male: 29<br>Female: 27<br>Adults | Leukemia: 43<br>Lymphoma: 5<br>MM: NR<br>Other: 8 | Baseline<br>D+7<br>D+14<br>D+21<br>D+28<br>D+84 | Supragingival Plaque<br>Gingival Crevicular Fluid<br>Subgingival Plaque<br>Oral Saliva | Shotgun |

Allo-HST = Allogeneic hematopoietic stem cell transplantation; D = Day; HOMIM = Human Oral Microbe Identification Microarray; ICU = Intensive Care Unit; NR = Not Reported; N = Number of patients; rt-PCR = Real-Time Polymerase Chain Reaction; RFLP = Restriction Fragment Length Polymorphism; * = additional sample was collected if the patient developed respiratory complications within 1 year after allo-HSCT and required intubation; ** = additional samples were obtained if respiratory signs and symptoms developed and patient required hospitalization; *** = Additional samples were collected if a patient was admitted to the ICU and intubation occurred and every 48h after that for two additional collections; † = detailed data about collection timepoints NR.

**Supplementary Table S4. Factors Impacting Oral Microbiota Over the Allo-HSCT**

| Variable | Author, year<br>N | Sample Timing | Oral Microbiota Diversity | Oral Microbiota Composition |
|---|---|---|---|---|

| | | | | |
|---|---|---|---|---|
| **Conditioning Regimen**[2,13,15,45,49,54] | Chukhlovin 2019[54] 202 | Before Allo-HSCT D+30 D+60 D+90 D+120 | **N/A** | There were no significant associations between four of the most common bacterial species and the type of conditioning regimen: *Streptococcus viridans* ($p$ = 0.54). *Neisseria spp.* ($p$ = 0.34). *Corynebacterium spp.* ($p$ = 0.11). *Klebsiella pneumoniae* ($p$ = 0.26). **Myeloablative vs. Non-myeloablative** Patients who received a myeloablative conditioning regimen had a significantly lower proportion of positive *Staphylococcus epidermidis* cultures compared to those who did not (13.6% vs. 27.3%; $p = 8 \times 10^{-5}$). |
| | Oku 2020[13] 45 | D0 | **N/A** | **Myeloablative vs. Reduced Intensity** There were no significant associations between presence of *Staphylococcus haemolyticus* and/or *Ralstonia pickettii* and the type of conditioning regimen ($p$ = 0.37). |
| | De Molla 2021[2] 30 | Before Conditioning At Aplasia At Engraftment | **Before Conditioning** No association was observed between oral microbial diversity and the type of conditioning regimen ($p$ = 1.00). **At Aplasia** No association was observed between oral microbial diversity and the type of conditioning regimen ($p$ = 0.44). **At Engraftment** No association was observed between oral microbial diversity and the type | **N/A** |

| | | | | |
|---|---|---|---|---|
| | | | of conditioning regimen (*p* = 0.69). | |
| | Laheij 2022[15] 50 | Before Allo-HSCT (8 weeks to days before) Weekly during hospitalization D+90 D+180 D+360 D+450 | **Before Conditioning** No association was observed between oral microbial diversity and the type of conditioning regiment (PERMANOVA $p > 0.001$; Mann-Whitney U Test $p > 0.05$). | **Before Conditioning** No association was observed between oral microbial composition and the type of conditioning regiment (PERMANOVA $p > 0.001$; Mann-Whitney U Test $p > 0.05$). |
| | Ebadi 2025 [49] 47 | Baseline D+14 D+28 D+84 | **TBI vs. Chemotherapy-Based Myeloablative** Saliva: Microbiota diversity had similar dynamics in both groups, with an initial decline from baseline to D+!4 and recovery by D+84. Supragingival Plaque: Groups showed significantly different microbiota diversity dynamics. In the chemotherapy-based group, α-diversity steadily | **All Samples** **TBI vs. Chemotherapy-Based Myeloablative** Saliva: No significant differences in microbiota composition were observed between groups. Supragingival Plaque: Microbiota composition was similar at baseline (p = 0.14) but differed significantly between groups at all post-HSCT time points. **D+14 (Supragingival Plaque)** **TBI** ↑ *Prevotella melaninogenica* ↑ *Actinobaculum* (NR) **Chemotherapy Based** ↑ *Streptococcus sanguinis* (NR) ↑ *Rothia* (NR) |

| | | | | |
|---|---|---|---|---|
| | | | declined with no recovery by D+84. In the TBI-based group, diversity increased by D+84, being significantly higher than in the chemotherapy group ($p$ = 0.01) | |
| | Soga 2011[45] 63 | D-7 to D-1<br>D0 to D+6<br>D+7 to D+13<br>D+14 to D+20 | **N/A** | **All Samples**<br>There were no significant differences in the detection frequencies of *Streptococcus* species or coagulase-negative *staphylococci* between patients undergoing myeloablative versus reduced-intensity conditioning. |
| **Antibiotic**[9,10,13,33,34,36,45,48] | Ames 2019[33] 4 | Before conditioning<br>At engraftment<br>D+100<br>Intubation* | Authors identified correlations between antibiotic use and oral microbiota diversity (specific data NR). | **N/A** |
| | Brehm 2025 [48] 43 | Not clear | Antibiotic exposure did not impact α or β diversity. | **N/A** |
| | Bartha 2025[9] 25 | Before Allo-HSCT<br>After Allo-HSCT | Number of antibiotics had a significant influence on β-diversity changes between saliva and mucosal samples collected before and after allo-HSCT (saliva: $R^2$ = 0.04; adjusted $p$ = 0.001; oral mucosa: $R^2$ = 0.05; adjusted $p$ = 0.001). | **N/A** |
| | Heidrich 2021[10] 30 | Before Conditioning<br>At Aplasia<br>At Engraftment | **N/A** | ***Enterococcus* Domination**<br>No significant association was found between *Enterococcus* domination and the use of cephalosporins ($p$ = 0.29) or antibiotics targeting anaerobic bacteria ($p$ = 1). |

| | | | | |
|---|---|---|---|---|
| | Oku 2020[13] 45 | D0 | **N/A** | There were a significant association between presence of *Staphylococcus haemolyticus* and/or *Ralstonia pickettii* and antibiotic use ($p = 0.001$).<br><br>PCoA plot based on unweighted UniFrac distance showed that antibiotic, rather than conditioning regimen and underlying diseases, had an impact on the overall oral bacterial composition ($p < 0.001$). |
| | Heidrich 2023[36] 31 | Before Conditioning<br>At Aplasia<br>At Engraftment<br>D+30<br>D+75 | **Diversity Stability**<br>Increased number of agent days under antibiotic therapy was significantly associated with lower diversity stability. However, the following specific antibiotic classes were not associated with altered diversity stability:<br><br>Cephalosporins<br>GCF: $p$ = 0.6636.<br>OM: $p$ = 0.2913<br>SB: $p$ = 0.1084<br><br>Carbapenems<br>GCF: $p$ = 0,9269<br>OM: $p$ = 0,3340<br>SB: $p$ = 0,5617<br><br>Glycopeptides<br>GCF: $p$ = 0,3848<br>OM: $p$ = 0,1914<br>SB: $p$ = 0,4673<br><br>Penicillin<br>GCF: $p$ = 0,4830<br>OM: $p$ = 0,4516 | **Composition Stability**<br>Number of agent days under antibiotic therapy was not associated with composition stability. Only glycopeptide usage was significantly associated with decreased compositional stability ($p = 0.0235$) in SB samples.<br>The following specific antibiotic classes were not associated with altered composition stability:<br><br>Cephalosporins<br>GCF: $p$ = 0,0848<br>OM: $p$ = 0,7440<br>SB: $p$ = 0,0553<br><br>Carbapenems<br>GCF: $p$ = 0,8990<br>OM: $p$ = 0,2970<br>SB: $p$ = 0,3500<br><br>Glycopeptides<br>GCF: $p$ = 0,2633<br>OM: $p$ = 0,9330<br>SB: $p$ = 0,0235<br><br>Penicillins<br>GCF: $p$ = 0,0812<br>OM: $p$ = 0,5050<br>SB: $p$ = 0,4417<br><br>DOT<br>GCF: $p$ = 0,4136<br>OM: $p$ = 0,6970<br>SB: $p$ = 0,2456<br><br>**Domination Event**<br>Patients experiencing domination events showed higher antibiotic length of therapy (GCF $p$ <0.05; OM $p < 0.05$; SB $p < 0.05$) and number of agent days under antibiotic therapy (GCF $p < 0.01$; OM $p < 0.05$; SB $p < 0.05$).<br><br>Domination events were also associated with the use of glycopeptides (OR 15-.65; $p = 0.006$; $p$ adjusted = 0.025). |

| | | | | |
|---|---|---|---|---|
| | | | SB: $p$ = 0,4031<br><br>DOT<br>GCF: $p$ = 0,0172<br>OM: $p$ = 0,0015<br>SB: $p$ = 0,0467 | |
| | Muro 2018[34] 6 | Before Allo-HSCT<br>After Allo-HSCT | **N/A** | **Before allo-HSCT vs. After allo-HSCT**<br><br>**B-lactam-glycopeptide**<br>The number of DGGE bands decreased markedly after allo-HSCT (12 vs. 4) in patients receiving this type of antibiotics.<br>Also, patients in this group had an increase in *Staphylococcus* and *Enterococcus* after the allo-HSCT.<br><br>**Quinolone Prophylaxis/B-lactam Monotherapy**<br>The number of DGGE bands was relatively stable after allo-HSCT (20 vs. 21) in patients from this group.<br>Also, patients in this group frequently had components of the normal oral microbiota both prior and after allo-HSCT, including *Gemella spp, Veillonella spp, Rothia spp, Actinomyces spp* and *Streptococcus spp*. |
| | Gem 2024[50] 56 | Baseline | N/A | *Oribacterium asaccharolyticum* abundance was not associated with antibiotic exposure ($p$ = 0.76). |
| | Soga 2011[45] 63 | D-7 to D-1<br>D0 to D+6<br>D+7 to D+13<br>D+14 to D+20 | **N/A** | **All Samples**<br>Across all samples, both short-term and long-term antibiotic users exhibited significant reductions in Streptococcus species ($p < 0.005$) and a significant increase in coagulase-negative staphylococci ($p < 0.05$) over the course of allo-HSCT.<br><br>**D+15 to D+21**<br>When comparing patients by short- versus long-term antibiotic use, those receiving long-term therapy demonstrated a significant decrease in Streptococcus species ($p < 0.005$) and a significant increase in coagulase-negative staphylococci ($p < 0.005$). |
| **Gender**[2,9,13,15] | Bartha 2025[9] 25 | Before Allo-HSCT<br>After Allo-HSCT | Gender had a significant influence on β-diversity changes between saliva and mucosal samples collected before and after allo-HSCT (saliva: $R^2$ = 0.03; | **N/A** |

| | | | | |
|---|---|---|---|---|
| | | | adjusted *p* = 0.022; oral mucosa: $R^2$ = 0.04; adjusted *p* = 0.014). | |
| | De Molla 2021[2] 30 | Before Conditioning<br>At Aplasia<br>At Engraftment | **Before Conditioning**<br>No association was observed between oral microbial diversity and gender (*p* = 0.13).<br><br>**At Aplasia**<br>No association was observed between oral microbial diversity and gender (*p* = 0.71).<br><br>**At Engraftment**<br>No association was observed between oral microbial diversity and gender (*p* = 0.24). | **N/A** |
| | Oku 2020[13] 45 | D0 | **N/A** | There were no significant associations between presence of *Staphylococcus haemolyticus* and/or *Ralstonia pickettii* and gender (*p* = 0.35). |
| | Gem 2024[50] 56 | Baseline | N/A | *Oribacterium asaccharolyticum* abundance was significantly higher in men than women (*p* = 0.0004). This bacterium was detected at baseline in the saliva of 20 of 29 men (69%), compared with only 5 of 27 women (19%) (*p* = 0.0002). |
| | Laheij 2022[15] 50 | Before Allo-HSCT (8 weeks to days before)<br>Weekly during hospitalization<br>D+90<br>D+180<br>D+360<br>D+450 | **Before Conditioning**<br>No association was observed between oral microbial diversity and gender (PERMANOVA *p* > 0.001; Mann-Whitney U | **Before Conditioning**<br>No association was observed between oral microbial composition and the gender (PERMANOVA *p* > 0.001; Mann-Whitney U Test *p* > 0.05). |

| | | | | |
|---|---|---|---|---|
| | | | Test $p >$ 0.05). | |
| **Age**[2,9,13,54] | Bartha 2025[9] 25 | Before Allo-HSCT After Allo-HSCT | Older age was significantly associated with a slower decline in $\alpha$-diversity (Estimate: 0.029; $p$ = 0.033). | **N/A** |
| | Chukhlovin 2019[54] 202 | Before Allo-HSCT D+30 D+60 D+90 D+120 | N/A | The frequency of common bacterial species identified in oral cultures varied according to patient age. For example, Streptococcus viridans detection was highest among children aged 0–5 years compared with those aged 6–14 and 15–21 years ($p < 0.02$). |
| | Oku 2020[13] 45 | D0 | N/A | There were no significant associations between presence of *Staphylococcus haemolyticus* and/or *Ralstonia pickettii* and age ($p$ = 0.46). |
| | Gem 2024[50] 56 | Baseline | N/A | Oribacterium asaccharolyticum abundance was not associated with age (r = -0.09; $p$ = 0.51). |
| | De Molla 2021[2] 30 | Before Conditioning At Aplasia At Engraftment | **Before Conditioning** No association was observed between oral microbial diversity and age ($p$ = 0.30).<br><br>**At Aplasia** No association was observed between oral microbial diversity and age ($p$ = 0.87).<br><br>**At Engraftment** No association was observed between oral microbial diversity and age ($p$ = 0.68). | **N/A** |
| **Body Mass Index**[9] | Bartha 2025[9] 25 | Before Allo-HSCT After Allo-HSCT | Overweight was significantly associated | **N/A** |

| | | | | |
|---|---|---|---|---|
| | | | with a slower decline in α-diversity (Estimate: 0.7157; $p$ = 0.027) and β-diversity (Estimate: -0.060; $p$ = 0.029). | |
| **DMF-T Score**[9] | Bartha 2025[9] 25 | Before Allo-HSCT After Allo-HSCT | DMF-T score was significantly associated with a slower decline in α-diversity (Estimate: 0.04092; $p$ = 0.046). | **N/A** |
| **Renal Function**[9] | Bartha 2025[9] 25 | Before Allo-HSCT After Allo-HSCT | Impaired renal function was significantly associated with a higher decline in α-diversity (Estimate: -1.06; $p$ = 0.044). | **N/A** |
| **Stem Cell Source**[2,13,54] | Chukhlovin 2019[54] 202 | Before Allo-HSCT D+30 D+60 D+90 D+120 | **N/A** | There were no significant associations between the most common bacterial species and the source of stem cells: *Streptococcus viridans* ($p$ = 0.39). *Staphylococcus epidermidis* ($p$ = 0.84). *Neisseria spp.* ($p$ = 0.90). *Corynebacterium spp.* ($p$ = 0.87). *Klebsiella pneumoniae* ($p$ = 0.20). |
| | Oku 2020[13] 45 | D0 | **N/A** | There were no significant associations between presence of *Staphylococcus haemolyticus* and/or *Ralstonia pickettii* and graft source ($p$ = 0.07). |
| | De Molla 2021[2] 30 | Before Conditioning At Aplasia At Engraftment | **Before Conditioning** No association was observed between oral microbial diversity and stem cell source ($p$ = 1.00). **At Aplasia** No association was observed between oral | **N/A** |

| | | | | |
|---|---|---|---|---|
| | | | microbial diversity and stem cell source ($p$ = 1.00).<br><br>**At Engraftment** No association was observed between oral microbial diversity and stem cell source ($p$ = 1.00). | |
| **Underlying Disease**[2,12,13] | Faraci 2024[12] 17 | Before Allo-HSCT At Engraftment | **Malignant vs. Non-malignant** Patients with malignant diseases exhibited lower $\alpha$-diversity values compared to those with non-malignant diseases (specific details NR).<br><br>Samples collected during the engraftment period showed a similar trend, except for the Chao1 index, which displayed higher values in patients with malignant diseases (26.07 vs. 20.14). | **N/A** |
| | Gem 2024[50] 56 | Baseline | N/A | *Oribacterium asaccharolyticum* abundance was not associated with the underlying disease ($p$ = 0.95). |
| | Oku 2020[13] 45 | D0 | **N/A** | There were no significant associations between presence of *Staphylococcus haemolyticus* and/or *Ralstonia pickettii* and underlying disease ($p$ = 0.73). |

| | | | | |
|---|---|---|---|---|
| | De Molla 2021[2] 30 | Before Conditioning At Aplasia At Engraftment | **Before Conditioning** No association was observed between oral microbial diversity and underlying disease ($p$ = 1.00).<br><br>**At Aplasia** No association was observed between oral microbial diversity and underlying disease ($p$ = 0.70).<br><br>**At Engraftment** No association was observed between oral microbial diversity and underlying disease ($p$ = 0.43). | **N/A** |
| **HCT-CI**[2] | De Molla 2021[2] 30 | Before Conditioning At Aplasia At Engraftment | **Before Conditioning** No association was observed between oral microbial diversity and HCT-CI Score ($p$ = 0.24).<br><br>**At Aplasia** No association was observed between oral microbial diversity and HCT-CI Score ($p$ = 1.00). | **N/A** |

| | | | | |
|---|---|---|---|---|
| | | | **At Engraftment** No association was observed between oral microbial diversity and HCT-CI Score ($p$ = 0.15). | |
| **Risk Status at allo-HSCT**[2,13] | De Molla 2021[2] 30 | Before Conditioning At Aplasia At Engraftment | **Before Conditioning** No association was observed between oral microbial diversity and disease risk index ($p$ = 0.45).<br><br>**At Aplasia** No association was observed between oral microbial diversity and disease risk index ($p$ = 1.00).<br><br>**At Engraftment** No association was observed between oral microbial diversity and disease risk index ($p$ = 0.43). | **N/A** |
| | Oku 2020[13] 45 | D0 | **N/A** | There were no significant associations between the presence of *Staphylococcus haemolyticus* and/or *Ralstonia pickettii* and risk status at allo-HSCT ($p$ = 0.06). |
| **Total body irradiation**[2,13] | De Molla 2021[2] 30 | Before Conditioning At Aplasia At Engraftment | **Before Conditioning** No association was observed between oral microbial diversity and total | **N/A** |

| | | | | |
|---|---|---|---|---|
| | | | body irradiation ($p = 0.42$).<br><br>**At Aplasia**<br>No association was observed between oral microbial diversity and total body irradiation ($p = 0.69$).<br><br>**At Engraftment**<br>No association was observed between oral microbial diversity and total body irradiation ($p = 0.69$). | |
| | Oku 2020[13] 45 | D0 | **N/A** | There were no significant associations between presence of *Staphylococcus haemolyticus* and/or *Ralstonia pickettii* and total body irradiation ($p = 0.72$). |
| **Smoking**[15] | Laheij 2022[15] 50 | Before Allo-HSCT (8 weeks to days before)<br>Weekly during hospitalization<br>D+90<br>D+180<br>D+360<br>D+450 | **Before Conditioning**<br>No association was observed between oral microbial diversity and smoking habits (PERMANOVA $p > 0.001$; Mann-Whitney U Test $p > 0.05$). | **Before Conditioning**<br>No association was observed between oral microbial composition and smoking habits (PERMANOVA $p > 0.001$; Mann-Whitney U Test $p > 0.05$). |
| **T-cell depletion**[2] | De Molla 2021[2] 30 | Before Conditioning<br>At Aplasia<br>At Engraftment | **Before Conditioning**<br>No association was observed between oral microbial diversity and T-cell depletion ($p = 1.00$). | **N/A** |

| | | | | |
|---|---|---|---|---|
| | | | **At Aplasia** No association was observed between oral microbial diversity and T-cell depletion (*p* = 1.00).<br><br>**At Engraftment** No association was observed between oral microbial diversity and T-cell depletion (*p* = 0.70). | |
| **Transplant Type**[2,13] | De Molla 2021[2] 30 | Before Conditioning At Aplasia At Engraftment | **Before Conditioning** No association was observed between oral microbial diversity and transplant type (*p* = 0.84).<br><br>**At Aplasia** No association was observed between oral microbial diversity and transplant type (*p* = 1.00).<br><br>**At Engraftment** No association was observed between oral microbial diversity and transplant type (*p* = 0.88). | **N/A** |

| | | | | |
|---|---|---|---|---|
| | Oku 2020[13] 45 | D0 | **N/A** | There were no significant associations between presence of *Staphylococcus haemolyticus* and/or *Ralstonia pickettii* and transplant type (HLA parity; *p* = 0.23). |
| **Immune System**[31,48] | Ingham 2021[31] 29 | At Preexamination<br>D0<br>D+7<br>D+14<br>D+21<br>D+30<br>D+90<br>D+180<br>D+360 | **N/A** | **D+90 and D+180**<br>CD4⁺ T cell counts on days 90 and 180 after allo-HSCT showed a positive correlation with families of *Flavobacteriaceae, Prevotellaceae, Veillonellaceae* e *Neisseriaceae* (NR)<br><br>**D+30 and D+90**<br>The CD4⁺Th17⁺ T cell count on days 30 and 90 after allo-HSCT showed a positive correlation with families of *Flavobacteriaceae, Prevotellaceae, Veillonellaceae* e *Neisseriaceae* (NR)<br><br>**Late Samples (Details NR)**<br>ASV 422 *Actinomyces odontolyticus* and ASV 546 *Veillonella parvula* positively correlated with NK-cell counts, while ASVs affiliated with *Staphylococcaceae* and *Lactobacillaceae* were negatively correlated. |
| | Brehm 2025 [48] 43 | Not clear | β-diversity differed significantly according to the levels of ILRL1 (*p* = 0.38), WFDC2 (*p* = 0.036), CXCL9 (*p* = 0.009), DKK3 (*p* = 0.037), and CCL15 (*p* =0.008).. | **N/A** |

Allo-HST = Allogeneic hematopoietic stem cell transplantation; ASV = Amplicon Sequence Variant; D = Day; DMF-T = Decayed, Missing, Filled Teeth Score; DOT = days of therapy; GCF = gingival crevicular fluid; HCT-CI = hematopoietic cell transplantation-specific comorbidity index; N = number of patients; N/A = Not Applicable; NK-cells = Natural Killers cells; NR = Not Reported; OM = oral mucosa; SB = supragingival biofilm; ↓ = Decreased; ↑ = increased; * = additional sample was collected if the patient developed respiratory complications within 1 year after allo-HSCT and required intubation.

**Supplementary Table S5. Studies Evaluating the Dynamics of Oral Microbiota Over the Allo-HSCT**

| Author, year N | Samples Timing | Oral Diversity | Oral Composition | Domination |
|---|---|---|---|---|
| Ames 2019[33] 4 | Before Conditioning<br>At Engraftment<br>D+100<br>Intubation* | **Before Conditioning vs At Engraftment**<br>↓ Shannon Diversity (3.0 vs. 1.4; *p* value NR).<br><br>**At Engraftment vs D+100**<br>↑ Shannon Diversity (1.4 vs. 2.3; *p* value NR). | **Allo-HSCT Samples vs. Controls**<br>When compared to controls, allo-HSCT patients had 229 unique bacterial taxa identified.<br><br>**Before Conditioning vs. At Engraftment**<br>↓ *Haemophilus parainfluenzae* (values NR; *p* = 0,049).<br>↑ *Rothia mucilaginosa* (values NR; *p* = 0,049).<br><br>**At Engraftment vs. D+100** | **N/A** |

| | | | | |
|---|---|---|---|---|
| | | **Before Conditioning vs. D+100**<br>↓ Shannon Diversity (3.0 vs. 2.3; *p* value NR). | ↑ *Haemophilus parainfluenzae* (values NR; *p* = 0,05).<br>↓ *Rothia mucilaginosa* (values NR; *p* < 0,05). | |
| Ames 2012[35]<br>11 | Before Allo-HSCT<br>Neutrophil Nadir<br>At Engraftment<br>Respiratory infection**<br>ICU Admission*** | **N/A** | **Before Allo-HSCT vs. After Allo-HSCT**<br>Despite differences in the relative number of positive probes for certain bacterial taxa, principal component analysis demonstrated considerable overlap between the groups, suggesting an absence of clear separation between the pre- and post–allo-HSCT samples. | **N/A** |
| Bartha 2025[9]<br>25 | Before Allo-HSCT<br>After Allo-HSCT | **Before Allo-HSCT vs. After Allo-HSCT**<br>A significant change in β-diversity was observed in both saliva and oral mucosal samples (saliva: $R^2$ = 0.10, adjusted *p* = 0.001; oral mucosa: $R^2$ = 0.07, adjusted *p* = 0.001).<br><br>**Before Allo-HSCT vs. After Allo-HSCT**<br>Saliva: ↓ Shannon Diversity (values NR; adjusted *p* < 0.01).<br>Oral mucosa: ↓ Shannon Diversity (values NR; adjusted *p* < 0.01). | **N/A** | **Before Allo-HSCT vs. After Allo-HSCT**<br>Saliva: ↑ Dominance prevalence (*p* values NR).<br>Oral mucosa: ↑ Dominance prevalence (*p* values NR). |
| Bruno 2022[11]<br>30 | Before Conditioning<br>Oral Mucositis Onset<br>Oral Mucositis Healing | **Before Conditioning vs. Oral Mucositis Onset**<br>Similar $\alpha$-Diversity (values NR; *p* = 0.11).<br><br>**Before Conditioning vs. Oral Mucositis Healing**<br>↓ $\alpha$-Diversity (values NR; *p* = 0.019).<br><br>**Oral Mucositis Onset vs. Oral Mucositis Healing**<br>Similar $\alpha$-Diversity (values NR; *p* = 0.21).<br><br>**All Samples**<br>*B-d*iversity significantly differed between timepoints (*F* = 1.23; *p* = 0.015) | **Before Conditioning vs. Oral Mucositis Onset**<br>↑ *Lactobacillus* (*p* < 0.001).<br>↑ *Mycoplasma* (*p* < 0.001).<br>↑ *Parvimonas* (*p* < 0.05).<br>↓ *Catonella* (*p* < 0.001).<br><br>**Before Conditioning vs. Oral Mucositis Healing**<br>↑ *Lactobacillus* (*p* < 0.001).<br>↑ *Enterococcus* (*p* < 0.001).<br>↑ Unclassified *Bifidobacteriaceae* (*p* < 0.001).<br>↑ *Treponema* (*p* < 0.001).<br>↑ *Lactococcus* (*p* < 0.05).<br>↑ *Staphylococcus* (*p* < 0.001).<br>↓ *Porphyromonas* (*p* < 0.001).<br>↓ *Haemophilus* (*p* < 0.001).<br>↓ *Lachnoaerobaculum* (*p* < 0.001).<br>↓ *Neisseria* (*p* < 0.001).<br>↓ *Bergeyella* (*p* < 0.001).<br><br>**Oral Mucositis Onset vs. Oral Mucositis Healing**<br>↑ *Delftia* (*p* < 0.05).<br>↓ *Porphyromonas* (*p* < 0.001). | **N/A** |

| | | | | |
|---|---|---|---|---|
| Chukhlovin 2019[54] 202 | Before Allo-HSCT<br>D+30<br>D+60<br>D+90<br>D+120 | **N/A** | **Before Allo-HSCT vs. D+30**<br>A significant drop was evident for all the detectable microbial species during the first month.<br>↓ *Streptococcus viridans* (50% vs. 29.5%; *p* value NR).<br>↓ *Staphylococcus epidermidis* (16% vs. 10.3%; *p* value NR).<br>↓ *Neisseria spp.* (12% vs. 6.2%; *p* value NR).<br>↓ *Corynebacterium spp.* (11.1% vs. 6.9%; *p* value NR).<br>↓ *Klebsiella pneumoniae* (5% vs. 0.7%; *p* value NR). | **N/A** |
| Oku 2020[13] 45 | D0 | **D0 vs. Age-Matched Controls**<br>Patients undergoing allo-HSCT had significantly lower α-diversity index<br>Phylogenetic diversity: 4.0 ± 1.1 vs. 6.9 ± 1.1 ($p < 0.001$).<br>Shannon: 2.1 ± 0.6 vs. 3.2 ± 0.3 ($p < 0.001$). | **D0 vs. Age-Matched Controls**<br>PCoA plot demonstrated that bacterial composition was significant different between these two groups.<br>Patients undergoing allo-HSCT had significantly lower relative abundance of the following genera: *Streptococcus* ($p < 0.05$), *Prevotella* ($p < 0.05$), *Haemophilus* (($p < 0.05$), *Alloprevotella* ($p < 0.05$), *Leptotrichia* ($p < 0.05$), *Oribacterium* ($p < 0.05$), and *Neisseria* ($p < 0.05$).<br>Patients undergoing allo-HSCT had bacterial taxa corresponding to 12 OTU that were absent in age-matched controls:<br>*Lactobacillus paracasei* (716)<br>*Staphylococcus haemolyticus* (120)/*hominis* (127)<br>*Ralstonia pickettii* (716)/sp. (406)<br>Genus *Xanthomonas*<br>Genus *Clostridium sensu stricto*<br>*Enterococcus durans* (880)<br>*Lactobacillus rhamnosus* (749)/casei (568)<br>*Corynebacterium tuberculostearicum* (077)<br>Genus *Acinetobacter*<br>*Enterococcus faecalis* (604)<br>Genus *Capnocytophaga*<br>Genus *Pseudomonas* | **D0**<br>Reported that three patients had domination by *Staphylococcus haemolyticus*. |
| De Molla 2021[2] 30 | Before Conditioning<br>At Aplasia<br>At Engraftment | **Before Conditioning vs. At Aplasia**<br>↓ α-Diversity (Value NR; $p < 0.01$).<br><br>**Before Conditioning vs. At Engraftment**<br>↓ α-Diversity (Value NR; $p < 0.0001$). | **Before Conditioning vs. At Aplasia**<br>↑ *Enterococcus* (values NR, *p* value NR).<br>↑ *Lactobacillus* (values NR, *p* value NR).<br>↓ *Haemophilus* (values NR, *p* value NR).<br><br>**Before Conditioning vs. At Engraftment**<br>↑ *Enterococcus* (values NR, *p* value NR).<br>↑ *Lactobacillus* (values NR, *p* value NR).<br>↑ *Staphylococcus* (values NR, *p* value NR).<br>↑ *Mycoplasma* (values NR, *p* value NR).<br>↓ *Gemella* (values NR, *p* value NR). | **Before Conditioning**<br>Sixteen patients (59%) had some type of bacterial dominance at preconditioning.<br>*Streptococcus* (56%; n = 9/16)<br>*Neisseria* (19%; n = 3/16)<br>*Rothia* (12%; n = 2/16)<br>*Veillonella* (12%; n = 2/16)<br><br>**At Aplasia and At Engraftment**<br>All patients showed bacterial dominance by a single genus |

| | | | | |
|---|---|---|---|---|
| | | | | after preconditioning. |
| Faraci 2024[12] 17 | Before allo-HSCT<br>At Engraftment<br>D+30<br>D+100 | **Before Allo-HSCT vs. At Engraftment**<br>Chao1: ↓ Diversity (values NR)<br>Shannon: ↓ Diversity (values NR)<br>Simpson: ↓ Diversity (values NR)<br><br>**Before Allo-HSCT vs. D+30**<br>Chao1: ↓ Diversity (values NR)<br>Shannon: ↓ Diversity (values NR)<br>Simpson: ↓ Diversity (values NR)<br><br>**All Samples**<br>Chao1: $p = 0.047133$<br>Shannon: $p = 0.047946$<br>Simpson: $p = 0.02763$<br>Overall, patients exhibited the lowest diversity values at the time of engraftment, which then gradually increased, reaching their highest levels after day +100.<br><br>**All Samples**<br>*Β*-diversity was similar across all time points ($p = 0.209$). | **Before Allo-HSCT vs. At Engraftment**<br>The following bacteria had higher abundance in samples from before allo-HSCT:<br>↑ *Actinomyces viscosus* (FDR = 0.0076)<br>↑ *Atopobium* (FDR = 0.0043)<br>↑ *Tannerella* (FDR = 0.0142)<br>↑ *Tannerella sp* (FDR = 0.0022)<br>↑ *Capnocytophaga gingivalis* (FDR = 0.0103)<br>↑ *Prevotella loescheii* (FDR = 0.0052)<br>↑ *Prevotella sp* (FDR = 0.0093)<br>↑ *Streptococcus australis* (FDR = 0.0249)<br>↑ *Streptococcus caballi* (FDR = 0.0430)<br>↑ *Streptococcus lactarius* (FDR = 0.0383)<br>↑ *Streptococcus salivarius* (FDR = 0.0202)<br>↑ *Streptococcus sanguinis* (FDR = 0.0016)<br>↑ *Streptococcus sinensis* (FDR = 0.0168)<br>↑ *Oribacterium* (FDR = 0.0066)<br>↑ *Oribacterium sinus* (FDR = 0.00002)<br>↑ *Stomatobaculum* (FDR = 0.0142)<br>↑ *Stomatobaculum longum* (FDR = 0.0090)<br>↑ *Bulleidia* (FDR = 0.0028)<br>↑ *Megasphaera* (FDR = 0.0312)<br>↑ *Neisseria perflava* (FDR = 0.0024)<br><br>The following bacteria had higher abundance in samples at engraftment:<br>↑ *Actinomyces lingnae* (FDR = 0.0022)<br>↑ *Corynebacteriaceae* (FDR = 0.00073)<br>↑ *Corynebacterium* (FDR = 0.0055)<br>↑ *Corynebacterium durum* (FDR = 0.0035)<br>↑ *Bacteroides* (FDR = 0.0127)<br>↑ *Paraprevotellaceae* (FDR = 0.00008)<br>↑ *Porphyromonas* (FDR = 0.0319)<br>↑ *Porphyromonas catoniae* (FDR = 0.0090)<br>↑ *Prevotella copri* (FDR = 0.0377)<br>↑ *Prevotella oulorum* (FDR = 0.020)<br>↑ *Prevotella salivae* (FDR = 0.0090)<br>↑ *Firmicutes* (FDR = 0.0021)<br>↑ *Gemella morbillorum* (FDR = 0.0090)<br>↑ *Lactobacillales* (FDR = 0.0478)<br>↑ *Aerococcaceae* (FDR = 0.0156)<br>↑ *Abiotrophia* (FDR = 0.0142)<br>↑ *Streptococcus* (FDR = 0.0371)<br>↑ *Streptococcus mitis* (FDR = 0.0170)<br>↑ *Clostridia* (FDR = 0.0171)<br>↑ *Clostridiale*s (FDR = 0.0478)<br>↑ *Catonella* (FDR = 0.0142)<br>↑ *Peptostreptococcacea*e (FDR = 0.0433)<br>↑ *Peptostreptococcus* (FDR = 0.0451)<br>↑ *Peptostreptococcus stomatis* (FDR = 0.0471)<br>↑ *Ruminococcaceae* (FDR = 0.00044) | **N/A** |

| | | | | |
|---|---|---|---|---|
| | | | ↑ *Negativicutes* (FDR = 0.0171)<br>↑ *Selenomonadales* (FDR = 0.0012)<br>↑ *Veillonellaceae* (FDR = 0.0017)<br>↑ *Veillonella* (FDR = 0.0253)<br>↑ *Veillonella alcalescens* (FDR = 0.0016)<br>↑ *Veillonella parvula* (FDR = 0.0090)<br>↑ *Veillonella sp* (FDR = 0.0249)<br>↑ *Leptotrichia buccalis* (FDR = 0.0249)<br>↑ *Eikenella corrodens* (FDR = 0.0014)<br>↑ *Campylobacterales* (FDR = 0.0438)<br>↑ *Campylobacter* (FDR = 0.0142)<br>↑ *Campylobacter gracilis* (FDR = 0.0000066)<br>↑ *Enterobacteriaceae* (FDR = 0.0181)<br>↑ *Serratia* (FDR = 0.0127)<br><br>**Before Allo-HSCT vs. D+30**<br>The following bacteria had higher abundance in samples at D+30:<br>↑ *Actinomyces lingnae* (FDR = 0.0047)<br>↑ *Porphyromonas catoniae* (FDR = 0.0274)<br>↑ *Paraprevotellaceae* (FDR = 0.0472)<br>↑ *Prevotella oris* (FDR = 0.0141)<br>↑ Firmicutes (FDR = 0.0000004)<br>↑ *Bacilli* (FDR = 0.0209)<br>↑ *Gemella* (FDR = 0.0052)<br>↑ *Gemella morbillorum* (FDR = 0.00099)<br>↑ *Streptococcaceae* (FDR = 0.0472)<br>↑ *Streptococcus* (FDR = 0.0273)<br>↑ *Streptococcus oralis* (FDR = 0.0141)<br>↑ *Catonella* (FDR = 0.0038)<br>↑ *Catonella morbi* (FDR = 0.0047)<br>↑ *Peptostreptococcaceae* (FDR = 0.0472)<br>↑ *Negativicutes* (FDR = 0.0000019)<br>↑ *Selenomonadales* (FDR = 0.0000028)<br>↑ *Veillonellaceaea* (FDR = 0.000044)<br>↑ *Enterobacteriaceae* (FDR = 0.0420)<br>↑ *Serratia* (FDR = 0.0141)<br><br>The following bacteria had higher abundance in samples from before allo-HSCT<br>↑ *Rothia aeria* (FDR = 0.0107)<br>↑*Rothia dentocariosa* (FDR = 0.0141)<br>↑ *Neisseria perflava* (FDR = 0.0141)<br><br>**Before Allo-HSCT vs. D+100**<br>The following bacteria had higher abundance in samples at D+100:<br>↑ *Corynebacteriaceae* (FDR = 0.0014)<br>↑ *Corinebacterium* (FDR = 0.0014)<br>↑ *Corinebacterium durum* (FDR = 0.0183)<br>↑ *Lachnoanaerobaculim* (FDR = 0.00011)<br>↑ *Lachnoanaerobaculim sp* (FDR = 0.0053)<br>↑ *Lachnoanaerobaculim umeanse* (FDR = 0.0305)<br>↑ *Veillonella alcalescens* (FDR = 0.0053)<br>↑ *Campylobacte*r (FDR = 0.0039)<br>↑ *Campylobacter gracilis* (FDR = 0.0000018) | |

| | | | | |
|---|---|---|---|---|
| | | | The following bacteria had higher abundance in samples from before allo-HSCT:<br>↑ Fusobacterium peridonticum (FDR = 0.0053)<br>↑ Enterobacteriales (FDR = 0.0024)<br>↑ Enterobacteriaceae (FDR = 0.0148)<br><br>**All Samples**<br>↓ *Actinobacteria* (11% vs. 9% vs. 7% vs. 7%).<br>↓ *Bacteroidetes* (17% vs. 14% vs. 11% vs. 9%).<br>↓ *Fusobacteria* (6% vs. 6% vs. 5% vs. 3%).<br>↓ *Proteobacteria* (20% vs. 16% vs. 17% vs. 10%).<br>↑ *Firmicutes* (46% vs. 55% vs. 60% vs. 72%). | |
| Heidrich 2021[10]<br>30 | Before Conditioning<br>At Aplasia<br>At Engraftment | **Before Conditioning vs. At Aplasia**<br>↓ Shannon (4.15 vs. 3.39; $p = 0.009$).<br>↓ Gini-Simpson (values NR; $p = 0.013$).<br>↓ ASVs: (values NR; $p = 0.009$).<br><br>**Before Conditioning vs. At Engraftment**<br>↓ Shannon (4.15 vs. 2.75; $p < 0.001$).<br>↓ Gini-Simpson (values NR; $p < 0.001$).<br>↓ ASVs: (values NR; $p < 0.001$).<br><br>**At Aplasia vs. At Engraftment**<br>↓ Shannon (3.39 vs. 2.75; $p < 0.013$).<br>↓ Gini-Simpson (values NR; $p = 0.039$).<br>ASVs: (value NR; $p = 0.029$). | **Before Conditioning vs. At Engraftment**<br>↓ *Gemella* (ANCOM test W >0.7)<br>↓ *Lachnoanaerobaculum* (ANCOM test W >0.7).<br>↓ *Streptococcus* (ANCOM test W >0.7).<br>↓ *Leptotrichia* (ANCOM test W >0.7).<br>↓ *Actinomyces* (ANCOM test W >0.7).<br>↑ *Lactobacillus* (ANCOM test W >0.7).<br>↑ *Staphylococcus* (ANCOM test W >0.7).<br>↑ *Mycoplasma* (ANCOM test W >0.7).<br>↑ *Enterococcus* (ANCOM test W >0.7).<br>↑ *Leuconostoc* (ANCOM test W >0.7). | **All Samples**<br>A total of 20 (67%) patients experienced 23 domination events involving 12 different genera.<br><br>*Enterococcus* domination was the most frequent event and occurred in 20% of patients undergoing allo-HSCT. Other genera that contributed to domination events were:<br>*Rothia* (n = 3).<br>*Lactobacillus* (n = 3).<br>*Prevotella* 7 (n = 2).<br>*Mycoplasma* (n = 2).<br>*Prevotella* (n =1).<br>*Staphylococcus* (n = 1).<br>*Granullcatella* (n = 1).<br>*Leuconostoc* (n = 1).<br>*Johnsonella* (n = 1).<br>*Lautropia* (n = 1).<br>*Stenotrophomonas* (n = 1). |
| Heidrich 2023[36]<br>31 | Before Conditioning<br>At Aplasia<br>At Engraftment<br>D+30<br>D+75 | **Before Conditioning vs. At Aplasia**<br>GCF: ↓ Gini-Simpson (values NR; $p < 0.05$).<br>OM: ↓ Gini-Simpson (values NR; $p < 0.05$).<br>SB: ↓ Gini-Simpson (values NR; $p < 0.05$).<br><br>**Before Conditioning vs. At Engraftment**<br>GCF: ↓ Gini-Simpson (values NR; $p < 0.001$).<br>OM: ↓ Gini-Simpson (values NR; $p < 0.0001$). | **Distance to Centroid**<br>**Before Conditioning vs. At Aplasia**<br>GCF: Distance to centroid ($p$ value NR).<br>OM: Distance to centroid ($p < 0.0001$).<br>SB: Distance to centroid ($p$ value NR).<br><br>**Before Conditioning vs. At Engraftment**<br>GCF: Distance to centroid ($p < 0.005$).<br>OM: Distance to centroid ($p < 0.0001$).<br>SB: Distance to centroid ($p < 0.005$).<br><br>**Before Conditioning vs. D+30** | **All Samples**<br>A total of 27 (87%) patients experienced 81 domination events involving 22 genera.<br><br>Domination events typically occurred at engraftment (53%) and rapidly resolved in the post-engraftment period.<br><br>Most common genera associated |

| | | | | |
|---|---|---|---|---|
| | | SB: ↓ Gini-Simpson (values NR; $p < 0.0001$).<br><br>**Before Conditioning vs. D+30**<br>GCF: ↓ Gini-Simpson (values NR; $p < 0.01$).<br>OM: Gini-Simpson *p* value NR.<br>SB: ↓ Gini-Simpson (values NR; $p < 0.05$).<br><br>**Before Conditioning vs. D+75**<br>GCF: Gini-Simpson (values NR; $p < 0.08$).<br>OM: Gini-Simpson *p* value NR.<br>SB: Gini-Simpson *p* value NR. | GCF: Distance to centroid ($p = 0.009$).<br>OM: Distance to centroid ($p < 0.0001$).<br>SB: Distance to centroid (*p* value NR).<br><br>**Before Conditioning vs. D+75**<br>GCF: Distance to centroid (*p* value NR).<br>OM: Distance to centroid (*p* value NR).<br>SB: Distance to centroid (*p* value NR).<br>Overall, distance to centroid increased up to engraftment and decreased in the post-engraftment period, indicating a displacement from and posterior recovery to baseline compositions.<br><br>**Compositional Distance (PERMANOVA)**<br>**Before Conditioning vs. At Aplasia**<br>GCF: PERMANOVA $p < 0.05$.<br>OM: PERMANOVA $p < 0.05$.<br>SB: PERMANOVA $p < 0.05$.<br><br>**Before Conditioning vs. At Engraftment**<br>GCF: PERMANOVA $p < 0.05$.<br>OM: PERMANOVA $p < 0.05$.<br>SB: PERMANOVA $p < 0.05$.<br><br>**Before Conditioning vs. D+30**<br>GCF: PERMANOVA $p < 0.05$.<br>OM: PERMANOVA $p > 0.05$.<br>SB: PERMANOVA $p < 0.05$.<br><br>**Before Conditioning vs. D+75**<br>GCF: PERMANOVA $p < 0.05$.<br>OM: PERMANOVA $p > 0.05$.<br>SB: PERMANOVA $p < 0.05$.<br>Overall, composition distance using PERMANOVA showed that GCF and SB samples post-engraftment still with significantly different composition after engraftment, while OM samples more fully recovered to their before conditioning state.<br><br>**ANCOM-BC**<br>**Before Conditioning vs. At Aplasia**<br><br>GCF<br>↓ *Neisseria* ($p < 0.01$).<br><br>OM<br>↑ *Enterococcus* ($p < 0.001$).<br>↑ *Lactobacillus* ($p < 0.001$).<br>↑ *Bifidobacteriaceae* ($p < 0.01$).<br><br>SB<br>↑ *Enterococcus* ($p < 0.001$).<br>↑ *Lactobacillus* ($p < 0.001$).<br>↑ *Atopobium* ($p < 0.01$).<br>↑ *Mycoplasma* ($p < 0.01$). | with domination events were:<br>*Lactobacillus* (15%).<br>*Enterococcus* (12%).<br>*Staphylococcus* (10%). |

| | | | | |
|---|---|---|---|---|
| | | | **ANCOM-BC**<br>**Before Conditioning vs. At Engraftment**<br><br>GCF<br>↓ *Gemella* ($p < 0.05$).<br>↓ *Streptococcus* ($p < 0.001$).<br>↓ *Leptotrichia* ($p < 0.05$).<br>↓ *Neisseria* ($p < 0.001$).<br>↑ *Lactobacillus* ($p < 0.001$).<br>↑ *Mycoplasma* ($p < 0.001$).<br>↑ *Staphylococcus* ($p < 0.001$).<br><br>OM<br>↓ *Gemella* ($p < 0.001$).<br>↑ *Enterococcus* ($p < 0.001$).<br>↑ *Lactobacillus* ($p < 0.001$).<br>↑ *Bifidobacteriaces* ($p <0.001$).<br>↑ *Lactococcus* ($p < 0.01$).<br>↑ *Treponema* 2 ($p < 0.001$).<br>↑ *Prevotella* 2 ($p < 0.001$).<br>↑ *Peptostreptococcus* ($p < 0.05$).<br><br>SB<br>↓ *Leptotrichia* ($p < 0.001$).<br>↓ *Streptococcus* ($p < 0.01$).<br>↓ *Gemella* ($p < 0.001$).<br>↓ *Neisseria* ($p < 0.05$).<br>↓ *Actinomyces* ($p < 0.001$).<br>↓ *Selenomonas 3* ($p < 0.05$).<br>↓ *Haemophilus* ($p < 0.05$).<br>↓ *Bergeyella* ($p < 0.05$).<br>↓ *Stomatobaculum* ($p < 0.05$).<br>↑ *Staphylococcus* ($p < 0.05$).<br>↑ *Mycoplasma* ($p < 0.001$).<br>↑ *Lactobacillus* ($p < 0.01$).<br>↑ *Enterococcus* ($p < 0.001$).<br><br>**ANCOM-BC**<br>**Before Conditioning vs. D+30**<br><br>GCF<br>↓ *Selenomonas 3* ($p < 0.01$).<br>↑ *Mycoplasma* ($p < 0.05$).<br>↑ *Lactobacillus* ($p < 0.05$).<br><br>OM<br>↑ *Lactobacillus* ($p < 0.001$).<br>↑ *Bifidobacteriacea* ($p < 0.001$).<br><br>SB<br>↑ *Lactobacillus* ($p < 0.01$).<br><br>**ANCOM-BC**<br>**Before Conditioning vs. D+75**<br><br>OM<br>↓ *Catonella* ($p < 0.05$). | |
| Lucas 1997[51] 20 | Before Conditioning<br>D+7<br>At Engraftment<br>D+119 | **N/A** | **Before Conditioning vs. Control Group**<br>Patients undergoing allo-HSCT exhibited a significantly higher baseline total aerobic bacterial count compared with controls ($5.35 \times 10^7$ | **N/A** |

| | | | | |
|---|---|---|---|---|
| | | | vs. $1.5 \times 10^7$; $p < 0.008$). No significant differences were found between groups when comparing baseline anaerobic counts (values NR).<br><br>**Before Conditioning vs. D+119**<br>Aerobic count: $5.35 \times 10^7$ vs. $1.9 \times 10^7$; $p$ = NS.<br>Anaerobic count: $6.5 \times 10^7$ vs. $1.9 \times 10^7$; $p$ = NS.<br>*S. mitis*: $3.4 \times 10^5$ vs. $1.0 \times 10^6$; $p$ = NS.<br>*S oralis*: $8.0 \times 10^5$ vs. $5.0 \times 10^5$; $p$ = NS.<br>*S. gordonii* <10 vs. <10, $p$ = NS<br>*S. vestibularis* <10 vs. <10, $p$ = NS<br>*S. salivarius* $7.0 \times 10^5$ vs. 1.30[1,3–7,14] x 10 $^6$, $p$ = NS<br>*S. sanguis* <10 vs. <10, $p$ = NS<br>*S. parasanguis* <10 vs. <10, $p$ = NS<br>*S. mutans* <10 vs. <10, $p$ = NS<br>*S. anginosus* <10 vs. <10, $p$ = NS<br>*S. constellatus* <10 vs. <10, $p$ = NS<br>*S. intermedius* <10 vs. <10, $p$ = NS | |
| Ingham 2021[31]<br>29 | At Preexamination<br>D0<br>D+7<br>D+14<br>D+21<br>D+30<br>D+90<br>D+180<br>D+360 | **All Samples**<br>Lowest Inverse Simpson value at D+21 (details NR). No significant differences were found across all samples. | **Phase I (Preexamination to Conditioning) vs. Phase II (D0 to D+30) vs. Phase III (D+90 to D+360)**<br>Samples from phase I differed from those of phase II. However, phase I samples overlapped with phase III, suggesting a possible recovery of the microbial community.<br><br>**Phase I vs. Phase II**<br>↓ *Actinom*[2,362,36]*ycetaceae* (9.7% vs. 2.9%).<br><br>**Phase I vs. Phase II**<br>↓ *Streptococcaceae* (44.6% vs. 23.3%).<br><br>**All Phases**<br>*Actinomycetaceae, Prevotellaceae,* and *Bacillales Family XI* were more abundant in phase I and phase III samples compared with those from phase II.<br><br>**All Phases (LDA Clade Members)**<br>↑ ASV_18 *Actinomyces sp*. (LDA coefficient = 0.021)<br>↑ ASV_66 *Actinomyces sp*. (LDA coefficient = 0.003)<br>↑ ASV_117 *Actinomyces naeslundii* (LDA coefficient = 0.016)<br>↑ ASV_126 *Actinomyces naeslundii* (LDA coefficient = 0.016)<br>↑ ASV_138 *Actinomyces sp*. (LDA coefficient = 0.003)<br>↑ ASV_155 *Actinomyces odontolyticus* (LDA coefficient = 0.003)<br>↑ ASV_227 *Actinomyces sp*. (LDA coefficient = 0.016)<br>↑ ASV_235 F0332 (LDA coefficient = 0.003)<br>↑ ASV_262 *Actinomyces sp*. (LDA coefficient = 0.003)<br>↑ ASV_345 *Actinomyces sp*. (LDA coefficient = 0.016) | **N/A** |

| | | | | |
|---|---|---|---|---|
| | | | ↑ ASV_389 *Actinomyces odontolyticus* (LDA coefficient = 0.003)<br>↑ ASV_403 *Actinomyces sp.* (LDA coefficient = 0.003)<br>↑ ASV_407 *Actinomyces sp.* (LDA coefficient = 0.016)<br>↑ ASV_2693 *Actinomyces sp.* (LDA coefficient = 0.021)<br>↑ ASV_422 *Actinomyces odontolyticus* (LDA coefficient = 0.003)<br>↑ ASV_431 *Actinomyces sp.* (LDA coefficient = 0.016)<br>↑ ASV_2697 *Actinomyces naeslundii* (LDA coefficient = 0.016)<br>↑ ASV_461 *Actinomyces sp.* (LDA coefficient = 0.003)<br>↑ ASV_475 *Actinomyces gerencseriae* (LDA coefficient = 0.003)<br>↑ ASV_484 *Actinomyces sp.* (LDA coefficient = 0.003)<br>↑ ASV_501 *Actinomyces odontolyticus* (LDA coefficient = 0.003)<br>↑ ASV_516 *Actinomyces odontolyticus* (LDA coefficient = 0.003)<br>↑ ASV_2700 *Actinomyces gerencseriae* (LDA coefficient = 0.003)<br>↑ ASV_568 *Actinomyces sp.* (LDA coefficient = 0.003)<br>↑ ASV_600 *Actinomyces sp.* (LDA coefficient = 0.003)<br>↑ ASV_642 *Actinomyces sp.* (LDA coefficient = 0.003)<br>↑ ASV_798 F0332 (LDA coefficient = 0.003)<br>↑ ASV_871 *Actinomyces massiliensis* (LDA coefficient = 0.003)<br>↑ ASV_2729 *Actinomyces graevenitzii* (LDA coefficient = 0.003)<br>↑ ASV_1055 *Actinomyces sp.* (LDA coefficient = 0.003)<br>↑ ASV_1172 *Actinomyces sp.* (LDA coefficient = 0.003)<br>↑ ASV_2751 *Actinomyces sp.* (LDA coefficient = 0.016)<br>↑ ASV_2664 *Streptococcus sp.* (LDA coefficient = 0.010)<br>↑ ASV_10 *Streptococcus sp.* (LDA coefficient = 0.010)<br>↑ ASV_16 *Streptococcus sp.* (LDA coefficient = 0.010)<br>↑ ASV_27 *Streptococcus sp.* (LDA coefficient = 0.010)<br>↑ ASV_28 *Streptococcus sp.* (LDA coefficient = 0.040)<br>↑ ASV_37 *Streptococcus sp.* (LDA coefficient = 0.010)<br>↑ ASV_48 *Streptococcus sp.* (LDA coefficient = 0.010)<br>↑ ASV_173 *Streptococcus salivarius* (LDA coefficient = 0.010)<br>↑ ASV_183 *Streptococcus sp.* (LDA coefficient = 0.010)<br>↑ ASV_188 *Streptococcus sp.* (LDA coefficient = 0.010)<br>↑ ASV_2674 *Streptococcus sp.* (LDA coefficient = 0.010) | |

| | | | | |
|---|---|---|---|---|
| | | | ↑ ASV_230 *Streptococcus sp.* (LDA coefficient = 0.010)<br>↑ ASV_269 *Streptococcus cristatus* (LDA coefficient = 0.010)<br>↑ ASV_282 *Streptococcus parasanguinis* (LDA coefficient = 0.010)<br>↑ ASV_2683 *Streptococcus mitis* (LDA coefficient = 0.010)<br>↑ ASV_480 *Streptococcus sp.* (LDA coefficient = 0.010)<br>↑ ASV_481 *Streptococcus peroris* (LDA coefficient = 0.010)<br>↑ ASV_802 *Streptococcus sp.* (LDA coefficient = 0.010)<br>↑ ASV_1531 *Streptococcus sp.* (LDA coefficient = 0.010)<br>↑ ASV_1599 *Streptococcus sp.* (LDA coefficient = 0.010)<br>↑ ASV_42 *Prevotella melaninogenica* (LDA coefficient = 0.028)<br>↑ ASV_226 *Prevotella melaninogenica* (LDA coefficient = 0.028)<br>↑ ASV_800 *Prevotella sp.* (LDA coefficient = 0.028)<br>↑ ASV_2665 *Gemella sp.* (LDA coefficient = 0.009)<br>↑ ASV_208 *Gemella sanguinis* (LDA coefficient = 0.009)<br>↑ ASV_2701 *Gemella sp.* (LDA coefficient = 0.009) | |
| Kawajiri 2022[38]<br>10 | D-21<br>D-1<br>D+7<br>D+14<br>D+21 | **N/A** | **All Samples**<br>*Streptococcus* was the most frequently detected genus, present in 90% of patients on oral assessment days.<br><br>**D-21**<br>*Porphyromonas gingivalis* (n = 5/10)<br>*Treponema denticola* (n = 4/10)<br>*Tannerella forsythia* (n = 4/10)<br>*Prevotella intermedia* (n = 6/10)<br>*Fusobacterium nucleatum* (n = 6/10)<br>*Streptococcus species* (n = 9/10)<br>*Streptococcus/Lactobacillus* (n = 4/10)<br><br>**D-1**<br>*Porphyromonas gingivalis* (n = 4/10)<br>*Treponema denticola* (n = 3/10)<br>*Tannerella forsythia* (n = 4/10)<br>*Prevotella intermedia* (n = 2/10)<br>*Fusobacterium nucleatum* (n = 7/10)<br>*Streptococcus species* (n = 8/10)<br>*Streptococcus/Lactobacillu*s (n = 1/10)<br><br>**D+7**<br>*Porphyromonas gingivalis* (n = 3/10)<br>*Treponema denticola* (n = 3/10)<br>*Tannerella forsythia* (n = 5/10)<br>*Prevotella intermedia* (n = 6/10)<br>*Fusobacterium nucleatum* (n = 6/10)<br>*Streptococcus species* (n = 9/10)<br>*Streptococcus/Lactobacillus* (n = 2/10)<br><br>**D+14**<br>*Porphyromonas gingivalis* (n = 5/10)<br>*Treponema denticola* (n = 2/10) | **N/A** |

| | | | | |
|---|---|---|---|---|
| | | | *Tannerella forsythia* (n = 4/10)<br>*Prevotella intermedia* (n = 5/10)<br>*Fusobacterium nucleatum* (n = 5/10)<br>*Streptococcus species* (n = 7/10)<br>*Streptococcus/Lactobacillus* (n = 0/10)<br><br>**D+21**<br>*Porphyromonas gingivalis* (n = 7/10)<br>*Treponema denticola* (n = 3/10)<br>*Tannerella forsythia* (n = 4/10)<br>*Prevotella intermedia* (n = 4/10)<br>*Fusobacterium nucleatum* (n = 6/10)<br>*Streptococcus species* (n = 9/10)<br>*Streptococcus/Lactobacillus* (n = 2/10) | |
| Laheij 2012[32] 49 | Before Conditioning Twice weekly until hospital discharge | **N/A** | **All Samples**<br>*Fusobacterium nucleatum* was the most frequently detected bacterium, present in 86% of patients.<br><br>All samples were positive for bacteria.<br><br>All patients had samples positive for a least one *Candida* species over the allo-HSCT.<br><br>Fusobacterium nucleatum was detected in 86% of patients.<br><br>*Porphyromonas gingivalis* was detected in 20% of patients.<br><br>*Prevotella intermedia* was detected in 18% of patients.<br><br>*Tannerella forsythia* was detected in 48% of patients.<br><br>*Parvimonas micra* was detected in 63% of patients.<br>*Treponema denticola* was detected in 25% of patients.<br><br>*Aggregatibacter actinomycetemcomitans* was detected in 0% of patients.<br><br>*Candida albicans* was detected in 94% of patients.<br><br>*Candida glabrata* was detected in 70% of patients.<br><br>*Candida tropicalis* was detected in 80% of patients.<br><br>*Candida kefyr* was detected in 94% of patients.<br><br>*Candida krusei* was detected in 67% of patients.<br><br>*Candida parapsilosis* was detected in 25% of patients. | **N/A** |
| Laheij 2022[15] 50 | Before Allo-HSCT (8 weeks to days before) | **Before Allo-HSCT vs. D+7**<br>↓ Shannon ($p$ = 0.043). | **All Samples**<br>The composition changed immediately after allo-HSCT, | **N/A** |

| | | | | |
|---|---|---|---|---|
| | Weekly during hospitalization<br>D+90<br>D+180<br>D+360<br>D+450 | **Before Allo-HSCT vs. D+14**<br>↓ Shannon ($p$ = 0.002).<br><br>**Before Allo-HSCT vs. D+21**<br>↓ Shannon ($p$ = 0.000085).<br><br>**Before Allo-HSCT vs. D+90**<br>Shannon was similar ($p$ value non-significant).<br><br>**Before Allo-HSCT vs. D+360**<br>↑ Shannon ($p$ = 0.0024).<br><br>**Before Allo-HSCT vs. D+450**<br>↑ Shannon ($p$ = 0.0029). | remained altered for two to three weeks, returned to pre-treatment conditions by three months, and stayed stable up to 18 months.<br><br>**Hospitalization Samples vs. Others**<br>Samples collected during hospitalization had a lower abundance of:<br>↓ *Lachnospiraceae*<br>↓ *Streptococcus mutans*<br>↓ *Veillonellaceae*<br>↓ *Butyrivibrio*<br>↓ *Peptoniphilaceae*<br><br>Samples collected during hospitalization had a higher abundance of:<br>↑*Prevotella*<br>↑ *Veillonellaceae*<br>↑ *Ralstonia picketti*<br>↑ *Olsenella*<br>↑ *Cryptobacterium curtum* | |
| Ohbayashi 2021[39]<br>96 | At the time of fever<br>D+30 | **N/A** | The most frequently isolated oral microorganism in patients with BSI was coagulase-negative *Staphylococcus species* (47.4%). The second most frequent microorganism was *Enterococcus species* (15.8%). | **N/A** |
| Rashid 2024[40]<br>80 | Before Conditioning<br>cGvHD Onset<br>First cGvHD follow-up visit<br>D+360 | **All samples**<br>No significant difference in Shannon diversity ($p$ = 0.20) | **N/A** | **N/A** |
| Shouval 2019[41]<br>184 | D-7 to D-1<br>D0 to D+6<br>D+7 to D+13<br>D+14 to D+20<br>D+21 to D+34 | **Prior to Allo-HSCT vs. Controls**<br>Oral microbiota diversity in patients prior to transplantation was similar to that of healthy controls, with no significant differences observed (Shannon index, $p$ = 0.460).<br><br>**Prior to Allo-HSCT vs. Peri-engraftment**<br>↓ Shannon ($p$ < 0.001).<br><br>**D-7 to D-1 vs. D+7 to D+13**<br>Pairwise analysis across two-time intervals (D−7 to D−1 and D+7 to D+13) revealed a significantly greater increase in β-diversity among | **N/A** | **N/A** |

| | | | | |
|---|---|---|---|---|
| | | patients with grade 3–4 oral mucositis compared with those with grade 0–1. | | |
| Shouval 2020[43] 184 | Weekly from D-7 to D+34 | **Prior to Allo-HSCT vs. Controls** Oral microbiota diversity in patients prior to transplantation was similar to that of healthy controls, with no significant differences observed (Shannon index, $p$ = 0.460).<br><br>**D+12** A more pronounced reduction in oral microbiota diversity was observed on D+12. | **N/A** | **N/A** |
| Shouval 2020[44] 184 | D-7 to D-1<br>D0 to D+6<br>D+7 to D+13<br>D+14 to D+20<br>D+21 to D+27<br>D+28 to D+34 | **Prior to Allo-HSCT vs. Controls** Baseline saliva samples showed similar α- and β-diversity (measured by weighted UniFrac and Shannon) when compared with healthy controls.<br><br>**All Samples** In patients undergoing allo-HSCT, α-diversity decreased over time, reaching a nadir on D+14.<br><br>**Prior to Allo-HSCT vs. Periengraftment** There was a significant decrease in Shannon diversity and an increase in β-diversity during the periengraftment period ($p$ value NR).<br><br>**Healthy Controls vs. Periengraftment** Periengraftment samples demonstrated a significant reduction in Shannon diversity and increased β-diversity compared with healthy controls ($p$ value not reported). | **Prior to Allo-HSCT vs. Controls** Bacterial composition differed significantly between patients and healthy controls when assessed using linear discriminant analysis effect size.<br><br>**Healthy Donors vs. Prior to Allo-HSCT**<br>↑ *Proteobacteria* ($p$ value NR)<br>↑ *Epsiionproteobacteria* ($p$ value NR)<br>↑ *Campylobacterales* ($p$ value NR)<br>↑*Erysipelotrichales* ($p$ value NR) | **N/A** |

| | | | | |
|---|---|---|---|---|
| Shouval 2020[42] 184 | NR | **Prior to Allo-HSCT vs. Controls**<br>$\alpha$-diversity progressively declined over time, reaching its nadir on day 14 (*p* value NR).<br><br>**All Samples**<br>$\alpha$-diversity decreased over time and reached a nadir on day 14. | **N/A** | **N/A** |
| Soga 2011[45] 63 | D-7 to D-1<br>D0 to D+6<br>D+7 to D+13<br>D+14 to D+20 | **N/A** | **All Samples**<br>Detection frequencies of coagulase-negative staphylococci increased significantly over the course of allo-HSCT, whereas those of bacteria comprising the normal oral flora decreased significantly (Baseline vs. D+7 to D+14 $p < 0.005$; Baseline vs. D+15 to D+21 $p < 0.005$).<br><br>**D-7 to D-1**<br>*$\alpha$-Streptococcus spp*: 54 (91.2%).<br>*$\gamma$-Streptococcus spp*: 11 (19.3%).<br>*Neisseria spp*: 35 (61.4%).<br>*Stomatococcus spp:* 15 (26.3%).<br>*Corynebacterium spp*: 0 (0.0%).<br>Coagulase-negative *Staphylococcus spp*: 8 (14.0%).<br>*Enterococcus spp:* 2 (3.5%).<br>*Pseudomonas aeruginosa*: 1 (1.8%).<br>*Staphylococcus aureus*: 1 (1.8%).<br>*Bacillus spp:* 0 (0.0%).<br>*Stenotrophomonas maltophilia*: 0 (0.0%).<br>*Haemophilus influenzae*: 0 (0.0%).<br>*Enterobacter cloacae*: 2 (3.5%).<br><br>**D0 to D+6**<br>*$\alpha$-Streptococcus spp*: 54 (88.5%).<br>*$\gamma$-Streptococcus spp:* 11 (18.0%).<br>*Neisseria spp:* 35 (57.4%).<br>*Stomatococcus spp*: 14 (23.0%).<br>*Corynebacterium spp*: 2 (3.3%).<br>Coagulase-negative *Staphylococcus spp:* 15 (24.6%).<br>*Enterococcus spp:* 1 (1.6%).<br>*Pseudomonas aeruginosa:* 1 (1.6%).<br>*Staphylococcus aureus*: 2 (3.2%).<br>*Bacillus spp:* 0 (0.0%).<br>*Stenotrophomonas maltophilia*: 0 (0.0%).<br>*Haemophilus influenzae*: 1 (1.6%).<br>*Enterobacter cloacae:* 1 (1.6%).<br><br>**D+7 to D+13**<br>*$\alpha$-Streptococcus spp*: 34 (56.7%).<br>*$\gamma$-Streptococcus spp*: 14 (23.3%).<br>*Neisseria spp:* 35 15 (25.0%).<br>*Stomatococcus spp*: 16 (26.7%).<br>*Corynebacterium spp:* 2 (3.3%).<br>Coagulase-negative *Staphylococcus spp:* 33 (55.0%).<br>*Enterococcus spp*: 3 (5.0%). | **N/A** |

| | | | | |
|---|---|---|---|---|
| | | | *Pseudomonas aeruginosa*: 1 (1.7%).<br>*Staphylococcus aureus:* 1 (1.7%).<br>*Bacillus spp*: 0 (0.0%).<br>*Stenotrophomonas maltophilia*: 1 (1.7%).<br>*Haemophilus influenzae*: 0 (0.0%).<br>*Enterobacter cloacae:* 0 (0.0%).<br><br>**D+14 to D+20**<br>*α-Streptococcus spp*: 35 (74.5%).<br>*γ-Streptococcus spp*: 5 (10.6%).<br>*Neisseria spp*: 8 (17.0%).<br>*Stomatococcus spp:* 14 (29.8%).<br>*Corynebacterium spp*: 1 (2.1%).<br>Coagulase-negative *Staphylococcus spp*: 24 (51.1%).<br>*Enterococcus spp*: 5 (10.6%).<br>*Pseudomonas aeruginosa*: 1 (2.1%).<br>*Staphylococcus aureus*: 0 (0.0%).<br>*Bacillus spp:* 1 (2.1%).<br>*Stenotrophomonas maltophilia*: 1 (2.1%).<br>*Haemophilus influenzae*: 0 (0.0%).<br>*Enterobacter cloacae*: 0 (0.0%). | |
| Lucas 1997[52] 20 | Before Conditioning<br>D+7<br>At Engraftment<br>D+110 to D+130 | **N/A** | **All Samples**<br>**Patients vs. Controls**<br>There was no difference in the mean number of species isolated from either group at baseline or at the end of the study (*p* value NR). There was no significant difference between groups in the total anaerobic counts (*p* value NR). However, there was a significant difference between baseline total aerobic counts between the two groups at baseline ($p < 0.008$).<br><br>**D+7**<br>The predominant species isolated at this time were *Streptococcus mitis* and *Streptococcus oralis*.<br><br>**Before Conditioning vs. D+7**<br>There was a significant decrease in both the total anaerobic count ($p < 0.0002$) and aerobic count ($p < 0.001$).<br>↑ *Streptococcus oralis* (12.1% vs. 48.4%; $p < 0.003$).<br>↓ *Streptococcus salivarius* ($p < 0.008$).<br>↓ *S. sanguis* ($p < 0.04$).<br>↓ *S. parasanguis* ($p < 0.03$).<br>↓ *S. mutans* ($p < 0.07$). | **N/A** |
| Vokurka 2020[46] 22 | Weekly from hospital admission to oral mucositis resolution | **N/A** | **Prior to Oral Mucositis**<br>Potentially pathogenic: 82% (18/22).<br>Pathogenic: 19% (4/22).<br>*Enterococcus faecalis*: 100% (4/4).<br>*Candida glabrata*: 25% (1/4).<br><br>**Oral Mucositis**<br>Negative: 59% (13/22).<br>Potentially pathogenic: 36% (8/22).<br>Pathogenetic: 5% (1/22).<br>*Enterococcus faecalis*: 100% (1/1).<br><br>After Oral Mucositis | **N/A** |

| | | | | |
|---|---|---|---|---|
| | | | Negative: 9% (2/22).<br>Potentially pathogenic: 36% (8/22).<br>Pathogenic: 55% (12/22).<br>*Enterococcus faecalis*: 67% (8/12).<br>*Enterococcus faecium*: 8% (1/12).<br>Vancomycin resistant *Enterococcus*: 8% (1/12).<br>*Candida glabrata*: 17% (2/12).<br>*Candida inconspicua*: 8% (1/12). | |
| Ebadi 2025 [49]<br>47 | Baseline<br>D+14<br>D+28<br>D+84 | In both saliva and supragingival plaque samples, $\alpha$-diversity showed an initial decline from baseline to D+14, followed by recovery until D+84. | **N/A** | **N/A** |
| Faraci 2023[55]<br>17 | Before Allo-HSCT<br>At Engraftment<br>D+30<br>D+100 | **All Samples**<br>Lowest $\alpha$-diversity values occurred at engraftment. | **All Samples**<br>The following taxa showed increased or decreased abundance over time<br>↑ *Firmicutes* (46% to 72%).<br>↑ *Firmicutes/Bacteroidetes* (2.7 to 8.0).<br>↑ *Firmicutes/Proteobacteri*a (2.3 to 7.2).<br>↓ *Proteobacteria* (20% to 10%).<br>↓ *Bacteroidetes* (19% to 17%). | **N/A** |
| Gem<br>2024[50]<br>56 | Baseline<br>D+7<br>D+14<br>D+21<br>D+28<br>D+84 | **All Samples**<br>**Oral Saliva**<br>$\alpha$-diversity declined markedly from baseline to D+14, followed by partial recovery by D+28 and complete recovery by D+84.<br><br>**All Samples**<br>**Supragingival Plaque**<br>$\alpha$-diversity showed a slight decline between baseline and D+14, with no significant changes thereafter. | **Baseline vs. D+14**<br>↓↓ *Oribacterium asaccharolyticum* at D+14 ($p < 0.002$).<br><br>**D+14 vs. D+28**<br>↑↑ *Oribacterium asaccharolyticum* ($p < 0.008$).<br><br>**D+14 vs. D+84**<br>↑↑ *Oribacterium asaccharolyticum* ($p < 0.005$). | **N/A** |

Allo-HSCT = allogeneic hematopoietic stem cell transplantation; ANCOM = Analysis of Composition of Microbiomes; ASV = Amplicon Sequence Variant; BSI = Bloodstream infection; cGvHD = chronic Graft-versus-Host Disease; D = day; FDR = False Discovery Rate; GCF = gingival crevicular fluid; ICU = Intensive Care Unit; LDA = Linear Discriminant Analysis; N = number of patients included in the analysis; N/A = Not Applicable; NR = Not Reported; NS = Not Significant; OM = oral mucosa; SB = supragingival biofilm; * = additional sample was collected if the patient developed respiratory complications within 1 year after allo-HSCT and required intubation; ** = additional samples were obtained if respiratory signs and symptoms developed and patient required hospitalization; *** = additional samples were collected if a patient was admitted to the ICU and intubation occurred and every 48h after that for two additional collections; ↑ = Increased; ↓ = Decreased.
